# Integrated analysis of mRNA-seq and miRNA-seq in the liver of *Pelteobagrus vachelli* in response to hypoxia

**DOI:** 10.1038/srep22907

**Published:** 2016-03-10

**Authors:** Guosong Zhang, Shaowu Yin, Jianqiang Mao, Fenfei Liang, Cheng Zhao, Peng Li, Guoqin Zhou, Shuqiao Chen, Zhonglin Tang

**Affiliations:** 1 College of Life Sciences, Key Laboratory of Biodiversity and Biotechnology of Jiangsu Province, Nanjing Normal University, Nanjing, Jiangsu 210023, China; 2 Co-Innovation Center for Marine Bio-Industry Technology of Jiangsu Province, Lianyungang, Jiangsu 222005, China; 3 Nanjing Institute of Fisheries Science, Nanjing, Jiangsu 210036, China

## Abstract

*Pelteobagrus vachelli* is a well-known commercial species in Asia. However, a sudden lack of oxygen will result in mortality and eventually to pond turnover. Studying the molecular mechanisms of hypoxia adaptation in fishes will not only help us to understand fish speciation and the evolution of the hypoxia-signaling pathway, but will also guide us in the breeding of hypoxia-tolerant fish strains. Despite this, the genetic regulatory network for miRNA-mRNA and the signaling pathways involved in hypoxia responses in fish have remained unexamined. In the present study, we used next-generation sequencing technology to characterise mRNA-seq and miRNA-seq of control- and hypoxia-treated *P. vachelli* livers to elucidate the molecular mechanisms of hypoxia adaptation. We were able to find miRNA-mRNA pairs using bioinformatics analysis and miRNA prediction algorithms. Furthermore, we compared several key pathways which were identified as involved in the hypoxia response of *P. vachelli*. Our study is the first report on integrated analysis of mRNA-seq and miRNA-seq in fishes and offers a deeper insight into the molecular mechanisms of hypoxia adaptation. qRT-PCR analysis further confirmed the results of mRNA-Seq and miRNA-Seq analysis. We provide a good case study for analyzing mRNA/miRNA expression and profiling a non-model fish species using next-generation sequencing technology.

Oxygen serves as the terminal electron acceptor in oxidative phosphorylation. Moreover, several enzymatic processes *in vivo* require molecular oxygen as the direct substrate^1^. Aquatic organisms are usually exposed to oxygen at various concentrations. For example, the natural oxygen level in fresh water can vary widely over the course of 24 hours, from a low level at night to oversaturation during the day. In order to thrive in this environment, fish have adapted and developed various survival strategies (e.g., depression of the metabolic rate, shifting of blood flow mainly to the brain and heart, and efficient production of energy)^2^. Unearthing the molecular mechanisms of hypoxia adaptation in fishes will not only help us to understand fish speciation and the evolution of the hypoxia-signaling pathway but will also guide us in the breeding of hypoxia-tolerant fish strains.

*Pelteobagrus vachelli* has delicious taste with little bone in muscle and high nutritional value. Moreover, it is omnivorous and has a remarkable ability to adapt to environment^3^,^4^. The relatively high yield of *P. vachelli* coupled with an affordable price for consumers thus make it a very popular commercial species in Asia. However, the species is only distributed in some of Asia’s larger rivers, such as the Liaohe, Huaihe, Yangtze, Xiangjiang, Minjiang and Pearl. It is not suitable for high-density pond farming because of the relatively high oxygen-consumption rate and low oxygen threshold; a sudden lack of oxygen will result in mortality among the fish and will eventually lead to pond turnover^5^. These special characteristics of *P. vachelli* suggest that it is not only a significant aquaculture species but also a potential model organism for study of the molecular mechanisms of acute hypoxia. Indeed, no genomic and transcriptomic resources from this species have previously been available, and until now only about 150 EST and 105 protein sequences have been deposited in the NCBI GenBank. This dearth of genetic resources hinders *P. vachelli* molecular breeding as well as further studies on the mechanisms of specific biological processes.

Recently, several species-specific cDNA microarrays have been developed for teleosts, and they are increasingly being used to reveal gene expression patterns in response to hypoxia in fish such as *Gillichthys mirabilis*, *Oryzias latipes* and *Fundulus grandis*^6^,^7^,^8^. These studies have identified several genes that were previously not known to be hypoxia responsive and that can be broadly categorized functionally as protein-coding genes. However, the significance of these observed transcript changes to hypoxia adaptation are far from clear. Previous studies that used various microarray platforms in fish were limited by the large representation of expressed sequence tags (ESTs), which made gene-specific interpretation of data difficult. Moreover, these probes did not include important regulatory elements of the genome such as small RNA (e.g., miRNA). Fortunately, the development of high throughput next-generation sequencing technology (NGST) has effectively overcome this limitation^9^. Rapid advances in NGST have allowed for efficient and economical large-scale production of ESTs. Transcriptome sequencing facilitates functional genomic studies, including global gene expression, novel gene discovery, and assembly of full-length genes^10^. RNA sequencing can also be utilized to analyse the sRNA component of the transcriptome when libraries are prepared from low-molecular weight RNA fractions^11^. This powerful new technology provides an opportunity for studies of species without genome reference databases and of non-model organisms.

MicroRNAs (miRNA) are a class of noncoding RNAs, 18–26 nt in length, that regulate gene expression primarily through post-transcriptional repression or mRNA degradation in a sequence-specific manner^12^. They play an important role in many fundamental cellular processes, including metabolism, cellular proliferation, differentiation, apoptosis, and developmental timing^13^. Under stress conditions, miRNA expression is altered to cope with the stress response. The miRNA regulation of hypoxia-responsive genes has been shown to be a key mechanism of fish in response to hypoxia. Thus, identification of the functional role of miRNA in hypoxia adaptation is an important topic for research in fish biology. In *Oreochromis niloticus*, miR-204 is down-regulated by *in vivo* hypoxia treatments, leading to an induction of its target, vascular endothelial growth factor (VEGF)^14^. Quantitative real-time PCR analysis demonstrated that let-7a, miR-122, and miR-9-3p were down-regulated in the liver and brain of a hypoxic female *Oryzias melastigma*, while miR-2184 was specifically up-regulated in the testis of a hypoxic male^15^. Insights into miRNA-mRNA regulatory networks facilitate understanding of the fine-tuning of gene expression at the post-transcriptional level^16^. However, estimating the regulatory function of miRNA according to the function of mRNA predicted by in silico modeling is not accurate, and those in silico predictions of miRNA-mRNA interactions do not take into account the specific transcriptomic status of the biological system and are biased due to false positives^17^. A potentially more reliable method for predicting miRNA-mRNA relations within a particular biological context is to integrate real mRNA and miRNA transcriptomic data into in silico target predictions^18^.

In the present study, we used NGST to characterise mRNA-seq and miRNA-seq of control- and hypoxia-treated *P. vachelli* livers to elucidate the molecular mechanisms of hypoxia adaptation. We were able to find miRNA-mRNA pairs using bioinformatics analysis and miRNA prediction algorithms. This is the first report on integrated analysis of mRNA-seq and miRNA-seq in fishes and as such offers deeper insight into the molecular mechanisms of hypoxia adaptation. We provide a good case study with which to analyse mRNA/miRNA expression and profile non-model fish species using NGST.

## Results

### Analysis of transcriptome sequencing of *P. vachelli* in response to hypoxia Sequencing and *de novo* assembly

In order to identify mRNA differentiation of *P. vachelli* in response to hypoxia, six cDNA libraries representing the livers of fish in the control group (P0 a, P0 b, P0 c) and those in the experimental group (P4 a, P4 b, P4 c) were constructed with total RNA and subjected to Illumina deep sequencing. Overviews of the sequencing and assembly results for the control group (P0 a, P0 b, P0 c) and experimental group (P4 a, P4 b, P4 c) are shown in Table S1. After discarding the low-quality raw reads, 237,126,362 clean reads remained. Through the Trinity *de novo* assembly method, we obtained 34,744 non-redundant genes, and 46,062 transcripts were achieved with an N50 of 1,744 bp and an average length of 2,086 bp (Table S2). The length distribution of genes and transcripts larger than 200 bp are shown in Figure S1.

### Functional annotation and classification

All the 34,744 assembled genes were referenced against Swiss-Prot, Nr, Pfam, KEGG, KOG, and GO databases, with the number of genes 15,321 (44.10%), 17,955 (51.68%), 14,134 (40.68%), 10,508 (30.24%), 14,444 (41.57%), and 13,604 (39.15%), respectively (Table S3).

According to GO classification system, 13,604 non-redundant genes were classified into three major functional categories (biological process, cellular component, and molecular function) and 50 subcategories (Figure S2). Genes involved in “transcription, DNA-dependent” (1131) and “regulation of transcription, DNA-dependent” (912) groups were notably represented in the biological process category. Among the cellular components, “nucleus” (2,720) was the most commonly represented, followed by “integral to membrane” (2,575) and “cytoplasm” (2,139). In the category of molecular function, a significant proportion of clusters were assigned to “ATP binding” (1,740) and “zinc ion binding” (1,603).

To classify orthologous gene products, 14,444 non-redundant genes were subdivided into 25 KOG classifications. Among them, the cluster of “Signal transduction mechanisms” (2,386, 16.5%) represented the largest group, followed by “General function prediction only” (2,342, 16.2%), “Cell motility” (29, 0.002%) was the smallest group (Figure S3).

The Kyoto Encyclopedia of Genes and Genomes (KEGG) classification was found for 10,508 genes that were further classified into six biochemical pathways (Figure S4). Dominant pathway categories for Organismal Systems (3,785), Metabolism (3,650), Human Diseases (4,005), Genetic Information Processing (917), Environmental Information Processing (2,310) and Cellular Processes (2,817) were Immune System (1,566, 41.4%), Carbohydrate Metabolism (754, 20.7%), Cancers (1,824, 45.5%), Folding, Sorting and Degradation (331, 36.1%), Signal Transduction (1,717, 74.3%), and Cell Communication (1,010, 35.9%), respectively.

### KEGG pathway enrichment analysis of differentially expressed (DE) mRNAs

**Figure 1:**
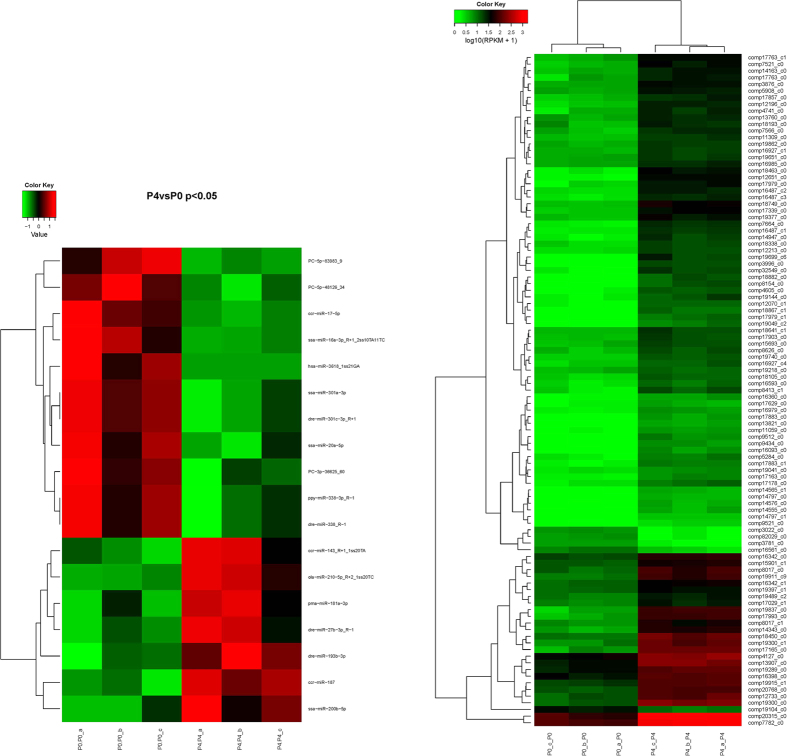
Hierarchical clustering of DE mRNAs and DE miRNAs among six libraries. Heatmap of the count data of DE mRNAs and DE miRNAs libraries for the differentially expressed genes between P0 group and P4 group. Note that only the top 100 genes are included in the DE mRNAs heatmap. For miRNA heatmap all DE miRNAs are included.

To explore the molecular mechanisms of *P. vachelli* in response to hypoxia, RPKM method analysis was performed to determine the DE mRNAs. We found that 712 genes were significantly up-regulated, while 249 genes were significantly down-regulated in response to hypoxia (Table S4). We then made a hierarchical clustering of the DE mRNAs based on the six samples’ log10 (RPKM + 1), with the results indicating that the samples could be sorted into two distinct groups based on hypoxia treatments (Fig. 1). Overall, undergoing hypoxia had a significant impact on the global gene expression profile of *P. vachelli*.

**Figure 2:**
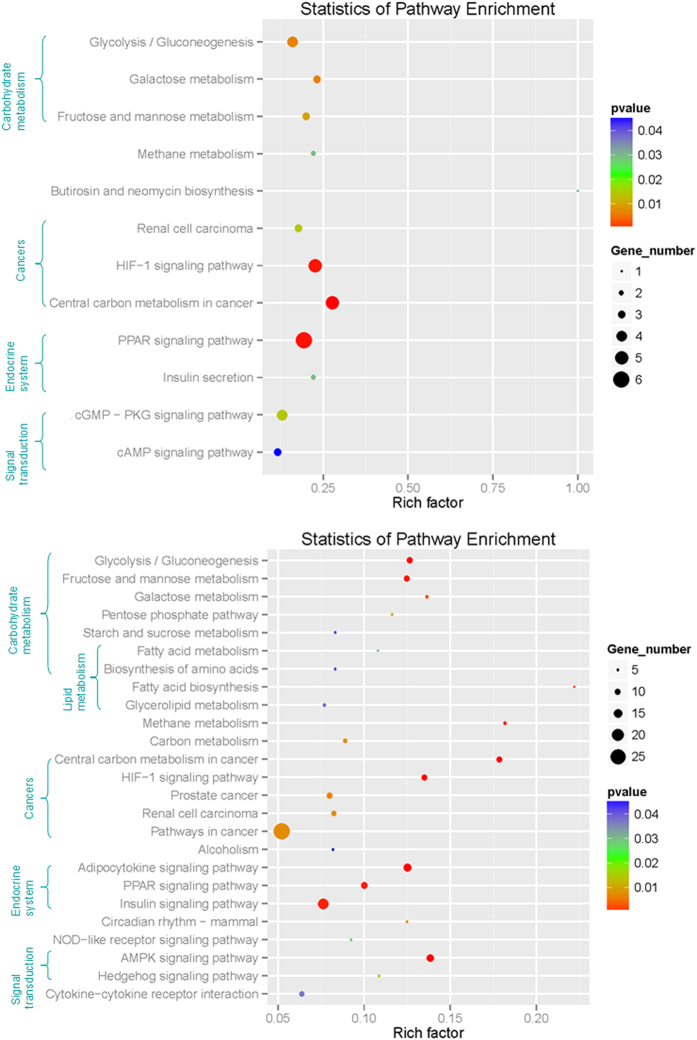
KEGG pathway enrichment analyses of 107 DE unigenes of 162 negative miRNA-mRNA pairs (12 pathways) and 961 DE unigenes of RNA-seq (25 pathways).

By performing the KEGG pathway analyses, a total of 25 pathways which changed significantly (P-value < 0.05) after hypoxia were identified (Fig. 2). Among these pathways, “Carbohydrate metabolism” and “Cancers” were the two most commonly represented subclasses. “Glycolysis/Gluconeogenesis”, “Fructose and mannose metabolism”, “Galactose metabolism”, “Pentose phosphate pathway”, “Starch and sucrose metabolism”, “Fatty acid metabolism” and “Biosynthesis of amino acids” are included in the “Carbohydrate metabolism” subclass. “Central carbon metabolism in cancer”, “HIF-1 signaling pathway”, “Prostate cancer”, “Pathways in cancer” and “Renal cell carcinoma” are included in the “Cancers” subclass. In addition, some important subclasses have also been significantly enriched, including “Lipid metabolism”, “Signal transduction”, “Energy metabolism” and “Endocrine system” (Fig. 2). These results imply that our hypoxia treatment was effective and that the genes involved in these pathways may play crucial roles in the hypoxia response of *P. vachelli*.

### Analysis of miRNA sequencing of *P. vachelli* in response to hypoxia Sequencing and data analysis

In order to identify miRNA differentiation of *P. vachelli* in response to hypoxia, six small RNA libraries representing the livers of fish in the control group (P0 a, P0 b, P0 c) and experimental group (P4 a, P4 b, P4 c) were constructed with total RNA and subjected to Illumina small RNA deep sequencing. After removing contaminant reads, 10.6, 12.0, 10.9, 12.0, 10.5, and 13.3 million clean reads were generated in P0 a, P0 b, P0 c, P4 a, P4 b, and P4 c samples, respectively (Table S5). An overview of reads for sRNA-seq from raw data to high quality and with quality filtering is shown in Table S5. The length distributions of miRNAs were similar among libraries in that 22 nt RNAs were the most abundant (Figure S5), which is consistent with previous reports on fishes, such as *Danio rerio*^19^.

### Identification of conserved and novel miRNAs

In total, we identified 529 conserved miRNAs belonging to 180 miRNA families, and 85 predicted novel miRNAs in the six small RNA libraries (Table S6, S7). Details regarding family member numbers of conserved miRNA are summarized in Table S7. A total of 69 conserved miRNA families contained more than one member.

### Comparison of miRNA expression level among six libraries

Compared with groups of P4 and P0, seven miRNAs were significantly up-regulated, while 11 miRNAs were significantly down-regulated (P < 0.05) (Table S8), and hierarchical clustering of the DE miRNAs based on the six samples’ log10 (Norm) showed the consistency between miRNAs and mRNAs (Figure S6). The results showed that the six samples were sorted into two distinct groups based on hypoxia treatment (Fig. 1).

### Correlation of DE miRNAs and DE mRNAs of *P. vachelli* in response to hypoxia

**Figure 3:**
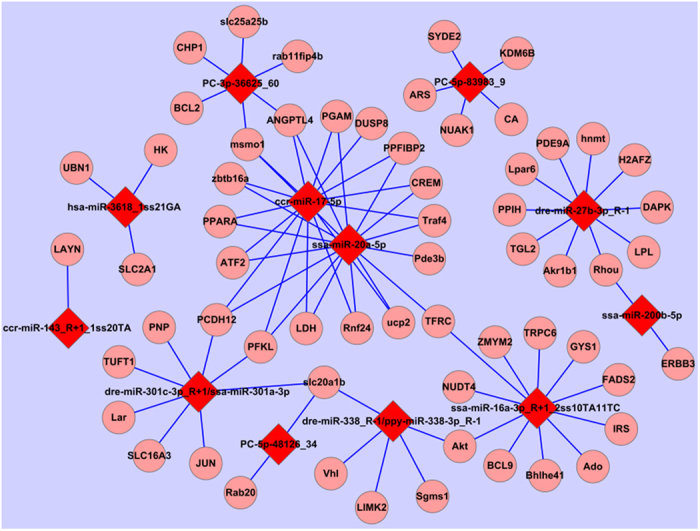
miRNA-mRNA negative correlation network.

DE miRNAs with their predicted target lists were investigated for cognate mRNA targets in their respective DE mRNA list in order to delineate miRNA-mRNA functional interactions using ACGT101-CORR 1.1. There were 308 miRNA-mRNA pairs among treatment groups, with both positive and negative correlation identified (Table S9). Given that miRNAs negatively regulate the expression of their target mRNAs by target RNA cleavage, the expression patterns of miRNA target genes generally show an inverse correlation with those of miRNAs. Therefore, for the majority of cases that involve target cleavage, the simple expectation is that when miRNAs are induced by hypoxia, their target mRNAs are reduced and vice versa. There were 162 negative miRNA-mRNA interactions with the involvement of 18 DE miRNAs and 107 DE mRNAs in total (Table S10). Due to the limited genetic background of *P. vachelli* and that the present ESTs may be the noncoding RNA, our study describes here the interaction between 60 genes with annotation and 14 miRNAs, including 97 negative miRNA-mRNA interactions (Fig. 3). Pathway enrichment analysis for 107 DE mRNAs of 162 negative miRNA-mRNA pairs identified a total of 12 pathways that changed significantly (P-value < 0.05) after hypoxia, with “Carbohydrate metabolism” and “Cancers” the two most frequently represented subclasses (Fig. 2).

### qRT-PCR validation of significant DE miRNAs and DE mRNAs

The expression profiles of 13 DE mature miRNAs (ccr-miR-143_R + 1_1ss20TA, ccr-miR-17-5p, dre-miR-27b-3p_R-1, dre-miR-301c-3p_R + 1, dre-miR-338_R-1, hsa-miR-3618_1ss21GA, ola-miR-210-5p_R + 2_1ss20TC, PC-3p-36625_60, PC-5p-83983_9, ppy-miR-338-3p_R-1, ssa-miR-16a-3p_R + 1_2ss10TA11TC, ssa-miR-20a-5p, and ssa-miR-301a-3p) (Table 1) and 18 DE genes among the miRNA-mRNA interaction network, together with three other DE genes from *P. vachelli*, were further validated using qRT-PCR. Twenty-one DE mRNAs were manually selected as representatives for their potential roles in hypoxia response according to their annotations and their potential relationship with hypoxia-responsive miRNAs. These genes encode 6-phosphofructokinase (miR-17/301c/20a/301a-PFKL), hexokinase (miR-3618-HK), lactate dehydrogenase (miR-17/20a-LDH), phosphoglycerate mutase (miR-17/20a-PGAM), lipoprotein lipase (miR-27b-LPL), apoptosis regulator BCL-2 (PC-3p-36625_60-BCL2), Von Hippel-Lindau disease tumour suppressor (miR-338-Vhl), transferrin receptor (miR-16a/20a-TFRC), MFS transporter, SP family, solute carrier family 2 (facilitated glucose transporter), member 1 (miR-3618-SLC2A1), vascular endothelial growth factor (VEGF), erythropoietin (EPO), death-associated protein kinase (miR-27b-DAPK), transcription factor AP-1 (miR-301a/301c-JUN), angiopoietin-like 4 (miR-17/20a/PC-3p-36625_60-ANGPTL4), carbonic anhydrase (PC-5p-83983_9-CA), 5′-AMP-activated protein kinase, regulatory gamma (PRKAG2), activating transcription factor 2 (miR-17/20a-ATF2, cAMP response element modulator (miR-17/20a-CREM), insulin receptor substrate (miR-16a-IRS), serine-threonine kinase (miR-16a/338-Akt), and dual-specificity phosphatase (miR-17-DUSP8) (Table 2). The results of qRT-PCR revealed that most of these mRNAs/miRNAs share a similar expression tendencies with those from mRNA-Seq/miRNASeq data (RPKM/reads -based expression values) (Tables 1 and 2). Although there were some quantitative differences between the two analytical platforms, the similarities between the RNA-Seq data and the qRT-PCR suggest that the RNA-Seq data are reproducible and reliable.

## Discussion

The findings discussed here reveal the first detailed information regarding parallel mRNA and miRNA expression levels in fish in response to hypoxia. We performed an integrative analysis of these data and obtained the complete set of hypoxia-responsive miRNAs/mRNAs, their interactions, and the dynamics of the biological process, which not only give us deeper insight into the molecular mechanisms of hypoxia adaptation, but also provide a good case study with which to analyse mRNA/miRNA expression and profiling of non-model fish species using NGST.

Some studies have demonstrated that the miRNA-mRNA regulatory network responds to environmental stress, including osmotic stress and temperature^20^. In this work, we attempt to construct the miRNA-mRNA regulatory network according to the DE miRNAs and DE mRNAs datasets and miRNA-targeting information. In total, 308 miRNA-mRNA pairs were identified. Although few cases of positive correlation at the expression level of miRNA and their target mRNAs have been reported, in most cases, the negative correlation between miRNA and their target mRNAs is often considered support for miRNA targeting^18^. In our results, we identified 162 miRNA-mRNA pairs with negative correlation as the key for analysis with the involvement of 18 DE miRNAs and 107 DE mRNAs in total. As shown in Fig. 3, the miRNA-mRNA regulatory network is more complex than previously thought. It is clear that a single miRNA can regulate multiple target mRNAs and vice versa (e.g., comp14576_c0, comp14797_c0, comp17163_c0, comp17214_c0, comp19528_c1, and comp9770_c0 can be simultaneously regulated by dre-miR-301c-3p_R + 1; comp11248_c0 can be simultaneously regulated by PC-3p-36625_60, ccr-miR-17-5p, and ssa-miR-20a-5p).

In performing KEGG pathway analyses for 107 DE mRNAs of 162 negative miRNA-mRNA pairs, “Carbohydrate metabolism” and “Cancers” were found to be the two most frequently represented subclasses (Fig. 2). Similarly, pathway enrichment analysis for 961 DE genes of mRNA-seq showed “Carbohydrate metabolism” and “Cancers” as the two most frequently represented subclasses. In addition, some important subclasses were significantly enriched, including “Lipid metabolism”, “Signal transduction” and “Endocrine system” (Fig. 2). With regard to the hypoxia-adaptation strategies in fish, we addressed our particular research question using pathway analysis to highlight the genes related to the functional clusters (1) Metabolism, (2) Cancers, and (3) Signal transduction. The DE genes and their regulating miRNAs under these three functional clusters were validated using real-time PCR analysis (Tables 1 and 2).

Organisms generally have two different metabolic strategies for coping with the stress of hypoxia—a reduction in metabolic rate and a shift in the aerobic and anaerobic contributions to total metabolism^21^. These DE genes enriched to “Carbohydrate metabolism” and “Endocrine system” (e.g., “Insulin signaling pathway”, “Insulin secretion” and “PPAR signaling pathway”) are primarily involved in blood glucose elevation, glucose utilization, and anabolism reduction. Moreover, “Carbohydrate metabolism” and “Endocrine system” are closely linked^22^. The enrichment of these two subclasses may be necessary to cope with the increased energy demand from the liver in respond to hypoxia, which is needed to re-establish homeostasis. The more severe the hypoxia, the greater the contribution of anaerobic metabolic pathways^23^. The lack of oxygen should increase usage of the glycolytic pathway and decrease use of aerobic pathways^24^. 6-phosphofructokinase (miR-17/301c/20a/301a-PFKL) and hexokinase (miR-3618-HK) are the speed limit of the glycolysis enzyme^25^,^26^. Lactate dehydrogenase (miR-17/20a-LDH) and phosphoglycerate mutase (miR-17/20a-PGAM) are indicators of anaerobic metabolism^27^,^28^. The transcriptional expressions of the key enzymes (PFKL, HK, LDH and PGAM) were significantly up-regulated and their regulating miRNAs were significantly down-regulated, which indicates that these enzymes as well as their regulating miRNAs play an important role in responding to hypoxia. A previous study reported that glycolysis metabolism could compensate for insufficient levels of oxygen in freely diving birds and mammals^29^. The anoxic animals *Trematomus bernacchii*, *Carassius auratus*, and *Hemiscyllium ocellatum* utilized the “Glycolysis/Gluconeogenesis” pathway to maintain active and responsive functions in the absence of oxygen^30^,^31^,^32^. Our results are consistent with the findings of this study. Moreover, “Alcoholism” has also been significantly enriched, revealing that *P. vachelli* can also use ethanol as an anaerobic end product to maintain ion gradients in hepatocytes under hypoxic conditions. This result is consistent with previous reports for *C. auratus* and *Carassius carassius*^33^,^34^,^35^. These lipid metabolism pathways are involved mainly in fat digestion, absorption, and oxidation^36^. There is a fundamental difference in how hypoxia affects the metabolism of lipids in mammals and in fish^37^. In mammals, hypoxia induces an increase in lipid metabolism and inhibits β-oxidation, resulting in an accumulation of fatty acids, which can eventually lead to tissue damage due to elevated plasma fatty acid levels, as with a heart attack^38^. This process does not occur in fishes, due to a reduced lipid metabolism^39^. Lipoprotein lipase (miR-27b-LPL) is a key enzyme for the formation of fatty acids. In our results, miR-27b was significantly up-regulated with its target gene LPL down-regulated, indicating a reduced lipid metabolism in response to hypoxia by *P. vachelli*.

The “Cancers” subclass is mainly involved in the proliferation of cells, inhibiting cell apoptosis and stimulating angiogenesis^40^. It is worth noting that the HIF-1 signaling pathway may offer clues for the molecular adaptation involved in hypoxia tolerance, which plays a pivotal role in the response to hypoxia. As a master regulator of the hypoxia-signaling pathway, the HIF-1 signaling pathway has been preserved through evolution from *Caenorhabditis elegans* to human beings, and it activates a similar or homogenous gene expression, resulting in similar physical and biochemical responses, including oxygen sensing, oxygen transport, angiogenesis, erythropoiesis, and heme metabolism. In this pathway, the apoptosis regulator BCL-2 (PC-3p-36625_60-BCL2), Von Hippel-Lindau disease tumour-suppressor (miR-338-Vhl), transferrin receptor (miR-16a/20a-TFRC), MFS transporter, SP family, solute carrier family 2 (facilitated glucose transporter), member 1 (miR-3618-SLC2A1), VEGF, and erythropoietin (EPO) expression were significantly up-regulated, which is additional evidence of an up-regulated HIF-1 signaling pathway. The death-associated protein kinase (miR-27b-DAPK) is a stress-regulated protein kinase that mediates a range of processes, including signal-induced cell death and autophagy^41^. Transcription factor AP-1 (miR-301a/301c-JUN) is an important transcription factor that regulates the expression of VEGF in response to hypoxic conditions, in turn affecting angiogenesis^42^. Angiopoietin-like 4 (miR-17/20a/PC-3p-36625_60-ANGPTL4) is a member of the angiopoietin family and encodes a secretory glycoprotein that is highly expressed in adipose, liver and placental tissue, as well as in ischemic tissues, which could stimulate angiogenesis *in vitro* and *in vivo*^43^. Carbonic anhydrase (PC-5p-83983_9-CA) is a ubiquitous metalloenzyme that catalyses the reversible hydration/dehydration of carbon dioxide. It is one of the main protein components of red blood cells, ranking second only to hemoglobin. Its expression was found to be regulated under the control of a hypoxia-inducible factor^44^. In *D. rerio*, the expression of CA in the eye, brain, and muscle is significantly up-regulated in response to hypoxia treatment^45^. The identification of these important genes (down-regulated DAPK, up-regulated CA, JUN and ANGPTL4, and vice versa for their regulating miRNAs) suggests the involvement of the liver in the regulated proliferation of red blood cells, inhibiting cell apoptosis and stimulating angiogenesis in fish.

Signal transduction plays a crucial role in fish development and stress response^46^. AMP-activated protein kinase (AMPK) is a serine/threonine kinase that is highly conserved through evolution. As a sensor of cellular energy status, the AMPK system is activated by increases in the cellular AMP:ATP ratio caused by metabolic stresses that either interfere with ATP production (e.g., deprivation of glucose or oxygen) or that accelerate ATP consumption (e.g., muscle contraction). Once activated, AMPK leads to a concomitant inhibition of energy-consuming biosynthetic pathways such as protein, fatty acid, and glycogen synthesis, and activation of ATP-producing catabolic pathways such as fatty acid oxidation and glycolysis. Hypoxia can lead to the activation of AMPK because of the failure to generate sufficient ATP required for cellular functions^47^. Thus, under hypoxic conditions, AMPK initiates various adaptive responses to two different cellular parameters—namely, decreased ATP levels and reduced oxygen levels. Previous studies have reported that hypoxia can affect the activation of the AMPK signaling pathway, demonstrating a potential link between energy status sensing and oxygen availability adaptation in *C. auratus* and *C. carassius*^46^,^48^. In the present study, 5′-AMP-activated protein kinase, regulatory gamma (PRKAG2) was significantly up-regulated, and this increase in activity is possibly associated with inhibition of energy-consuming biosynthetic pathways and activation of ATP-producing catabolic pathways. Activating transcription factor 2 (miR-17/20a-ATF2, also called cAMP response element binding protein 1) and cAMP response element modulator (miR-17/20a-CREM) belong to the alkaline leucine zipper-containing structure (bZIPs) family of transcription factors, mainly in response to Protein kinase A (PKA). They bind to certain DNA sequences called cAMP response elements (CRE), thereby increasing or decreasing the transcription of downstream genes, such as VEGF. These genes are differentially expressed in response to hypoxia in *D. rerio* and *Oncorhynchus mykiss*^49^,^50^. Significantly up-regulated ATF2 and CREM are involved in the AMPK signaling pathway, suggesting an important role in hypoxia adaptation. It has been well established that Insulin receptor substrate (miR-16a-IRS) is an important mediator of insulin and insulin-like growth factor (IGF) signaling. IGF, phosphoinositide 3-kinases (PI3Ks), and the serine/threonine kinase (miR-16a/338-Akt) are the three major members in the IGF/PI3K/Akt signaling pathway^51^. Activation of the IGF/PI3K/Akt pathway increases the hypoxia-inducible factor 1α (HIF-1α) synthesis and transcriptional activity, which amplify a signaling cascade via the HIF-1 signaling pathway^52^. Hypoxia-induced expression of IRS and Akt rise, it means that the up-regulation IGF/PI3K/Akt pathway has participated in a response to hypoxia. MAPKs are a family of enzymes that are involved in oxygen sensing. The MAPK superfamily is made up of three main and distinct signaling pathways: the extracellular signal-regulated protein kinases (ERKs), the c-Jun N-terminal kinases or stress-activated protein kinases (JNK/SAPK), and the p38 family of kinases^53^. The MAPK pathway has been implicated in regulating the HIF-1 signaling pathway, and hypoxia results in a change in MAPK expression in the hearts of *Danio rerio*^54^. Dual-specificity phosphatase (miR-17-DUSP8) can directly dephosphorylate and inactivate each of the MAPK terminal kinases (ERK, p38, JNK), resulting in an induced MAPK pathway^55^. In our results, miR-17 was significantly down-regulated with its target gene DUSP8 significantly up-regulated, providing evidence that MAPK is involved in hypoxia adaptation in fish.

In the present study, compared with groups of P4 and P0, seven miRNAs were significantly up-regulated, while 11 miRNAs were significantly down-regulated. Up to now, the data of small RNAs in the liver of fish are still limited, and information regarding small RNAs in different conditions of dissolved oxygen is especially rare. The miRNA-seq in the livers of *P. vachelli* cultured in different dissolved oxygen conditions is urgently needed. Several recent studies have established a link between hypoxia and the specific target genes of miRNAs in the tumour microenvironment, but there is no direct evidence supporting the involvement of hypoxia adaptation of these miRNAs in fishes. miR-210 was identified as hypoxia inducible in all cell types tested and is overexpressed in most cancer types. Its hypoxic induction is dependent on a functional HIF-1α, which also can be regulated by miR-338 in hepatocarcinoma cells^56^,^57^. VEGF can be regulated by miR-20a under hypoxia in CNE cells^58^. miR-143 can directly target the key glycolytic enzyme hexokinase under hypoxic conditions in lung alveolar epithelial cells^59^. The expression dynamics of these above-mentioned miRNAs is the same as previously reported in the tumour response to hypoxia. Although a given miRNAs in fish may be either predicted (in silico) or demonstrated (*in situ*) to have a regulatory capacity, its physiological relevance has to be established in a specific context within a live system. Characterisation and profiling of miRNAs in different fish is the beginning of a long road, and insights into miRNA-mRNA regulatory networks facilitate the understanding of the fine-tuning of gene expression at the post-transcriptional level.

## Conclusions

**Figure 4:**
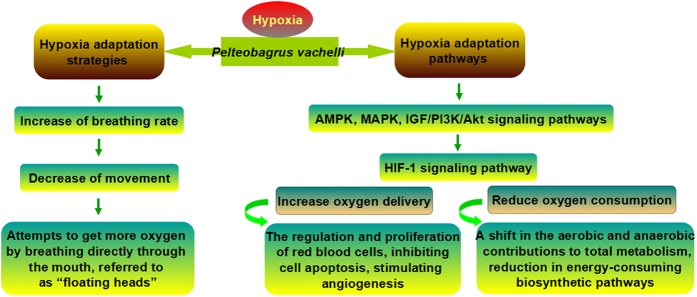
Diagrammatic drawing of hypoxia adaptation strategies and hypoxia adaptation pathways in *P. vachelli*.

To the best of our knowledge, this study is the first exploration to simultaneously characterise the mRNA-seq and miRNA-seq of fish in response to hypoxia. A large number of mRNAs and miRNAs from *P. vachelli* involved in diverse biological pathways were identified. Furthermore, the comparison of several key pathways (e.g., HIF-1 signaling, Glycolysis/Gluconeogenesis, and AMPK signaling pathways) provided informative results which could help us articulate the different mechanisms involved in the hypoxia response of *P. vachelli*. According to our data of integrated analysis, in order to maintain normal physical activity, fishes can have an acute reaction to acute hypoxia, including regulated proliferation of red blood cells, inhibiting cell apoptosis and stimulating angiogenesis, a shift in aerobic and anaerobic contributions to total metabolism, and a reduction in energy-consuming biosynthetic pathways (Fig. 4).

In the present study, we highlighted the negative correlation between miRNA and their target mRNAs, providing another layer of gene regulatory networks in a hypoxia-responsive pathway. Despite the apparent conceptual simplicity of the construction of miRNA-mRNA regulatory networks by integrative analysis of mRNA and miRNA data, the coherent and incoherent relationships between miRNAs and their target mRNAs are complex and dynamic, and the miRNA, transcription factors, or endo-siRNA mediated interactions are not yet well-characterised. Thus, the combination of multilevel high throughput deep sequencing datasets (e.g., Chromatin immunoprecipitation with sequencing data and degradome sequencing data) with bioinformatics analysis could serve as a powerful tool for building better network-based molecular models to predict, test, and identify robust hypoxia-responsive miRNA-mRNA pairs. Moreover, systemic analysis of miRNA-mRNA regulatory network from diverse tissues or cell types and during time courses is required, which would provide a better understanding of gene regulatory network in hypoxia-responsive pathways. Taken together, although this study cannot completely account for the hypoxia-signaling pathway in fish, we provided a good case study to analyse mRNA/miRNA expression and profiling of non-model fish species using NGST.

## Materials and Methods

### Sample collection and RNA extraction

*P. vachelli* (13 ± 1.12 cm in length, 20 ± 1.77 g in weight) were obtained from Nanjing Fisheries Research Institute, Jiangsu Province, China. Then, 120 individuals were randomly transferred to three aquaria with bio-filtered water recirculation systems (equipped with cooling and heating functions and a volume of 200 L and flow rate of 5 L/min) and fed with artificial compound feed, including more than 42.0% protein (Zhenjiang Jiaji Feed Co. Ltd., China). Normal oxygen concentration was 6.8 mg/L (measured with an HQd portable meter and LDO101 probe). After acclimation at 25 ± 1 °C, PH 7.2 ~ 7.4 for two weeks and sequential feed restriction for two days, the fish were used for the experiments.

First, we tested the oxygen threshold for *P. vachelli.* While the water was deoxygenated for 34 min by bubbling pure nitrogen gas and the oxygen concentration was lower than 0.61 mg/L, *P. vachelli* tried to get more oxygen by breathing directly through the mouth, which is often referred to as “floating heads.” Thus, we chose 0.7 mg/L as the oxygen concentration level for creating a hypoxic condition for this study. Control fish (P0 a, P0 b, and P0 c) were removed from three aquaria for immediate liver dissection. Next, the oxygen infiltration and recirculation systems in the three aquaria were closed to initiate the hypoxia experiments. The water was deoxygenated for 30–35 min by bubbling pure nitrogen gas in order to decrease oxygen concentration from 6.8 mg/L to 0.7 mg/L. After oxygen concentration was maintained for 4 h by continuous bubbling of nitrogen gas, the experimental fish (P4 a, P4 b, and P4 c) were quickly removed for liver dissection. Samplings of control fish (P0 a, P0 b, and P0 c) and experimental fish (P4 a, P4 b, and P4 c) had three biological replicates, each made up of five different individual liver tissues. Fish were killed by dissection after mild anaesthetisation in a eugenol bath (1:10,000), after which they were frozen in liquid nitrogen and stored at −80 °C. All experiments were performed according to the Guidelines for the Care and Use of Laboratory Animals in China. This study was also approved by the Ethics Committee of Experimental Animals at Nanjing Normal University.

Total RNA was extracted using Trizol reagent (Invitrogen, Carlsbad, CA, USA) following the manufacturer’s protocol. The total RNA quantity and purity were analysed with Bioanalyzer 2100 and RNA 6000 Nano LabChip Kit (Agilent, CA, USA) with RIN number > 7.0.

### Transcriptome sequencing, assembly, and annotation

For six cDNA library constructions, approximately 5 μg of total RNA per sample was used for the RNA sample preparations. The library for sequencing was generated using an Illumina TruSeq RNA Sample Preparation Kit (Illumina, San Diego, CA, USA). Transcriptome sequencing was carried out on an Illumina HiSeq 2500 platform that generated approximately 125-bp paired-end (PE) raw reads by LC Sciences (Houston, TX, USA). After removing adaptor sequences, ambiguous ‘N’ nucleotides (with the ratio of ‘N’ greater than 5%) and low quality sequences (with quality score less than 20), the remaining clean reads were assembled using trinity software^60^ as described for *de novo* transcriptome assembly without a reference genome. The quality of the assembly was critically assessed by LC Sciences before subsequent analysis.

For homology annotation, non-redundant sequences were annotated based on the following databases: Swiss-Prot (a manually annotated and reviewed protein sequence database); Nr (NCBI non-redundant protein sequences); Pfam (protein family); KEGG (KEGG Ortholog database); and KOG (euKaryotic Ortholog Groups). All the genes were searched against Nr, KEGG, Swiss-Prot, Pfam (protein family) and KOG databases using the BLASTx algorithm (E-value < 1E-5). If the results from different databases conflicted, a priority order of alignments from the Nr, KEGG, Swiss-Prot, Pfam, and KOG databases was followed.

### Analysis of differentially expressed (DE) mRNAs

The expression level of each transcript was measured as the number of clean reads mapped to its sequence. The mapped clean read number was normalized to RPKM (reads per kilo of per million mapped reads) with RSEM 1.2.3. We used DESeq to determine the FDR threshold. FDR < 0.05 and fold change > 2 were considered to indicate significant expression abundance. KEGG is the major public pathway-related database for helping to further understand the biological functions of the high level functions of genes as well as the utilities of the biological system of large-scale molecular datasets (http://www.genome.jp/kegg/).

Pathway enrichment analysis identifies significantly enriched metabolic pathways or signal transduction pathways using the corrected P-value < 0.05 as a threshold to find significantly enriched KEGG terms in the input list of DE genes, comparing them to the whole genome background. The calculation formula of P-value was as follows:


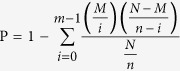


N represented the number of KEGG annotated genes in *P. vachelli*, n represented the number of differentially expressed genes in N, M represented the number of particular KEGG annotated genes in a genome, and m represented the number of particular KEGG annotated genes expressed differentially in M. After correction for multiple testing, we chose pathways with a P-value < 0.05 to represent those significantly enriched in DE genes.

### Small RNAs sequencing and analysis

Approximately 2.5 μg of total RNA were used to prepare small RNA library according to the protocol of TruSeq Small RNA Sample Prep Kits (Illumina, San Diego, CA, USA). And then the libraries were sequenced by Illumina Hiseq2500 50SE (single end) at the LC-BIO (Hangzhou, China) following the vendor’s recommended protocol. Data processing followed the procedures as described in a previous study. Briefly, the raw reads were subjected to the Illumina pipeline filter (Solexa 0.3), and then the dataset was further processed with an in-house program, ACGT101-miR (LC Sciences, Houston, TX, USA), to remove adapter dimers, junk, low complexity, common RNA families (rRNA, tRNA, snRNA, snoRNA), and repeats.

Subsequently, unique sequences 18–26 nt in length were mapped to specific species precursors in miRBase 21.0 by Bowtie search to identify known miRNAs and novel 3p- and 5p- derived miRNAs. Length variation at both 3′ and 5′ ends and one mismatch inside of the sequence were allowed in the alignment. Mapping methods for identification of conserved and novel miRNAs are listed in Table S6.

### Analysis of DE miRNAs

miRNA differential expression based on normalized deep-sequencing counts was analysed by selectively using Student t test based on the experimental design. The significance threshold was set to be 0.05 in this test.

### Prediction of Target Genes of miRNAs

To predict the genes targeted by DE miRNAs, two computational target prediction algorithms (TargetScan 50 and miRanda 3.3a) were used to identify miRNA binding sites. Finally, the data predicted by both algorithms were combined and the overlaps were calculated.

### Integrated analysis of mRNA-seq and miRNA-seq

In order to define all the possible miRNA-mRNA interactions, including positive and negative relationships between miRNA and mRNA expression, we used ACGT101-CORR 1.1 to construct the miRNA-mRNA regulatory network. Briefly, we normalized all the sample-matched miRNA and mRNA sequencing data. Afterwards, integration of miRNA-seq with mRNA-seq was achieved by integrating expression profiles of DE miRNAs and DE mRNAs with the addition of DE miRNA-targeting information.

### Gene expression validation

The expression profiles of 13 DE mature miRNAs and 18 DE genes among the miRNA-mRNA interaction network, together with three other DE genes from *P. vachelli* were further validated using qRT-PCR.

The relative expression of 11 known and two novel miRNAs were selected and analysed by quantifying the miRNA stem-loop. Total RNAs were isolated using Trizol reagent (Biotake, Beijing, China), following the cDNA generation using 1 μg of total RNA by reverse transcription kit (Toyobo, Osaka, Japan). Quantitative real-time PCR (qRT-PCR) was performed on an ABI Step One Plus system (Applied Biosystems, Foster, CA, USA) by using qRT-PCR Reagents provided by Toyobo. The stem-loop primers are shown in supplementary data (Table S11). PCR amplification was conducted under an initial denaturation at 94 °C for 30 seconds (s), and then 40 cycles of amplification including the denaturation at 94 °C with 20 s, annealing at 61 °C for 30 s, and extension at 72 °C for 30 s; after 40 cycles was final extension at 72 °C for 1 minute. Each sample was tested in triplicate. Finally, the melting curve was performed to verify the specificity of PCR amplification. U6 was used as an internal control.

To verify RNA-seq results, qRT-PCR method with β-actin as an internal control was used to explore mRNA expression levels. qRT-PCR was performed with an SYBR Green Master kit according to the manufacturer’s protocol (Roche, Basel, Switzerland). The primers for qRT-PCR are listed in Table S11. The experiments were carried out in triplicate with a total volume of 20 μL in ABI stepone^TM^ plus (Applied Biosystems, Waltham, MA, USA), containing 10 μL of SYBR green master, 4 μL of cDNA (500 ng), and 3 μL of forward and reverse primers (2 μmol/L). The qRT-PCR was programmed at 95 °C for 10 min, followed by 40 cycles of 95 °C for 15 s, and 55 °C for 1 min. The expression level was calculated by 2^−ΔΔCT^ method and subjected to statistical analysis. During mRNA expression analysis, fold-change was determined by comparing with the reference gene expression.

## Additional Information

**How to cite this article**: Zhang, G. *et al.* Integrated analysis of mRNA-seq and miRNA-seq in the liver of *Pelteobagrus vachelli* in response to hypoxia. *Sci. Rep.* **6**, 22907; doi: 10.1038/srep22907 (2016).

## Supplementary Material

Supplementary Information

## Figures and Tables

**Table 1:** Relative miRNA expression of 13 selected DE genes for comparison of the P4 versus P0 groups, in respect to miRNA-Seq and Quantitative real-time PCR.

miR_name	Illumina miRNA-seq (log2 fold change)	Regulation (P4 vs P0)	Real-time PCR (log2 fold change)
ola-miR-210-5p_R + 2_1ss20TC	2.28	up	1.94*
ccr-miR-17-5p	−0.62	down	−0.76*
dre-miR-301c-3p_R + 1	−0.95	down	−1.76*
ssa-miR-16a-3p_R + 1_2ss10TA11TC	−1.09	down	−1.04*
PC-5p-83983_9	−1.93	down	−1.72*
ssa-miR-20a-5p	−0.73	down	−1.63*
dre-miR-338_R-1	−0.52	down	−1.36*
ccr-miR-143_R + 1_1ss20TA	0.40	up	0.49*
PC-3p-36625_60	−0.98	down	−0.93*
hsa-miR-3618_1ss21GA	-inf	down	-inf *
dre-miR-27b-3p_R-1	0.57	up	−0.59*
ppy-miR-338-3p_R-1	−0.52	down	−1.36*
ssa-miR-301a-3p	−0.95	down	−1.76*

^*^Asterisk indicates statistical significance of differential gene expression with p-value < 0.05 (t-test). fold change = P4 group (mean)/P0 group(mean). “mean” represents the mean of three biological replicates, respectively.

**Table 2:** Relative mRNA expression of 21 selected DE genes for comparison of the P4 versus P0 groups, in respect to mRNA-Seq and Quantitative real-time PCR.

annotation	Accession	Illumina mRNA-seq (log2 fold change)	regulation	Real-time PCR (log2 fold change)
Metabolism				
6-phosphofructokinase (PFKL)	comp19528_c1	1.50	up	2.00*
hexokinase (HK)	comp19211_c0	1.77	up	3.03*
lactate dehydrogenase (LDH)	comp5908_c0	2.75	up	3.62*
phosphoglycerate mutase (PGAM)	comp13915_c0	1.69	up	1.91*
lipoprotein lipase (LPL)	comp18665_c0	−1.31	down	−1.08*
Cancers				
vascular endothelial growth factor (VEGF)	comp17707_c0	0.95	up	1.23*
erythropoietin(EPO)	comp7521_c0	3.45	up	4.08*
apoptosis regulator BCL-2(BCL2)	comp12482_c0	1.53	up	1.09*
von Hippel-Lindau disease tumor supressor(Vhl)	comp16131_c0	1.37	up	1.84*
transferrin receptor(TFRC)	comp19840_c0	1.44	up	1.09*
MFS transporter, SP family, solute carrier family 2 (facilitated glucose transporter), member 1(SLC2A1)	comp19218_c0	1.99	up	1.45*
death-associated protein kinase (DAPK)	comp17281_c0	−1.45	down	−1.46*
transcription factor AP-1(JUN)	comp9770_c0	2.69	up	2.63*
angiopoietin-like 4(ANGPTL4)	comp13907_c0	2.49	up	3.19*
carbonic anhydrase (CA)	comp12230_c0	1.66	up	1.19*
Signal transduction				
activating transcription factor 2(ATF2)	comp18509_c0	3.67	up	2.96*
cAMP response element modulator(CREM)	comp16593_c0	2.54	up	2.34*
insulin receptor substrate(IRS)	comp18226_c0	2.61	up	2.15*
dual specificity phosphatase (DUSP8)	comp16360_c0	2.51	up	2.32*
serine/threonine kinase (Akt)	comp18391_c0	2.50	up	2.10*
5′-AMP-activated protein kinase, regulatory gamma subunit(PRKAG2)	comp19753_c1	1.14	up	1.52*

^*^Asterisk indicates statistical significance of differential gene expression with p-value < 0.05 (t-test). fold change = P4 group (mean)/P0 group(mean). “mean” represents the mean of three biological replicates, respectively.
