# Supplementary material for: Integrated analysis of mRNA-seq and miRNA-seq in the liver of Pelteobagrus vachelli in response to hypoxia
Source: Sci Rep. 2016 Mar 10;6:22907. doi: 10.1038/srep22907 (PMC4785494; doi:10.1038/srep22907)
Supplement: Supplementary Information [file srep22907-s1.doc]

**Integrated analysis of mRNA-seq and miRNA-seq in the liver of *Pelteobagrus vachelli* in response to** **hypoxia**

**Guosong Zhang^a,b^, Shaowu Yin^a,b*^, Jianqiang Mao^c^, Fenfei Liang^a,b^, Cheng Zhao^a,b^, Peng Li^a,b^, Guoqin Zhou^c^, Shuqiao Chen^c^, Zhonglin Tang^c^**

**^a^**College of Life Sciences, Key Laboratory of Biodiversity and Biotechnology of Jiangsu Province, Nanjing Normal University, Nanjing, Jiangsu 210023, China

**^b^**Co-Innovation Center for Marine Bio-Industry Technology of Jiangsu Province, Lianyungang, Jiangsu 222005, China

**^c^**Nanjing Institute of Fisheries Science, Nanjing, Jiangsu 210036, China

Figure S1 Distribution of the assembled genes and transcript length.


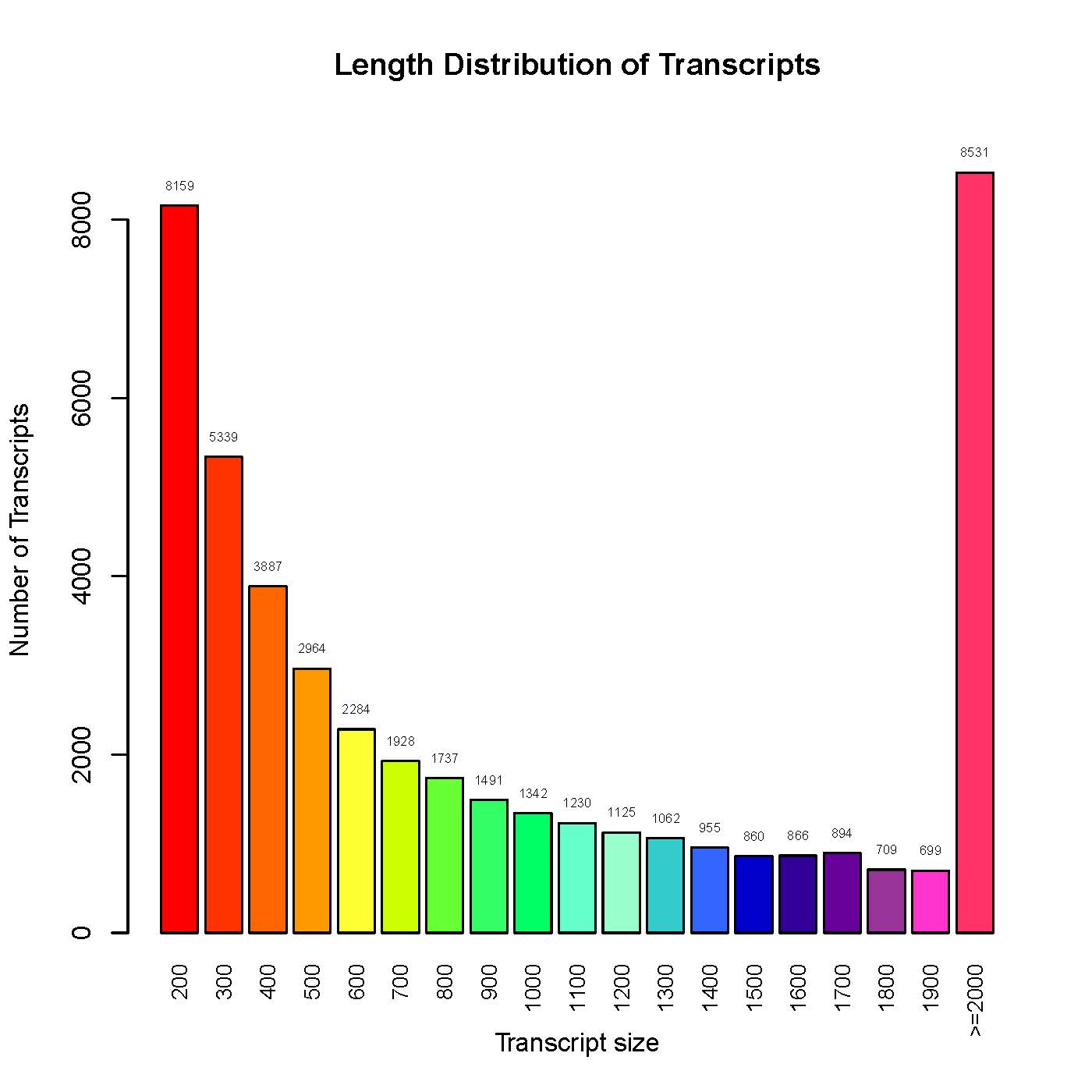

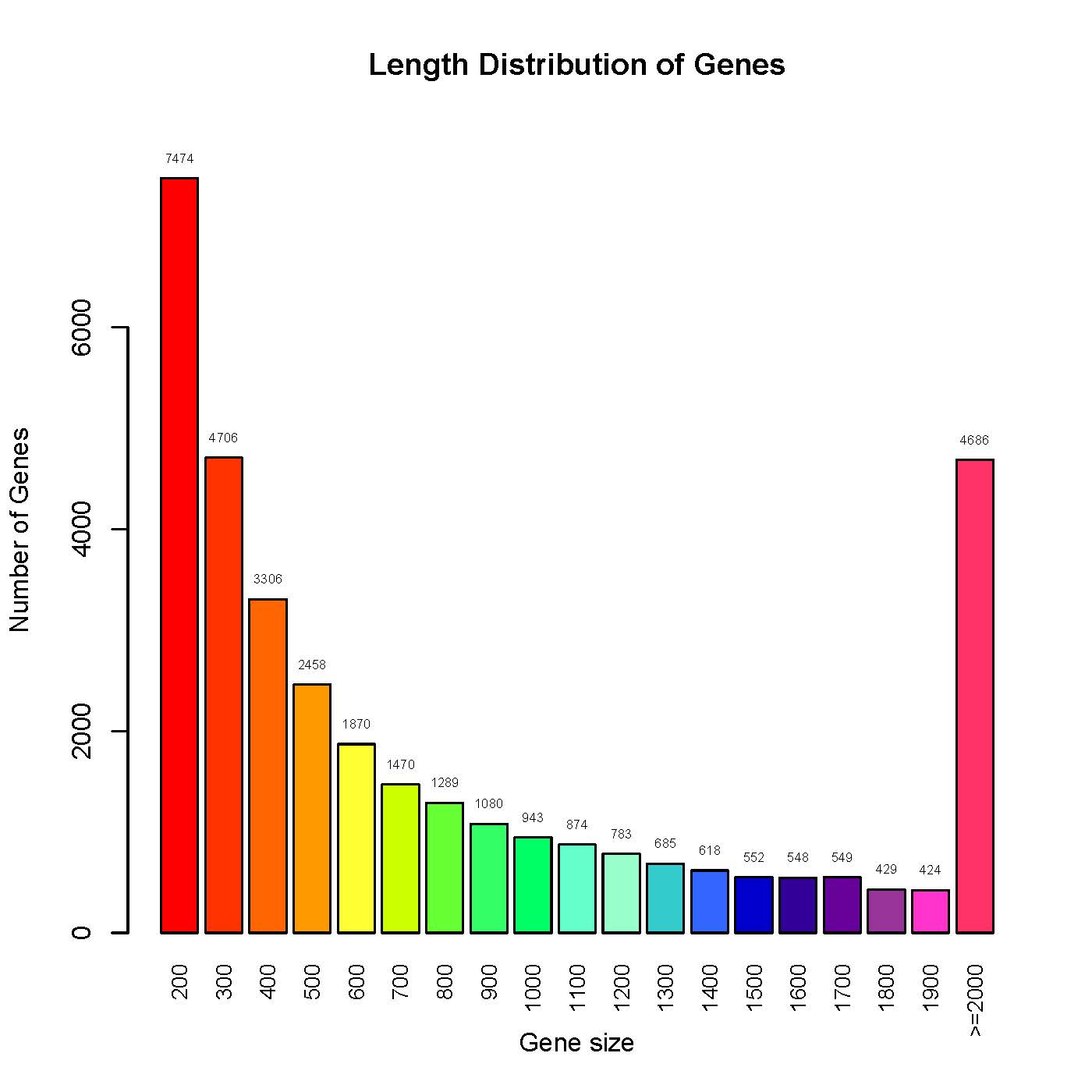


Figure S2 GO categorization of non-redundant unigenes.


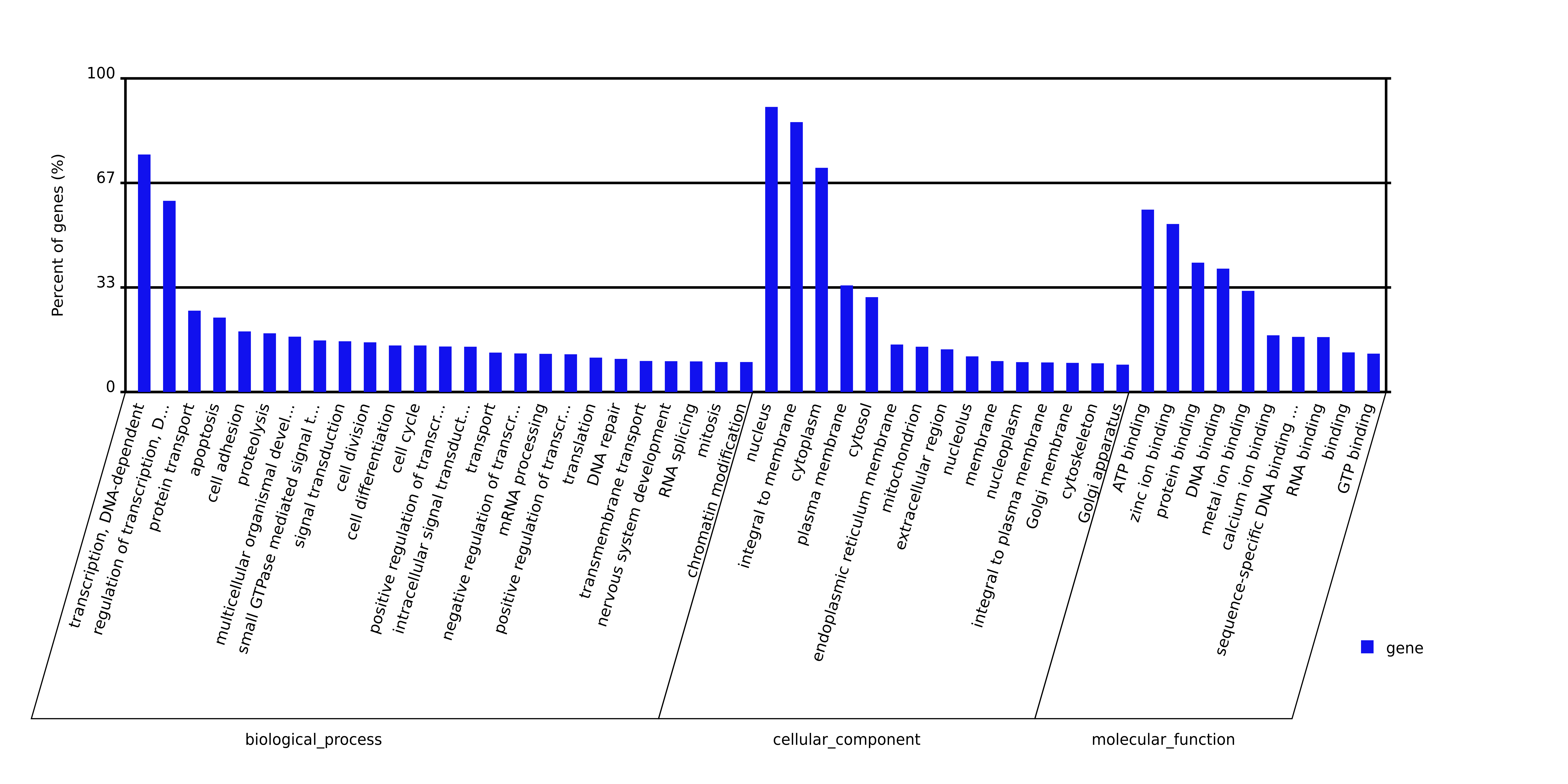


Figure S3 KOG annotation of putative proteins.


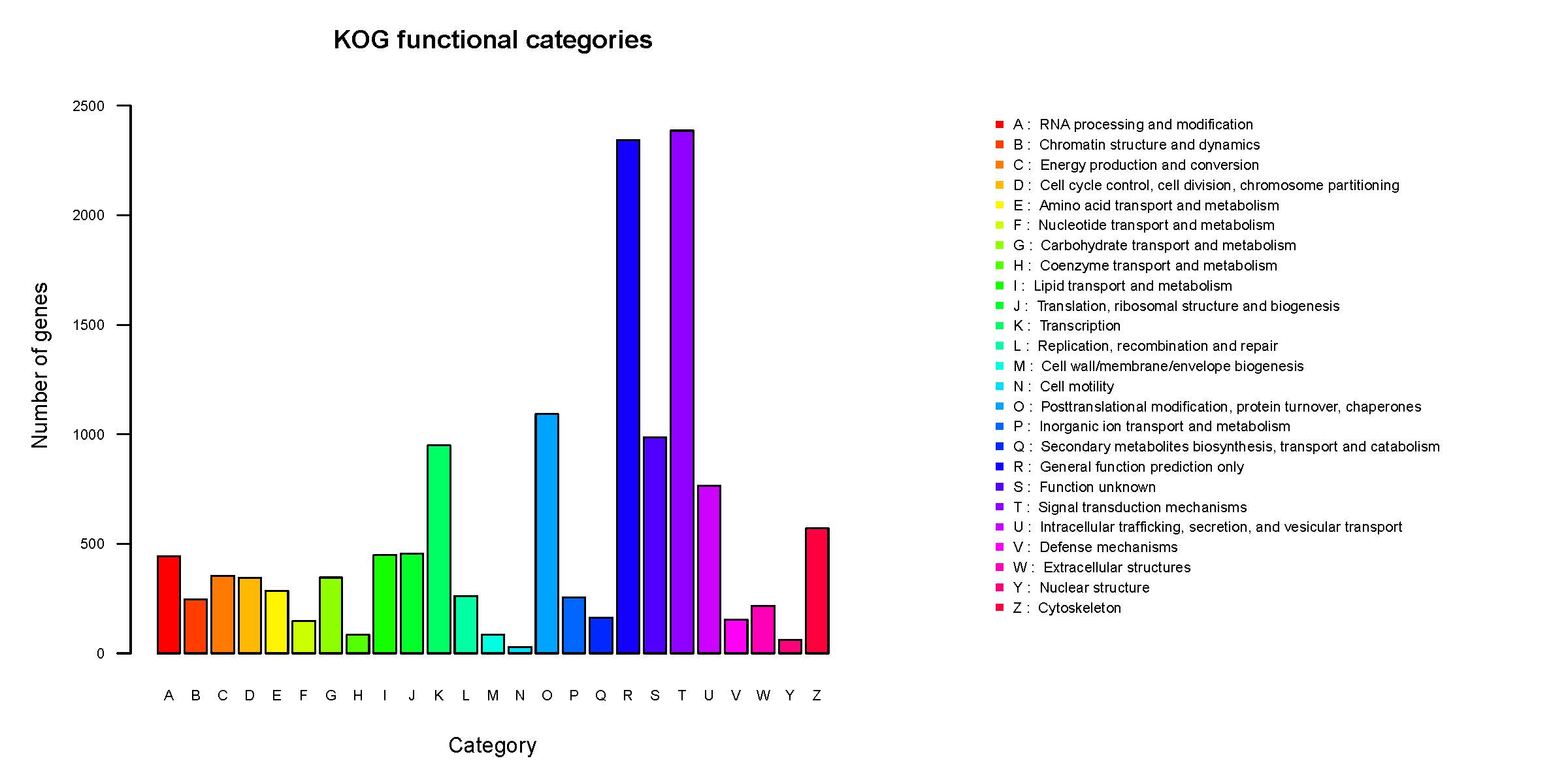


Figure S4 KEGG pathways of differentially expressed genes.


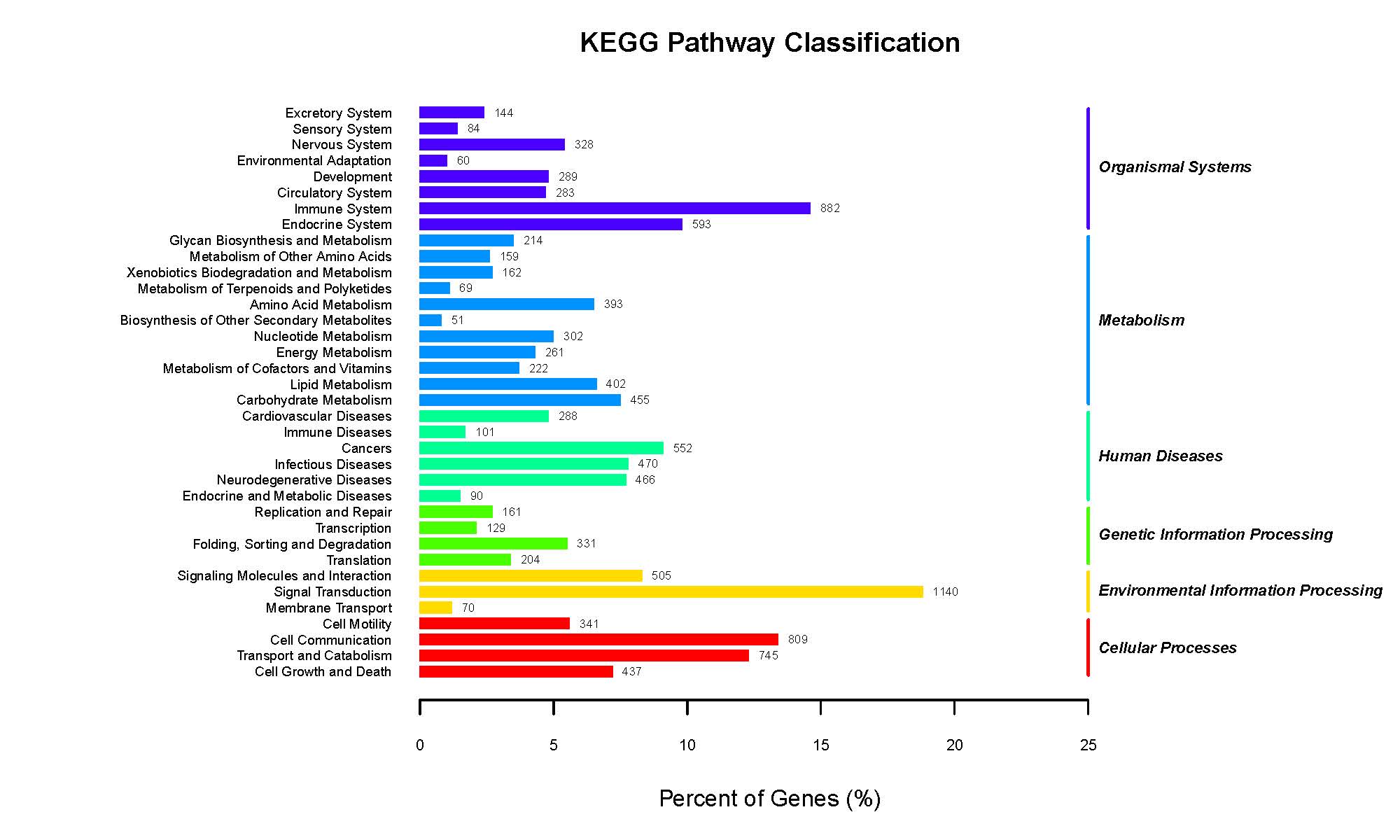


Figure S5 The length distribution of the small RNA in six miRNA libraries.


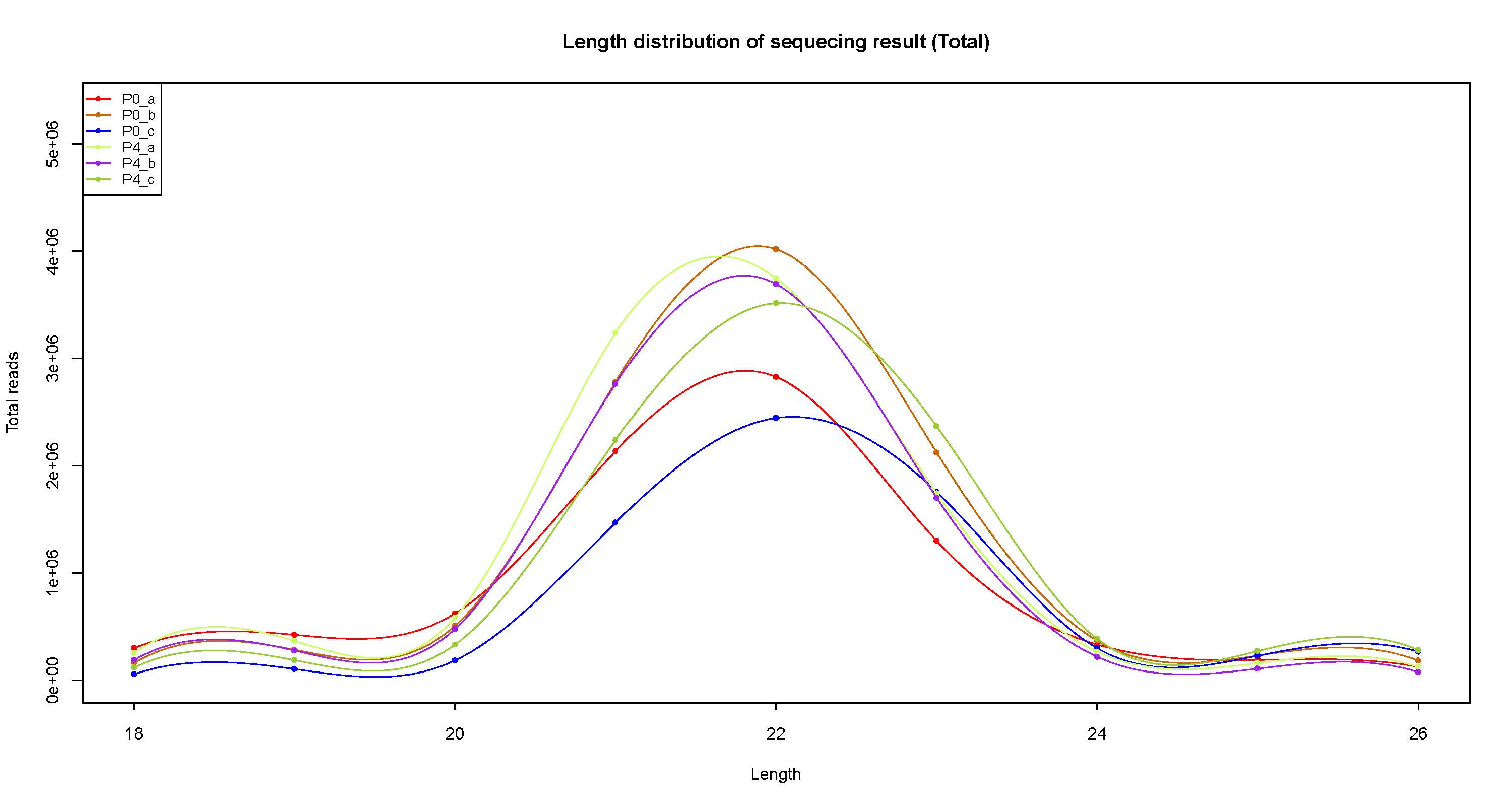


Table S1 Summary of sequence data generated for *P. vachelli* transcriptome, and quality filtering

| Sample | Raw Data | Valid Data | Valid% | Q20% | Q30% | GC% |
| --- | --- | --- | --- | --- | --- | --- |
| P0 a | 39126322 | 38780204 | 99.12 | 92.26 | 85.24 | 47.61 |
| P0 b | 40354078 | 40127532 | 99.44 | 93.25 | 86.85 | 48.27 |
| P0 c | 39203638 | 38859272 | 99.12 | 89.60 | 81.29 | 47.00 |
| P4 a | 39421990 | 39054098 | 99.07 | 92.40 | 85.48 | 47.91 |
| P4 b | 42331220 | 41972364 | 99.15 | 92.83 | 86.19 | 47.89 |
| P4 c | 38661974 | 38332892 | 99.15 | 89.70 | 81.29 | 47.24 |

Table S2 Assembly statistics of reads.

|  | All | Min Length | Mean Length | Max Length | Total Assembled bases | N50 |
| --- | --- | --- | --- | --- | --- | --- |
| gene | 34744 | 201 | 1016 | 17111 | 35312747 | 1744 |
| transcript | 46062 | 201 | 1232 | 17111 | 56759524 | 2086 |

Table S3 Blast analysis of non-redundant unigenes against public databases.

| gene_number | swiss-prot | nr | Pfam | KEGG | KOG | GO |
| --- | --- | --- | --- | --- | --- | --- |
| 34744 | 15321 | 17955 | 14134 | 10508 | 14444 | 13604 |
| 100% | 44.10% | 51.68% | 40.68% | 30.24% | 41.57% | 39.15% |

Table S4 List of the genes up-regulated or down-regulated in response to hypoxia

| gene_ID | Annotation | mean_rpkm_P4 | mean_rpkm_P0 | log2fold_change | pvalue |
| --- | --- | --- | --- | --- | --- |
| comp12651_c0 | FK506-binding protein 1 | 36.81 | 0.87 | 5.33 | 0.00 |
| comp17165_c0 | hypoxia-inducible factor prolyl hydroxylase | 144.25 | 5.22 | 4.73 | 0.00 |
| comp19837_c0 | nuclear receptor, subfamily 1, group C, member 1 | 88.37 | 3.80 | 4.48 | 0.00 |
| comp17993_c0 | - | 81.89 | 3.66 | 4.44 | 0.00 |
| comp18463_c0 | - | 33.95 | 1.17 | 4.82 | 0.00 |
| comp20315_c0 | - | 1683.62 | 89.35 | 4.16 | 0.00 |
| comp17979_c0 | mitochondrial carrier protein, MC family | 31.68 | 1.52 | 4.33 | 0.00 |
| comp19300_c0 | - | 196.95 | 12.09 | 3.98 | 0.00 |
| comp18749_c0 | - | 45.90 | 2.65 | 4.06 | 0.00 |
| comp17339_c0 | protein-tyrosine phosphatase | 38.30 | 2.40 | 3.94 | 0.00 |
| comp19300_c1 | - | 126.48 | 6.66 | 4.20 | 0.00 |
| comp14343_c0 | cysteamine dioxygenase | 60.60 | 4.05 | 3.84 | 0.00 |
| comp7664_c0 | - | 21.93 | 1.14 | 4.19 | 0.00 |
| comp12196_c0 | - | 25.89 | 2.22 | 3.49 | 0.00 |
| comp3996_c0 | - | 14.78 | 0.36 | 5.29 | 0.00 |
| comp18867_c1 | prolyl 4-hydroxylase | 9.50 | 0.65 | 3.81 | 0.00 |
| comp16487_c1 | - | 19.07 | 1.24 | 3.88 | 0.00 |
| comp4605_c0 | - | 12.21 | 0.50 | 4.61 | 0.00 |
| comp17763_c1 | - | 36.63 | 3.82 | 3.22 | 0.00 |
| comp14797_c0 | MFS transporter, MCP family, solute carrier family 16 (monocarboxylic acid transporters), member 3 | 3.28 | 0.12 | 4.74 | 0.00 |
| comp12213_c0 | - | 15.83 | 1.65 | 3.21 | 0.00 |
| comp7521_c0 | erythropoietin | 34.04 | 3.01 | 3.45 | 0.00 |
| comp3876_c0 | aspartate beta-hydroxylase | 33.56 | 3.97 | 3.02 | 0.00 |
| comp18450_c0 | - | 141.27 | 8.67 | 3.96 | 0.00 |
| comp19915_c1 | - | 115.10 | 18.38 | 2.87 | 0.00 |
| comp16487_c2 | - | 28.05 | 1.69 | 3.98 | 0.00 |
| comp20768_c0 | GABA(A) receptor-associated protein (autophagy-related protein 8) | 102.81 | 13.39 | 2.88 | 0.00 |
| comp14163_c0 | fructose-bisphosphate aldolase, class I | 29.95 | 3.37 | 3.10 | 0.00 |
| comp14576_c0 | protein-tyrosine phosphatase | 3.68 | 0.22 | 4.01 | 0.00 |
| comp17763_c0 | Cu2+-exporting ATPase | 29.19 | 3.55 | 3.00 | 0.00 |
| comp18338_c0 | hypoxia-inducible factor prolyl hydroxylase | 15.69 | 1.93 | 2.95 | 0.00 |
| comp14947_c0 | - | 17.81 | 0.99 | 4.09 | 0.00 |
| comp13821_c0 | adenosine receptor A2b | 4.22 | 0.37 | 3.45 | 0.00 |
| comp5908_c0 | L-lactate dehydrogenase | 28.87 | 4.09 | 2.75 | 0.00 |
| comp18882_c0 | - | 12.73 | 0.45 | 4.77 | 0.00 |
| comp14565_c1 | 6-phosphofructokinase | 3.51 | 0.21 | 4.02 | 0.00 |
| comp3781_c0 | - | 0.36 | 4.67 | -3.82 | 0.00 |
| comp9434_c0 | hypoxia-inducible factor prolyl hydroxylase | 5.57 | 0.15 | 5.13 | 0.00 |
| comp32549_c0 | - | 18.04 | 0.64 | 4.69 | 0.00 |
| comp11309_c0 | ERO1-like protein alpha | 18.99 | 2.78 | 2.71 | 0.00 |
| comp13907_c0 | angiopoietin-like 4 | 197.86 | 33.86 | 2.49 | 0.00 |
| comp7566_c0 | - | 22.27 | 3.21 | 2.71 | 0.00 |
| comp16487_c3 | - | 24.30 | 1.38 | 4.08 | 0.00 |
| comp4741_c0 | - | 22.93 | 2.85 | 2.96 | 0.00 |
| comp19740_c0 | myosin IX | 12.39 | 2.16 | 2.45 | 0.00 |
| comp16985_c0 | 3',5'-cyclic-nucleotide phosphodiesterase | 23.35 | 4.09 | 2.45 | 0.00 |
| comp4127_c0 | myeloid leukemia cell differntiation protein MCL-1 | 224.10 | 40.66 | 2.41 | 0.00 |
| comp9512_c0 | - | 6.65 | 0.07 | 6.38 | 0.00 |
| comp7782_c0 | C-type lectin domain family 4, member E | 1053.81 | 104.41 | 3.27 | 0.00 |
| comp8154_c0 | - | 13.21 | 0.40 | 4.98 | 0.00 |
| comp18641_c1 | NUAK family, SNF1-like kinase | 16.85 | 3.21 | 2.32 | 0.00 |
| comp18105_c0 | - | 11.06 | 1.95 | 2.43 | 0.00 |
| comp19397_c1 | class B basic helix-loop-helix protein 3 | 35.86 | 10.10 | 2.27 | 0.00 |
| comp13760_c0 | - | 24.17 | 4.24 | 2.44 | 0.00 |
| comp19862_c0 | - | 17.76 | 3.65 | 2.28 | 0.00 |
| comp16927_c4 | histone deacetylase 4/5 | 11.88 | 2.23 | 2.36 | 0.00 |
| comp18193_c0 | - | 23.39 | 3.90 | 2.49 | 0.00 |
| comp19377_c0 | elongation factor 2 kinase | 33.78 | 2.70 | 3.64 | 0.00 |
| comp14555_c0 | - | 3.77 | 0.09 | 5.34 | 0.00 |
| comp16927_c1 | histone deacetylase 4/5 | 17.90 | 3.54 | 2.26 | 0.00 |
| comp17857_c0 | coproporphyrinogen III oxidase | 24.17 | 2.82 | 3.53 | 0.00 |
| comp12070_c1 | - | 10.34 | 0.73 | 3.76 | 0.00 |
| comp3022_c0 | - | 0.87 | 5.37 | -2.70 | 0.00 |
| comp12733_c0 | - | 118.49 | 12.99 | 3.15 | 0.00 |
| comp19049_c2 | - | 7.84 | 0.42 | 4.17 | 0.00 |
| comp19699_c6 | - | 19.58 | 0.26 | 6.12 | 0.00 |
| comp5284_c0 | - | 7.64 | 1.12 | 2.70 | 0.00 |
| comp17979_c1 | - | 9.26 | 0.55 | 4.05 | 0.00 |
| comp16979_c0 | - | 5.23 | 1.03 | 2.28 | 0.00 |
| comp15901_c1 | glyceraldehyde-3-phosphate dehydrogenase, spermatogenic | 56.88 | 12.49 | 2.13 | 0.00 |
| comp17178_c0 | - | 9.25 | 1.36 | 2.71 | 0.00 |
| comp14797_c1 | MFS transporter, MCP family, solute carrier family 16 (monocarboxylic acid transporters), member 3 | 2.44 | 0.06 | 5.27 | 0.00 |
| comp17883_c0 | - | 4.79 | 0.58 | 2.98 | 0.00 |
| comp17163_c0 | purine-nucleoside phosphorylase | 6.59 | 1.05 | 2.45 | 0.00 |
| comp16093_c0 | - | 5.44 | 0.19 | 4.95 | 0.00 |
| comp19651_c0 | histone-lysine N-methyltransferase SETD1 | 18.24 | 4.27 | 2.04 | 0.00 |
| comp16342_c1 | shingomyelin synthase | 43.96 | 10.63 | 2.00 | 0.00 |
| comp16342_c0 | - | 69.29 | 15.93 | 2.06 | 0.00 |
| comp82029_c0 | - | 0.75 | 5.20 | -2.85 | 0.00 |
| comp17903_c0 | - | 15.19 | 2.97 | 2.29 | 0.00 |
| comp9521_c0 | - | 1.86 | 0.14 | 3.68 | 0.00 |
| comp8017_c0 | - | 84.39 | 10.32 | 2.96 | 0.00 |
| comp19289_c0 | protein polybromo-1 | 127.63 | 32.26 | 1.94 | 0.00 |
| comp8017_c1 | - | 50.41 | 5.68 | 3.08 | 0.00 |
| comp16561_c0 | heparan sulfate 6-O-sulfotransferase HS6ST1 | 2.10 | 8.48 | -2.04 | 0.00 |
| comp8413_c1 | - | 14.23 | 1.49 | 3.16 | 0.00 |
| comp17029_c1 | - | 27.56 | 6.61 | 1.99 | 0.00 |
| comp19104_c0 | lipoprotein lipase | 9.32 | 33.12 | -1.89 | 0.00 |
| comp17883_c1 | - | 7.59 | 1.00 | 2.87 | 0.00 |
| comp19218_c0 | MFS transporter, SP family, solute carrier family 2 (facilitated glucose transporter), member 1 | 10.15 | 3.29 | 1.99 | 0.00 |
| comp16360_c0 | dual specificity phosphatase | 4.62 | 0.79 | 2.51 | 0.00 |
| comp19041_c0 | - | 6.13 | 1.49 | 1.98 | 0.00 |
| comp8626_c0 | - | 14.53 | 3.03 | 2.21 | 0.00 |
| comp15693_c0 | - | 15.28 | 3.19 | 2.16 | 0.00 |
| comp19144_c0 | 3',5'-cyclic-nucleotide phosphodiesterase | 14.07 | 1.00 | 3.57 | 0.00 |
| comp19911_c9 | WD repeat and SOCS box-containing protein 1 | 78.38 | 7.90 | 3.27 | 0.00 |
| comp11059_c0 | - | 5.03 | 0.46 | 3.46 | 0.00 |
| comp19489_c2 | - | 29.14 | 7.69 | 1.85 | 0.00 |
| comp17629_c0 | cadherin 23 | 3.80 | 0.93 | 1.97 | 0.00 |
| comp16398_c0 | - | 117.92 | 32.40 | 1.80 | 0.00 |
| comp16593_c0 | cAMP response element modulator | 9.82 | 1.64 | 2.54 | 0.00 |
| comp10728_c0 | - | 2.44 | 0.13 | 4.13 | 0.00 |
| comp16027_c3 | krueppel-like factor 3/8/12 | 26.41 | 7.50 | 1.85 | 0.00 |
| comp19996_c2 | - | 15.87 | 2.05 | 2.90 | 0.00 |
| comp17379_c3 | - | 12.06 | 2.63 | 2.14 | 0.00 |
| comp17379_c4 | - | 17.29 | 4.12 | 2.02 | 0.00 |
| comp4641_c0 | - | 14.43 | 0.13 | 6.64 | 0.00 |
| comp16329_c0 | nuclear receptor coactivator 4 | 108.19 | 30.85 | 1.73 | 0.00 |
| comp16927_c2 | - | 15.09 | 3.62 | 2.00 | 0.00 |
| comp19462_c0 | - | 9.19 | 2.21 | 1.98 | 0.00 |
| comp3005_c0 | - | 4.18 | 0.69 | 2.55 | 0.00 |
| comp17030_c0 | krueppel-like factor 10/11 | 47.19 | 153.06 | -1.75 | 0.00 |
| comp12039_c0 | - | 6.86 | 0.53 | 3.66 | 0.00 |
| comp19969_c1 | jumonji domain-containing protein 2 | 17.94 | 4.93 | 1.77 | 0.00 |
| comp452874_c0 | - | 1.83 | 0.02 | 6.45 | 0.00 |
| comp19982_c0 | KRAB domain-containing zinc finger protein | 22.07 | 6.36 | 1.74 | 0.00 |
| comp19211_c0 | hexokinase | 12.72 | 4.76 | 1.77 | 0.00 |
| comp12362_c0 | - | 23.41 | 3.61 | 2.65 | 0.00 |
| comp17244_c0 | - | 144.85 | 41.93 | 1.72 | 0.00 |
| comp16430_c1 | - | 35.13 | 9.72 | 1.81 | 0.00 |
| comp14565_c0 | 6-phosphofructokinase | 1.74 | 0.21 | 3.02 | 0.00 |
| comp14617_c0 | - | 5.62 | 0.07 | 6.30 | 0.00 |
| comp9770_c0 | transcription factor AP-1 | 2.23 | 0.33 | 2.69 | 0.00 |
| comp311260_c0 | - | 2.03 | 0.13 | 3.95 | 0.00 |
| comp19410_c6 | BRCA1-associated RING domain protein 1 | 22.51 | 1.97 | 3.47 | 0.00 |
| comp17379_c0 | - | 4.83 | 0.74 | 2.66 | 0.00 |
| comp13915_c0 | phosphoglycerate mutase | 201.08 | 60.18 | 1.69 | 0.00 |
| comp14450_c0 | - | 9.61 | 31.80 | -1.78 | 0.00 |
| comp214672_c0 | - | 4.06 | 0.40 | 3.29 | 0.00 |
| comp16027_c0 | - | 24.78 | 6.97 | 1.77 | 0.00 |
| comp9343_c0 | - | 5.77 | 1.21 | 2.19 | 0.00 |
| comp11597_c1 | potassium channel, subfamily K, member 5 | 3.08 | 0.38 | 2.99 | 0.00 |
| comp6402_c0 | - | 3.10 | 0.09 | 5.05 | 0.00 |
| comp8016_c0 | - | 89.56 | 6.89 | 3.66 | 0.00 |
| comp19931_c0 | glucuronosyltransferase | 5.73 | 17.07 | -1.70 | 0.00 |
| comp7637_c0 | - | 30.23 | 5.02 | 2.54 | 0.00 |
| comp47219_c0 | - | 9.88 | 2.22 | 2.09 | 0.00 |
| comp19493_c2 | IQ motif and SEC7 domain-containing protein | 7.79 | 1.99 | 1.91 | 0.00 |
| comp37299_c0 | - | 16.31 | 3.58 | 2.13 | 0.00 |
| comp660_c0 | - | 1.99 | 0.09 | 4.51 | 0.00 |
| comp14350_c0 | - | 5.78 | 1.06 | 2.32 | 0.00 |
| comp7960_c0 | NUAK family, SNF1-like kinase | 69.50 | 21.08 | 1.66 | 0.00 |
| comp12317_c0 | diphosphoinositol-polyphosphate diphosphatase | 15.66 | 4.33 | 1.80 | 0.00 |
| comp14459_c0 | - | 11.11 | 3.26 | 1.72 | 0.00 |
| comp18285_c0 | - | 10.24 | 2.94 | 1.75 | 0.00 |
| comp15901_c0 | - | 30.12 | 6.41 | 2.18 | 0.00 |
| comp133091_c0 | - | 5.34 | 0.13 | 5.33 | 0.00 |
| comp17379_c1 | - | 8.43 | 2.03 | 1.97 | 0.00 |
| comp15979_c0 | transcription factor CP2 and related proteins | 2.67 | 8.72 | -1.75 | 0.00 |
| comp7960_c1 | NUAK family, SNF1-like kinase | 49.77 | 15.43 | 1.62 | 0.00 |
| comp9290_c0 | - | 6.50 | 1.51 | 2.04 | 0.00 |
| comp5761_c0 | - | 2.57 | 0.20 | 3.60 | 0.00 |
| comp8354_c0 | - | 8.11 | 2.15 | 1.85 | 0.00 |
| comp18521_c3 | - | 9.07 | 1.79 | 2.40 | 0.00 |
| comp17433_c0 | - | 2.49 | 9.20 | -1.94 | 0.00 |
| comp14701_c0 | - | 949.90 | 300.42 | 1.59 | 0.00 |
| comp8626_c1 | - | 8.84 | 1.62 | 2.40 | 0.00 |
| comp17864_c0 | jumonji domain-containing protein 1 | 13.52 | 4.38 | 1.62 | 0.00 |
| comp12230_c0 | carbonic anhydrase | 34.97 | 10.58 | 1.66 | 0.00 |
| comp13589_c0 | - | 17.64 | 5.21 | 1.69 | 0.00 |
| comp16210_c0 | - | 37.04 | 11.74 | 1.61 | 0.00 |
| comp16430_c0 | - | 40.90 | 12.08 | 1.71 | 0.00 |
| comp16561_c1 | - | 2.75 | 12.43 | -2.25 | 0.00 |
| comp15877_c0 | glycogen(starch) synthase | 15.23 | 4.31 | 1.76 | 0.00 |
| comp19152_c1 | F-box and leucine-rich repeat protein 5 | 24.73 | 7.79 | 1.62 | 0.00 |
| comp16770_c0 | - | 12.85 | 3.69 | 1.73 | 0.00 |
| comp17601_c0 | - | 4.26 | 1.27 | 1.67 | 0.00 |
| comp13136_c0 | zinc finger and BTB domain-containing protein 11 | 4.23 | 1.21 | 1.74 | 0.00 |
| comp12753_c0 | - | 7.35 | 2.18 | 1.72 | 0.00 |
| comp17577_c0 | STE20-like kinase | 14.06 | 4.28 | 1.66 | 0.00 |
| comp20020_c1 | - | 9.71 | 3.08 | 1.60 | 0.00 |
| comp13654_c0 | - | 97.34 | 274.98 | -1.56 | 0.00 |
| comp19627_c2 | - | 9.42 | 2.67 | 1.78 | 0.00 |
| comp11597_c0 | - | 4.25 | 0.52 | 3.01 | 0.00 |
| comp88181_c0 | - | 3.58 | 0.58 | 2.53 | 0.00 |
| comp19024_c3 | - | 3.57 | 0.98 | 1.79 | 0.00 |
| comp15194_c1 | - | 0.77 | 3.28 | -2.18 | 0.00 |
| comp8426_c0 | - | 5.90 | 0.56 | 3.32 | 0.00 |
| comp17381_c0 | - | 48.48 | 10.59 | 2.15 | 0.00 |
| comp14871_c1 | - | 14.12 | 4.03 | 1.74 | 0.00 |
| comp16951_c0 | - | 12.21 | 3.73 | 1.63 | 0.00 |
| comp13062_c0 | - | 1.48 | 5.61 | -1.99 | 0.00 |
| comp17306_c1 | arylsulfatase I/J | 8.63 | 2.52 | 1.67 | 0.00 |
| comp9600_c0 | - | 3.51 | 0.70 | 2.27 | 0.00 |
| comp11858_c0 | - | 2.91 | 0.19 | 3.84 | 0.00 |
| comp69021_c0 | - | 5.11 | 1.05 | 2.22 | 0.00 |
| comp17201_c0 | F-box and leucine-rich repeat protein 20 | 37.10 | 7.26 | 2.31 | 0.00 |
| comp11640_c0 | - | 4.90 | 1.45 | 1.71 | 0.00 |
| comp19493_c1 | - | 5.59 | 1.68 | 1.69 | 0.00 |
| comp10381_c1 | SMAD, mothers against DPP 6/7 | 2.86 | 0.57 | 2.27 | 0.00 |
| comp19996_c3 | - | 35.94 | 4.30 | 3.00 | 0.00 |
| comp11803_c0 | fatty acid desaturase 2 (delta-6 desaturase) | 38.91 | 13.02 | 1.51 | 0.00 |
| comp407955_c0 | peroxisome proliferative activated receptor, gamma, coactivator 1, alpha | 0.36 | 1.72 | -2.29 | 0.00 |
| comp9541_c1 | bone morphogenetic protein 2/4 | 0.53 | 2.13 | -2.06 | 0.00 |
| comp6436_c0 | - | 3.24 | 0.33 | 3.25 | 0.00 |
| comp18715_c0 | RAD54-like protein 2 | 13.14 | 4.00 | 1.55 | 0.00 |
| comp157518_c0 | pseudouridylate synthase | 0.55 | 2.32 | -2.12 | 0.00 |
| comp14380_c0 | NAD+ kinase | 2.36 | 7.23 | -1.68 | 0.00 |
| comp8203_c0 | - | 2.20 | 0.52 | 2.05 | 0.00 |
| comp15169_c0 | - | 18.74 | 5.52 | 1.70 | 0.00 |
| comp8413_c0 | - | 17.13 | 2.35 | 2.76 | 0.00 |
| comp10578_c0 | - | 2.20 | 0.14 | 3.90 | 0.00 |
| comp18226_c0 | insulin receptor substrate | 17.85 | 2.86 | 2.61 | 0.00 |
| comp342282_c0 | - | 2.18 | 0.03 | 6.09 | 0.00 |
| comp7063_c0 | - | 2.49 | 0.13 | 4.18 | 0.00 |
| comp111932_c0 | - | 7.36 | 0.35 | 4.37 | 0.00 |
| comp7984_c0 | - | 2.57 | 0.06 | 5.21 | 0.00 |
| comp19528_c1 | 6-phosphofructokinase | 16.03 | 5.64 | 1.50 | 0.00 |
| comp19400_c0 | polycomb group RING finger protein 1 | 13.31 | 4.49 | 1.52 | 0.00 |
| comp8211_c0 | - | 2.11 | 0.30 | 2.72 | 0.00 |
| comp26289_c0 | - | 27.59 | 8.07 | 1.70 | 0.00 |
| comp10980_c0 | - | 3.65 | 11.94 | -1.77 | 0.00 |
| comp14714_c0 | ephrin-B | 4.29 | 1.26 | 1.71 | 0.00 |
| comp19311_c3 | semaphorin 4 | 21.48 | 5.73 | 1.86 | 0.00 |
| comp3974_c0 | - | 12.88 | 3.87 | 1.68 | 0.00 |
| comp21056_c0 | - | 47.88 | 16.24 | 1.49 | 0.00 |
| comp10728_c2 | - | 1.66 | 0.13 | 3.60 | 0.00 |
| comp19117_c0 | - | 14.79 | 4.52 | 1.66 | 0.00 |
| comp485004_c0 | - | 1.59 | 0.04 | 5.19 | 0.00 |
| comp8040_c0 | - | 9.71 | 1.08 | 3.06 | 0.00 |
| comp6256_c0 | - | 0.33 | 1.94 | -2.62 | 0.00 |
| comp19895_c0 | receptor tyrosine-protein kinase erbB-3 | 14.11 | 36.26 | -1.45 | 0.00 |
| comp19049_c0 | - | 6.53 | 0.30 | 4.37 | 0.00 |
| comp13844_c0 | protein-tyrosine phosphatase | 3.34 | 9.32 | -1.55 | 0.00 |
| comp19192_c6 | - | 18.84 | 9.91 | 1.52 | 0.00 |
| comp14775_c0 | phosphatidylserine decarboxylase | 7.72 | 2.23 | 1.75 | 0.00 |
| comp20118_c0 | T-box protein 6 | 4.61 | 1.59 | 1.48 | 0.00 |
| comp4831_c0 | - | 8.63 | 1.38 | 2.59 | 0.00 |
| comp17029_c0 | - | 15.95 | 4.50 | 1.77 | 0.00 |
| comp19996_c6 | - | 68.86 | 9.04 | 2.87 | 0.00 |
| comp13580_c0 | FMS-like tyrosine kinase 1 | 15.06 | 5.21 | 1.49 | 0.00 |
| comp41956_c0 | FK506-binding protein 9/10 | 6.53 | 2.08 | 1.58 | 0.00 |
| comp9895_c0 | - | 10.54 | 28.62 | -1.49 | 0.00 |
| comp381718_c0 | - | 2.08 | 0.17 | 3.58 | 0.00 |
| comp11734_c0 | tumor necrosis factor receptor superfamily, member 9 | 2.41 | 0.50 | 2.17 | 0.00 |
| comp19192_c8 | - | 7.89 | 1.59 | 2.27 | 0.00 |
| comp19286_c0 | - | 23.14 | 6.67 | 1.75 | 0.00 |
| comp11189_c0 | - | 3.14 | 0.95 | 1.66 | 0.00 |
| comp90958_c0 | - | 6.66 | 0.29 | 4.46 | 0.00 |
| comp19192_c7 | - | 4.55 | 0.76 | 2.55 | 0.00 |
| comp17909_c0 | - | 7.35 | 2.47 | 1.53 | 0.00 |
| comp9541_c0 | bone morphogenetic protein 2/4 | 0.51 | 2.13 | -2.12 | 0.00 |
| comp112939_c0 | - | 1.22 | 3.98 | -1.77 | 0.00 |
| comp8045_c0 | - | 6.80 | 2.23 | 1.55 | 0.00 |
| comp4845_c0 | methyl-CpG-binding domain protein 2 | 10.30 | 2.88 | 1.80 | 0.00 |
| comp335807_c0 | connective tissue growth factor | 1.66 | 0.32 | 2.30 | 0.00 |
| comp9760_c1 | - | 4.09 | 0.53 | 2.88 | 0.00 |
| comp14375_c0 | - | 2.48 | 7.80 | -1.71 | 0.00 |
| comp19996_c1 | - | 31.88 | 4.96 | 2.63 | 0.00 |
| comp17422_c0 | - | 8.86 | 2.35 | 1.84 | 0.00 |
| comp15095_c1 | - | 4.55 | 0.63 | 2.76 | 0.00 |
| comp16979_c1 | - | 3.30 | 0.58 | 2.43 | 0.00 |
| comp15319_c0 | carboxypeptidase M | 2.86 | 0.64 | 2.10 | 0.00 |
| comp21298_c0 | - | 68.40 | 24.38 | 1.42 | 0.00 |
| comp4174_c0 | - | 257.72 | 646.43 | -1.39 | 0.00 |
| comp4432_c0 | - | 11.40 | 1.64 | 2.68 | 0.00 |
| comp302128_c0 | - | 2.09 | 0.16 | 3.61 | 0.00 |
| comp12070_c0 | - | 4.73 | 0.23 | 4.27 | 0.00 |
| comp18045_c0 | tumor necrosis factor receptor superfamily, member 10 | 10.62 | 3.74 | 1.45 | 0.00 |
| comp4733_c0 | - | 11.69 | 3.57 | 1.66 | 0.00 |
| comp20237_c3 | PFTAIRE protein kinase | 32.25 | 8.79 | 1.83 | 0.00 |
| comp283723_c0 | - | 2.31 | 0.25 | 3.08 | 0.00 |
| comp5474_c0 | - | 8.72 | 1.81 | 2.24 | 0.00 |
| comp18550_c0 | histone demethylase JARID1 | 11.88 | 4.34 | 1.39 | 0.00 |
| comp19983_c0 | myosin heavy chain | 14.77 | 4.92 | 1.41 | 0.00 |
| comp5932_c0 | - | 2.22 | 0.13 | 4.03 | 0.00 |
| comp17541_c2 | SCAN domain-containing zinc finger protein | 4.51 | 1.59 | 1.45 | 0.00 |
| comp388027_c0 | - | 1.95 | 0.12 | 3.97 | 0.00 |
| comp16431_c0 | hyaluronoglucosaminidase | 12.05 | 30.18 | -1.38 | 0.00 |
| comp10219_c0 | - | 3.42 | 0.80 | 2.04 | 0.00 |
| comp18692_c1 | kinesin family member 1/13/14 | 9.03 | 3.09 | 1.44 | 0.00 |
| comp10980_c1 | - | 2.51 | 7.22 | -1.59 | 0.00 |
| comp640_c0 | - | 1.86 | 0.16 | 3.54 | 0.00 |
| comp19152_c0 | - | 13.88 | 4.83 | 1.47 | 0.00 |
| comp6430_c0 | - | 2.29 | 0.28 | 2.99 | 0.00 |
| comp17365_c0 | - | 72.63 | 9.18 | 2.94 | 0.00 |
| comp19098_c1 | - | 7.70 | 3.13 | 1.49 | 0.00 |
| comp7932_c0 | - | 40.85 | 15.02 | 1.39 | 0.00 |
| comp7596_c0 | transcription initiation factor TFIID subunit D4 | 17.83 | 6.49 | 1.39 | 0.00 |
| comp18539_c0 | - | 6.86 | 2.44 | 1.49 | 0.00 |
| comp11639_c0 | diacylglycerol kinase | 3.58 | 1.24 | 1.49 | 0.00 |
| comp17631_c0 | - | 114.24 | 43.64 | 1.33 | 0.00 |
| comp13910_c0 | fringe | 11.02 | 27.10 | -1.36 | 0.00 |
| comp18470_c0 | fos-like antigen 2 | 20.15 | 5.33 | 1.87 | 0.00 |
| comp18665_c0 | lipoprotein lipase | 34.99 | 83.39 | -1.31 | 0.00 |
| comp17124_c0 | - | 9.50 | 3.37 | 1.45 | 0.00 |
| comp7130_c0 | N-acetylglucosaminyltransferase 3, mucin type | 4.40 | 1.50 | 1.48 | 0.00 |
| comp17287_c2 | - | 37.72 | 8.82 | 2.06 | 0.00 |
| comp13090_c0 | - | 5.06 | 1.01 | 2.32 | 0.00 |
| comp18153_c0 | diacylglycerol kinase | 10.56 | 3.36 | 1.39 | 0.00 |
| comp16984_c0 | nuclear receptor, subfamily 1, group D, member 1 | 1.44 | 7.79 | -2.50 | 0.00 |
| comp10807_c0 | - | 7.28 | 2.11 | 1.71 | 0.00 |
| comp11983_c0 | glucose-6-phosphatase | 525.58 | 1239.73 | -1.28 | 0.00 |
| comp7871_c0 | polycomb protein EED | 16.09 | 4.98 | 1.64 | 0.00 |
| comp16225_c0 | phospholipase C, beta | 1.20 | 3.29 | -1.54 | 0.00 |
| comp12046_c0 | - | 202.47 | 39.10 | 2.33 | 0.00 |
| comp13255_c0 | collagen, type I/II/III/V/XI, alpha | 2.75 | 0.82 | 1.71 | 0.00 |
| comp7719_c0 | - | 1.87 | 0.23 | 3.01 | 0.00 |
| comp12992_c1 | - | 0.76 | 3.24 | -2.14 | 0.00 |
| comp40830_c0 | - | 2.91 | 8.64 | -1.61 | 0.00 |
| comp15401_c0 | - | 138.37 | 2.05 | 6.03 | 0.00 |
| comp18521_c1 | - | 8.05 | 1.47 | 2.39 | 0.00 |
| comp15877_c1 | glycogen(starch) synthase | 15.20 | 5.52 | 1.35 | 0.00 |
| comp7337_c0 | - | 8.39 | 2.46 | 1.69 | 0.00 |
| comp586074_c0 | - | 1.97 | 0.00 | inf | 0.00 |
| comp18867_c0 | - | 5.77 | 0.31 | 4.15 | 0.00 |
| comp14797_c2 | MFS transporter, MCP family, solute carrier family 16 (monocarboxylic acid transporters), member 3 | 1.69 | 0.00 | inf | 0.00 |
| comp5619_c0 | - | 3.76 | 0.08 | 5.43 | 0.00 |
| comp16334_c0 | fructose-2,6-bisphosphatase | 2.84 | 0.88 | 1.61 | 0.00 |
| comp7962_c0 | cytochrome-b5 reductase | 67.57 | 23.74 | 1.46 | 0.00 |
| comp7645_c0 | mannose-6-phosphate isomerase | 22.99 | 8.82 | 1.34 | 0.00 |
| comp7988_c0 | - | 22.62 | 4.75 | 2.21 | 0.00 |
| comp7291_c0 | - | 9.79 | 1.24 | 2.97 | 0.00 |
| comp10912_c0 | - | 7.73 | 0.85 | 3.13 | 0.00 |
| comp15086_c1 | phospholipase C, beta | 0.85 | 2.76 | -1.74 | 0.00 |
| comp18265_c1 | monocyte to macrophage differentiation protein | 27.43 | 11.21 | 1.30 | 0.00 |
| comp8906_c0 | - | 3.74 | 0.98 | 1.89 | 0.00 |
| comp4539_c0 | - | 7.43 | 2.02 | 1.79 | 0.00 |
| comp428_c0 | - | 1.82 | 0.23 | 2.93 | 0.00 |
| comp19656_c0 | solute carrier family 25 (mitochondrial carrier; adenine nucleotide translocator) | 4.47 | 1.45 | 1.58 | 0.00 |
| comp16947_c0 | - | 12.15 | 3.40 | 1.78 | 0.00 |
| comp11782_c0 | hedgehog | 1.63 | 4.19 | -1.44 | 0.00 |
| comp12596_c0 | - | 1.76 | 0.24 | 2.80 | 0.00 |
| comp5298_c0 | - | 2.17 | 0.31 | 2.74 | 0.00 |
| comp8267_c0 | tRNA pseudouridine synthase A | 2.18 | 6.05 | -1.51 | 0.00 |
| comp12125_c0 | regulatory factor X 1/2/3 | 2.36 | 0.68 | 1.73 | 0.00 |
| comp20230_c2 | guanine nucleotide binding protein (G protein), alpha o polypeptide | 45.43 | 20.13 | 1.27 | 0.00 |
| comp192759_c0 | transcription factor AP-4 | 0.66 | 2.17 | -1.78 | 0.00 |
| comp17865_c0 | carbonyl reductase 4 | 1.05 | 3.22 | -1.69 | 0.00 |
| comp216931_c0 | - | 2.48 | 0.47 | 2.35 | 0.00 |
| comp13086_c0 | - | 5.09 | 0.13 | 5.19 | 0.00 |
| comp14198_c1 | - | 37.69 | 13.38 | 1.44 | 0.00 |
| comp18127_c1 | gap junction protein, alpha 3 | 118.55 | 49.82 | 1.25 | 0.00 |
| comp19915_c2 | - | 4.99 | 1.82 | 2.21 | 0.00 |
| comp8673_c0 | - | 10.91 | 3.82 | 1.46 | 0.00 |
| comp16131_c0 | von Hippel-Lindau disease tumor supressor | 10.38 | 3.81 | 1.37 | 0.00 |
| comp18240_c1 | - | 4.39 | 1.45 | 1.54 | 0.00 |
| comp13650_c0 | - | 7.76 | 1.28 | 2.57 | 0.00 |
| comp18036_c0 | protein kinase N | 14.23 | 5.66 | 1.27 | 0.00 |
| comp7280_c0 | - | 2.65 | 0.33 | 2.97 | 0.00 |
| comp9951_c0 | - | 3.59 | 0.37 | 2.99 | 0.00 |
| comp15330_c2 | - | 6.73 | 2.42 | 1.42 | 0.00 |
| comp429816_c0 | - | 1.64 | 0.00 | inf | 0.00 |
| comp9136_c0 | - | 2.71 | 0.15 | 4.13 | 0.00 |
| comp19602_c3 | - | 2.52 | 0.27 | 3.14 | 0.00 |
| comp14450_c1 | - | 4.25 | 12.58 | -1.62 | 0.00 |
| comp15051_c0 | - | 1.27 | 3.39 | -1.47 | 0.00 |
| comp5224_c0 | - | 0.45 | 2.18 | -2.35 | 0.00 |
| comp5662_c0 | - | 1.76 | 0.50 | 1.77 | 0.00 |
| comp16544_c0 | - | 4.26 | 1.33 | 1.63 | 0.00 |
| comp11888_c0 | protein phosphatase 3, regulatory subunit | 39.54 | 12.29 | 1.64 | 0.00 |
| comp13339_c0 | AMP deaminase | 2.84 | 6.77 | -1.31 | 0.00 |
| comp19089_c7 | - | 4.24 | 0.63 | 2.72 | 0.00 |
| comp265223_c0 | - | 2.62 | 0.22 | 3.54 | 0.00 |
| comp19481_c0 | - | 0.61 | 3.05 | -2.36 | 0.00 |
| comp7665_c0 | - | 21.61 | 2.34 | 3.14 | 0.00 |
| comp15503_c0 | protein phosphatase 1, regulatory (inhibitor) subunit 3 | 3.69 | 1.27 | 1.47 | 0.00 |
| comp11719_c1 | - | 1.62 | 0.40 | 1.95 | 0.00 |
| comp15081_c1 | 2-oxoglutarate dehydrogenase E1 component | 5.39 | 1.05 | 2.33 | 0.00 |
| comp68079_c0 | - | 6.32 | 1.94 | 1.63 | 0.00 |
| comp14912_c1 | - | 1.21 | 3.00 | -1.38 | 0.00 |
| comp17306_c0 | Goodpasture-antigen-binding protein kinase | 7.40 | 2.88 | 1.30 | 0.00 |
| comp193526_c0 | - | 3.62 | 0.34 | 3.32 | 0.00 |
| comp17379_c2 | - | 7.80 | 1.92 | 1.96 | 0.00 |
| comp11635_c0 | - | 3.00 | 7.61 | -1.42 | 0.00 |
| comp5617_c0 | - | 2.07 | 0.07 | 4.86 | 0.00 |
| comp12214_c0 | partitioning defective protein 6 | 7.13 | 2.76 | 1.31 | 0.00 |
| comp351724_c0 | kinesin family member 18/19 | 2.47 | 0.02 | 6.58 | 0.00 |
| comp16454_c0 | - | 8.71 | 2.95 | 1.59 | 0.00 |
| comp605413_c0 | - | 1.46 | 0.00 | inf | 0.00 |
| comp18334_c2 | - | 9.56 | 1.70 | 2.48 | 0.00 |
| comp3197_c0 | - | 4.85 | 1.17 | 1.99 | 0.00 |
| comp17125_c0 | - | 5.07 | 1.88 | 1.37 | 0.00 |
| comp16273_c0 | - | 6.35 | 1.27 | 2.20 | 0.00 |
| comp13255_c1 | bromodomain-containing protein 4 | 3.48 | 1.31 | 1.35 | 0.00 |
| comp20010_c0 | - | 13.63 | 5.69 | 1.23 | 0.00 |
| comp5253_c0 | - | 0.75 | 2.00 | -1.48 | 0.00 |
| comp18460_c0 | transglutaminase 2 | 6.68 | 15.25 | -1.27 | 0.00 |
| comp8306_c0 | kinetochore-associated protein 1 | 2.52 | 5.77 | -1.25 | 0.00 |
| comp12806_c0 | - | 2.66 | 0.08 | 5.08 | 0.00 |
| comp15463_c0 | - | 22.17 | 49.63 | -1.23 | 0.00 |
| comp15526_c0 | - | 1.34 | 3.93 | -1.60 | 0.00 |
| comp188523_c0 | tripartite motif-containing protein 35 | 0.85 | 2.51 | -1.62 | 0.00 |
| comp19327_c0 | - | 21.60 | 8.83 | 1.25 | 0.00 |
| comp18521_c2 | - | 9.49 | 1.54 | 2.58 | 0.00 |
| comp44825_c0 | histone H2B | 4.16 | 10.39 | -1.38 | 0.00 |
| comp7896_c0 | RIO kinase 3 | 41.12 | 12.11 | 1.71 | 0.00 |
| comp19089_c0 | Arf-GAP with SH3 domain, ANK repeat and PH domain-containing protein | 8.54 | 0.98 | 3.10 | 0.00 |
| comp7873_c0 | - | 45.57 | 16.28 | 1.44 | 0.00 |
| comp41830_c0 | - | 9.65 | 1.67 | 2.50 | 0.00 |
| comp17574_c0 | - | 1.43 | 3.80 | -1.49 | 0.00 |
| comp19485_c0 | - | 40.41 | 16.96 | 1.20 | 0.00 |
| comp243791_c0 | - | 2.65 | 0.58 | 2.12 | 0.00 |
| comp18721_c0 | alcohol sulfotransferase | 13.03 | 33.05 | -1.40 | 0.00 |
| comp7871_c1 | - | 15.67 | 5.35 | 1.50 | 0.00 |
| comp18220_c0 | PR domain zinc finger protein 2 | 4.16 | 8.38 | -1.24 | 0.00 |
| comp79820_c0 | - | 7.41 | 0.55 | 3.72 | 0.00 |
| comp12039_c1 | - | 7.45 | 0.89 | 3.05 | 0.00 |
| comp17201_c3 | - | 28.76 | 8.24 | 1.76 | 0.00 |
| comp572202_c0 | - | 1.21 | 0.13 | 3.18 | 0.00 |
| comp3353_c0 | - | 2.47 | 0.60 | 1.96 | 0.00 |
| comp13954_c0 | apelin receptor | 6.13 | 2.38 | 1.31 | 0.00 |
| comp9230_c0 | - | 3.95 | 1.15 | 1.74 | 0.00 |
| comp9072_c0 | - | 0.78 | 3.36 | -2.17 | 0.00 |
| comp9313_c0 | - | 4.21 | 0.72 | 2.43 | 0.00 |
| comp16933_c0 | - | 298.50 | 127.32 | 1.18 | 0.00 |
| comp4005_c0 | - | 14.35 | 4.46 | 1.65 | 0.00 |
| comp3989_c0 | - | 18.89 | 6.73 | 1.41 | 0.00 |
| comp18900_c1 | coagulation factor X | 5.03 | 2.00 | 1.35 | 0.00 |
| comp12065_c0 | - | 1.74 | 0.26 | 2.71 | 0.00 |
| comp6218_c0 | - | 9.70 | 3.37 | 1.46 | 0.00 |
| comp12539_c1 | - | 0.57 | 1.68 | -1.60 | 0.00 |
| comp366872_c0 | peroxisome proliferative activated receptor, gamma, coactivator 1, alpha | 1.88 | 0.34 | 2.39 | 0.00 |
| comp15464_c0 | - | 11.31 | 4.61 | 1.25 | 0.00 |
| comp18098_c0 | aconitate hydratase 1 | 9.55 | 3.98 | 1.21 | 0.00 |
| comp19492_c8 | - | 1.48 | 0.02 | 6.38 | 0.00 |
| comp8485_c0 | - | 17.15 | 6.29 | 1.39 | 0.00 |
| comp662679_c0 | - | 1.54 | 0.06 | 4.63 | 0.00 |
| comp11721_c0 | gap junction protein, alpha 4 | 48.40 | 20.57 | 1.17 | 0.00 |
| comp17057_c0 | - | 2.17 | 4.87 | -1.27 | 0.00 |
| comp15957_c0 | cryptochrome | 14.03 | 5.03 | 1.44 | 0.00 |
| comp8659_c0 | - | 1.94 | 0.19 | 3.28 | 0.00 |
| comp7225_c0 | - | 10.89 | 1.52 | 2.77 | 0.00 |
| comp17929_c1 | G protein-coupled receptor 39 | 1.46 | 3.52 | -1.33 | 0.00 |
| comp17636_c0 | long-chain acyl-CoA synthetase | 25.31 | 5.38 | 2.12 | 0.00 |
| comp10699_c2 | - | 4.81 | 1.76 | 1.38 | 0.00 |
| comp19627_c8 | - | 4.77 | 1.03 | 2.19 | 0.00 |
| comp9465_c0 | - | 1.65 | 4.99 | -1.65 | 0.00 |
| comp9702_c0 | - | 2.71 | 0.82 | 1.56 | 0.00 |
| comp8437_c0 | - | 7.03 | 15.96 | -1.24 | 0.00 |
| comp17908_c0 | neurotransmitter:Na+ symporter, NSS family | 5.77 | 2.35 | 1.20 | 0.00 |
| comp18432_c0 | Ras homolog gene family, member U | 5.77 | 14.25 | -1.38 | 0.00 |
| comp21132_c0 | - | 62.85 | 23.91 | 1.34 | 0.00 |
| comp19468_c0 | - | 15.49 | 32.84 | -1.16 | 0.00 |
| comp11126_c1 | - | 1.57 | 0.26 | 2.58 | 0.00 |
| comp19968_c0 | sialidase-2/3/4 | 9.76 | 3.78 | 1.26 | 0.00 |
| comp14189_c0 | - | 1.97 | 5.09 | -1.44 | 0.00 |
| comp18441_c0 | ankyrin | 2.43 | 0.94 | 1.32 | 0.00 |
| comp48699_c0 | - | 1.68 | 0.07 | 4.69 | 0.00 |
| comp2202_c0 | - | 1.67 | 0.17 | 3.22 | 0.00 |
| comp15311_c0 | TNF receptor-associated factor 4 | 4.38 | 1.72 | 1.28 | 0.00 |
| comp14523_c0 | DnaJ homolog, subfamily B, member 2 | 9.64 | 4.49 | 1.23 | 0.00 |
| comp9760_c0 | - | 5.95 | 0.87 | 2.73 | 0.00 |
| comp16072_c0 | - | 6.97 | 2.62 | 1.34 | 0.00 |
| comp530705_c0 | - | 1.75 | 0.00 | inf | 0.00 |
| comp18347_c0 | - | 9.30 | 3.64 | 1.28 | 0.00 |
| comp49191_c0 | fatty acid-binding protein 7, brain | 3.48 | 8.90 | -1.43 | 0.00 |
| comp13969_c0 | - | 2.22 | 4.83 | -1.19 | 0.00 |
| comp361075_c0 | - | 1.89 | 0.41 | 2.17 | 0.00 |
| comp20139_c1 | - | 4.28 | 1.40 | 1.55 | 0.00 |
| comp17636_c1 | - | 8.43 | 2.12 | 1.93 | 0.00 |
| comp20014_c0 | - | 71.43 | 28.26 | 1.25 | 0.00 |
| comp4842_c0 | thyroxine 5-deiodinase | 37.48 | 8.43 | 2.11 | 0.00 |
| comp10219_c1 | jun dimerization protein 2 | 2.71 | 0.60 | 2.11 | 0.00 |
| comp12482_c0 | apoptosis regulator BCL-2 | 2.58 | 0.87 | 1.53 | 0.00 |
| comp12673_c0 | - | 2.49 | 0.90 | 1.42 | 0.00 |
| comp13870_c0 | - | 16.50 | 3.90 | 2.02 | 0.00 |
| comp12112_c0 | - | 7.21 | 2.90 | 1.25 | 0.00 |
| comp7721_c0 | angiopoietin-like 4 | 153.69 | 67.48 | 1.12 | 0.00 |
| comp13773_c2 | - | 3.89 | 1.25 | 1.56 | 0.00 |
| comp10901_c0 | - | 6.21 | 1.55 | 1.97 | 0.00 |
| comp18458_c0 | - | 1.32 | 3.18 | -1.35 | 0.00 |
| comp128720_c0 | - | 2.54 | 0.63 | 1.96 | 0.00 |
| comp14559_c1 | - | 4.69 | 1.08 | 2.03 | 0.00 |
| comp14388_c0 | - | 38.91 | 81.74 | -1.14 | 0.00 |
| comp19541_c0 | - | 10.54 | 0.90 | 3.45 | 0.00 |
| comp5648_c0 | - | 1.86 | 0.48 | 1.87 | 0.00 |
| comp13393_c0 | histone H1/5 | 6.96 | 15.34 | -1.22 | 0.00 |
| comp7065_c0 | - | 11.25 | 2.27 | 2.27 | 0.00 |
| comp15113_c0 | E3 ubiquitin ligase SMURF1/2 | 8.75 | 3.69 | 1.18 | 0.00 |
| comp5054_c0 | DNA-directed RNA polymerase III subunit C2 | 0.76 | 1.98 | -1.44 | 0.00 |
| comp18675_c0 | - | 6.06 | 2.52 | 1.48 | 0.00 |
| comp19918_c0 | - | 5.73 | 14.13 | -1.36 | 0.00 |
| comp15220_c0 | - | 3.93 | 0.06 | 6.16 | 0.00 |
| comp13043_c2 | dual-specificity tyrosine-(Y)-phosphorylation regulated kinase | 1.05 | 3.11 | -1.67 | 0.00 |
| comp19753_c1 | 5'-AMP-activated protein kinase, regulatory gamma subunit | 29.52 | 12.94 | 1.14 | 0.00 |
| comp310330_c0 | - | 2.69 | 0.11 | 4.57 | 0.00 |
| comp13743_c1 | cyclin-dependent kinase inhibitor 1B | 24.65 | 10.40 | 1.14 | 0.00 |
| comp8602_c1 | - | 5.84 | 1.19 | 2.24 | 0.00 |
| comp669311_c0 | - | 1.49 | 0.19 | 2.98 | 0.00 |
| comp17422_c1 | - | 11.58 | 4.08 | 1.45 | 0.00 |
| comp118910_c0 | - | 1.09 | 2.67 | -1.34 | 0.00 |
| comp9757_c0 | - | 3.10 | 0.39 | 2.80 | 0.00 |
| comp16358_c0 | actinin alpha | 2.61 | 5.67 | -1.17 | 0.00 |
| comp17287_c1 | - | 41.95 | 10.96 | 1.90 | 0.00 |
| comp8271_c0 | - | 9.07 | 0.92 | 3.28 | 0.00 |
| comp19639_c0 | - | 13.41 | 4.93 | 1.33 | 0.00 |
| comp6886_c0 | ATP-dependent DNA helicase 2 subunit 1 | 2.30 | 5.14 | -1.23 | 0.00 |
| comp11777_c0 | protein kinase A | 1.18 | 2.88 | -1.35 | 0.00 |
| comp13726_c2 | molecular chaperone HtpG | 28.35 | 11.06 | 1.32 | 0.00 |
| comp19295_c0 | dual specificity phosphatase | 20.65 | 9.16 | 1.12 | 0.00 |
| comp15330_c0 | - | 8.65 | 3.60 | 1.20 | 0.00 |
| comp18976_c0 | - | 7.68 | 1.69 | 2.25 | 0.00 |
| comp348028_c0 | - | 2.32 | 0.19 | 3.54 | 0.00 |
| comp14871_c0 | - | 5.09 | 0.64 | 2.90 | 0.00 |
| comp63150_c0 | - | 7.75 | 2.08 | 1.84 | 0.00 |
| comp3769_c0 | GTP cyclohydrolase I | 0.76 | 3.13 | -2.10 | 0.00 |
| comp8707_c0 | - | 11.11 | 3.97 | 1.40 | 0.00 |
| comp4907_c0 | - | 2.22 | 0.18 | 3.51 | 0.00 |
| comp18856_c0 | - | 18.64 | 8.07 | 1.13 | 0.00 |
| comp14932_c1 | long-chain acyl-CoA synthetase | 1.85 | 0.64 | 1.45 | 0.00 |
| comp7480_c0 | aldehyde reductase | 13.63 | 28.26 | -1.11 | 0.00 |
| comp14143_c0 | - | 1.18 | 3.12 | -1.45 | 0.00 |
| comp8280_c0 | - | 6.72 | 2.34 | 1.46 | 0.00 |
| comp14115_c0 | acetylserotonin N-methyltransferase | 49.60 | 100.34 | -1.09 | 0.00 |
| comp6237_c0 | huntingtin-associated protein 1 | 1.99 | 0.19 | 3.33 | 0.00 |
| comp18342_c2 | mannosyl-oligosaccharide alpha-1,2-mannosidase | 1.38 | 3.16 | -1.26 | 0.00 |
| comp12233_c0 | myosin IX | 20.77 | 9.24 | 1.10 | 0.00 |
| comp16219_c0 | histone demethylase JMJD3 | 5.23 | 1.22 | 2.06 | 0.00 |
| comp504789_c0 | - | 1.86 | 0.21 | 3.12 | 0.00 |
| comp15963_c0 | mitogen-activated protein kinase kinase kinase | 1.35 | 2.90 | -1.16 | 0.00 |
| comp19378_c0 | - | 94.71 | 38.99 | 1.27 | 0.00 |
| comp3593_c1 | - | 2.57 | 0.36 | 2.74 | 0.00 |
| comp14606_c1 | relaxin family peptide receptor 3 | 2.86 | 1.05 | 1.42 | 0.00 |
| comp17028_c0 | - | 1.12 | 2.61 | -1.28 | 0.00 |
| comp14901_c0 | LIM domain kinase 2 | 6.87 | 3.01 | 1.14 | 0.01 |
| comp438333_c0 | - | 0.22 | 1.55 | -2.85 | 0.01 |
| comp2873_c0 | - | 3.82 | 1.23 | 1.56 | 0.01 |
| comp18772_c0 | - | 7.62 | 2.88 | 1.34 | 0.01 |
| comp20056_c0 | inorganic phosphate transporter, PiT family | 39.54 | 18.11 | 1.06 | 0.01 |
| comp7840_c0 | - | 1.84 | 0.36 | 2.32 | 0.01 |
| comp3578_c0 | - | 3.44 | 0.47 | 2.75 | 0.01 |
| comp14288_c0 | - | 7.93 | 2.67 | 1.54 | 0.01 |
| comp8799_c1 | nuclear receptor, subfamily 2, group F, member 2 | 0.90 | 2.68 | -1.64 | 0.01 |
| comp11294_c0 | layilin | 1.16 | 2.74 | -1.31 | 0.01 |
| comp16810_c0 | - | 11.52 | 4.91 | 1.17 | 0.01 |
| comp17281_c0 | death-associated protein kinase | 2.60 | 6.72 | -1.45 | 0.01 |
| comp3786_c0 | chemokine binding protein 2 | 2.73 | 0.97 | 1.45 | 0.01 |
| comp4507_c0 | - | 26.76 | 6.55 | 1.96 | 0.01 |
| comp347896_c0 | - | 2.40 | 0.11 | 4.31 | 0.01 |
| comp470758_c0 | - | 1.74 | 0.21 | 3.00 | 0.01 |
| comp19840_c0 | transferrin receptor | 122.11 | 43.55 | 1.44 | 0.01 |
| comp20162_c0 | E3 ubiquitin-protein ligase HERC1 | 21.64 | 8.03 | 1.38 | 0.01 |
| comp21518_c0 | - | 64.75 | 8.34 | 2.92 | 0.01 |
| comp7338_c0 | - | 3.02 | 6.76 | -1.23 | 0.01 |
| comp9585_c0 | - | 4.50 | 1.67 | 1.37 | 0.01 |
| comp18612_c0 | ferredoxin hydrogenase | 8.19 | 17.28 | -1.09 | 0.01 |
| comp5776_c0 | - | 2.41 | 0.25 | 3.21 | 0.01 |
| comp15028_c0 | - | 12.29 | 5.28 | 1.16 | 0.01 |
| comp167783_c0 | - | 1.86 | 0.58 | 1.64 | 0.01 |
| comp13836_c2 | protein-serine/threonine kinase | 9.99 | 1.56 | 2.73 | 0.01 |
| comp15095_c0 | - | 6.64 | 1.31 | 2.25 | 0.01 |
| comp19946_c2 | - | 14.04 | 1.84 | 2.90 | 0.01 |
| comp11600_c1 | - | 13.98 | 2.14 | 2.69 | 0.01 |
| comp660672_c0 | - | 1.52 | 0.00 | inf | 0.01 |
| comp100683_c0 | - | 1.29 | 4.22 | -1.75 | 0.01 |
| comp13836_c3 | - | 5.15 | 0.72 | 2.82 | 0.01 |
| comp116741_c0 | - | 6.01 | 0.16 | 5.25 | 0.01 |
| comp15644_c0 | triple functional domain protein | 2.21 | 4.60 | -1.13 | 0.01 |
| comp19646_c0 | - | 12.30 | 5.17 | 1.19 | 0.01 |
| comp15194_c0 | - | 0.79 | 2.65 | -1.82 | 0.01 |
| comp16238_c0 | - | 4.90 | 0.88 | 2.44 | 0.01 |
| comp10061_c0 | - | 1.23 | 3.12 | -1.42 | 0.01 |
| comp3830_c0 | - | 4.78 | 9.98 | -1.13 | 0.01 |
| comp18566_c0 | groucho | 3.11 | 7.59 | -1.51 | 0.01 |
| comp262436_c0 | arylsulfatase I/J | 2.19 | 0.71 | 1.57 | 0.01 |
| comp5459_c0 | - | 2.36 | 0.29 | 2.93 | 0.01 |
| comp20128_c0 | adenylate cyclase 5 | 15.68 | 6.27 | 1.33 | 0.01 |
| comp5435_c0 | - | 2.06 | 0.42 | 2.22 | 0.01 |
| comp13836_c0 | - | 1.65 | 0.28 | 2.50 | 0.01 |
| comp14077_c0 | InaD-like protein | 6.20 | 2.69 | 1.15 | 0.01 |
| comp17651_c0 | - | 26.37 | 8.11 | 1.65 | 0.01 |
| comp10699_c0 | - | 2.33 | 0.64 | 1.82 | 0.01 |
| comp10453_c0 | - | 3.14 | 1.05 | 1.52 | 0.01 |
| comp7797_c0 | chromatin licensing and DNA replication factor 1 | 0.62 | 1.79 | -1.61 | 0.01 |
| comp14747_c0 | - | 4.01 | 8.55 | -1.15 | 0.01 |
| comp19489_c1 | - | 82.61 | 38.00 | 1.08 | 0.01 |
| comp3662_c0 | - | 1.34 | 3.40 | -1.40 | 0.01 |
| comp8203_c1 | - | 2.18 | 0.46 | 2.21 | 0.01 |
| comp3590_c0 | - | 3.08 | 0.50 | 2.57 | 0.01 |
| comp166179_c0 | - | 2.93 | 0.50 | 2.55 | 0.01 |
| comp559161_c0 | - | 1.75 | 0.06 | 4.81 | 0.01 |
| comp645573_c0 | - | 1.43 | 0.00 | inf | 0.01 |
| comp19552_c0 | Cu2+-exporting ATPase | 20.01 | 4.73 | 2.03 | 0.01 |
| comp8759_c1 | - | 1.17 | 2.74 | -1.28 | 0.01 |
| comp18728_c0 | - | 4.76 | 0.95 | 2.24 | 0.01 |
| comp8219_c0 | - | 3.44 | 8.78 | -1.41 | 0.01 |
| comp4640_c0 | - | 4.17 | 10.57 | -1.39 | 0.01 |
| comp444418_c0 | methionyl aminopeptidase | 1.61 | 0.38 | 2.01 | 0.01 |
| comp176125_c0 | - | 2.86 | 0.12 | 4.56 | 0.01 |
| comp15273_c1 | - | 4.70 | 1.76 | 1.35 | 0.01 |
| comp19695_c0 | - | 48.80 | 16.81 | 1.09 | 0.01 |
| comp7697_c0 | histone H2A | 68.24 | 134.11 | -1.05 | 0.01 |
| comp53680_c0 | tRNA (adenine-N1-)-methyltransferase | 3.04 | 6.65 | -1.18 | 0.01 |
| comp15319_c1 | carboxypeptidase M | 1.67 | 0.50 | 1.68 | 0.01 |
| comp14838_c0 | phosphomannomutase | 34.37 | 15.86 | 1.07 | 0.01 |
| comp15594_c0 | 3',5'-cyclic-nucleotide phosphodiesterase | 3.39 | 7.16 | -1.15 | 0.01 |
| comp3593_c0 | - | 4.18 | 0.87 | 2.20 | 0.01 |
| comp12214_c1 | - | 7.61 | 3.20 | 1.17 | 0.01 |
| comp567141_c0 | - | 0.16 | 1.77 | -3.50 | 0.01 |
| comp15937_c2 | - | 15.80 | 4.04 | 1.93 | 0.01 |
| comp12182_c2 | transforming growth factor, alpha | 3.07 | 0.73 | 2.07 | 0.01 |
| comp149947_c0 | - | 3.96 | 0.40 | 3.32 | 0.01 |
| comp85454_c0 | solute carrier family 1 (neutral amino acid transporter), member 5 | 4.20 | 1.45 | 1.49 | 0.01 |
| comp8298_c0 | zinc finger and BTB domain-containing protein 16 | 8.61 | 3.77 | 1.16 | 0.01 |
| comp17837_c1 | - | 2.15 | 4.67 | -1.20 | 0.01 |
| comp17415_c0 | ethanolamine-phosphate cytidylyltransferase | 10.63 | 5.58 | 1.08 | 0.01 |
| comp5922_c0 | cell cycle checkpoint protein | 0.63 | 2.13 | -1.83 | 0.01 |
| comp10615_c0 | peroxisome proliferative activated receptor, gamma, coactivator 1, alpha | 1.82 | 0.34 | 2.35 | 0.01 |
| comp10953_c0 | 1-phosphatidylinositol-5-phosphate 4-kinase | 4.92 | 2.13 | 1.15 | 0.01 |
| comp13563_c0 | mitochondrial brown fat uncoupling protein 1 | 123.83 | 7.50 | 4.03 | 0.01 |
| comp8539_c0 | DNA polymerase I | 0.52 | 2.21 | -2.14 | 0.01 |
| comp19810_c0 | bile acid-CoA:amino acid N-acyltransferase | 35.31 | 13.17 | 1.12 | 0.01 |
| comp5885_c0 | - | 2.27 | 0.18 | 3.69 | 0.01 |
| comp307943_c0 | SCAN domain-containing zinc finger protein | 0.80 | 1.95 | -1.35 | 0.01 |
| comp14369_c0 | glucosamine--fructose-6-phosphate aminotransferase (isomerizing) | 3.52 | 1.43 | 1.25 | 0.01 |
| comp11504_c0 | - | 10.30 | 3.73 | 1.40 | 0.01 |
| comp225278_c0 | deoxyguanosine kinase | 0.86 | 2.37 | -1.53 | 0.01 |
| comp17214_c0 | RUN and FYVE domain-containing protein 1 | 15.26 | 7.06 | 1.05 | 0.01 |
| comp8255_c2 | - | 1.95 | 0.48 | 1.96 | 0.01 |
| comp12627_c0 | nuclear factor of kappa light polypeptide gene enhancer in B-cells inhibitor, alpha | 51.24 | 22.27 | 1.05 | 0.01 |
| comp17989_c1 | thymidine phosphorylase | 8.08 | 16.24 | -1.05 | 0.01 |
| comp18992_c2 | - | 7.10 | 1.64 | 2.09 | 0.01 |
| comp16016_c1 | - | 4.47 | 9.94 | -1.06 | 0.01 |
| comp13716_c0 | - | 5.22 | 1.12 | 2.15 | 0.01 |
| comp18848_c1 | - | 47.94 | 21.97 | 1.04 | 0.01 |
| comp19575_c0 | Rab11 family-interacting protein 3/4 | 53.90 | 23.91 | 1.03 | 0.01 |
| comp18764_c1 | - | 10.65 | 29.15 | -1.49 | 0.01 |
| comp441440_c0 | - | 0.33 | 1.66 | -2.38 | 0.01 |
| comp19754_c0 | - | 22.92 | 10.60 | 1.03 | 0.01 |
| comp19192_c4 | - | 3.66 | 8.45 | -1.26 | 0.01 |
| comp15526_c1 | - | 0.73 | 2.97 | -2.07 | 0.01 |
| comp142435_c0 | - | 1.14 | 2.74 | -1.33 | 0.01 |
| comp14976_c0 | - | 6.40 | 12.32 | -1.09 | 0.01 |
| comp70822_c0 | - | 9.07 | 0.83 | 3.36 | 0.01 |
| comp519759_c0 | - | 1.37 | 0.17 | 3.00 | 0.01 |
| comp11248_c0 | methylsterol monooxygenase | 13.75 | 4.97 | 1.41 | 0.01 |
| comp15937_c0 | - | 24.40 | 5.58 | 2.09 | 0.01 |
| comp17257_c0 | alcohol dehydrogenase (NADP+) | 6.97 | 13.89 | -1.07 | 0.01 |
| comp660701_c0 | - | 1.41 | 0.25 | 2.43 | 0.01 |
| comp15330_c1 | - | 7.77 | 3.36 | 1.15 | 0.01 |
| comp133232_c0 | heart-and neural crest derivatives-expressed protein | 0.98 | 2.53 | -1.44 | 0.01 |
| comp15700_c0 | - | 1.99 | 4.40 | -1.22 | 0.01 |
| comp16573_c0 | - | 9.55 | 4.19 | 1.13 | 0.01 |
| comp17890_c0 | - | 3.73 | 8.24 | -1.08 | 0.01 |
| comp18500_c2 | coagulation factor VII | 1.29 | 2.71 | -1.14 | 0.01 |
| comp14810_c0 | - | 68.54 | 6.65 | 3.26 | 0.01 |
| comp15060_c0 | - | 2.66 | 5.44 | -1.10 | 0.01 |
| comp607112_c0 | - | 1.48 | 0.13 | 3.40 | 0.01 |
| comp3822_c0 | - | 20.44 | 3.85 | 2.33 | 0.01 |
| comp6718_c0 | - | 1.66 | 0.11 | 3.93 | 0.01 |
| comp268336_c0 | - | 0.60 | 2.47 | -2.13 | 0.01 |
| comp6598_c0 | - | 1.87 | 0.20 | 3.14 | 0.01 |
| comp3468_c0 | - | 1.06 | 2.57 | -1.33 | 0.01 |
| comp2836_c0 | - | 1.52 | 4.14 | -1.51 | 0.01 |
| comp127641_c0 | - | 2.70 | 0.13 | 4.33 | 0.01 |
| comp18308_c1 | - | 10.16 | 3.05 | 1.67 | 0.01 |
| comp16758_c0 | bromodomain-containing factor 1 | 3.87 | 1.77 | 1.07 | 0.01 |
| comp18632_c0 | - | 34.60 | 8.46 | 2.01 | 0.01 |
| comp5367_c0 | - | 2.17 | 0.29 | 2.86 | 0.01 |
| comp10482_c1 | - | 2.43 | 6.14 | -1.39 | 0.01 |
| comp327530_c0 | - | 1.84 | 0.40 | 2.15 | 0.01 |
| comp9323_c0 | - | 5.34 | 0.16 | 5.02 | 0.01 |
| comp10213_c0 | - | 2.18 | 0.23 | 3.20 | 0.01 |
| comp4766_c0 | - | 1.62 | 3.37 | -1.13 | 0.01 |
| comp10138_c0 | - | 3.96 | 0.76 | 2.30 | 0.01 |
| comp7622_c0 | toll-like recepto 5 | 17.61 | 7.10 | 1.27 | 0.01 |
| comp632306_c0 | topoisomerase (DNA) II binding protein 1 | 0.20 | 1.43 | -2.91 | 0.01 |
| comp189571_c0 | - | 2.65 | 0.28 | 3.19 | 0.01 |
| comp18276_c0 | - | 32.92 | 11.76 | 1.44 | 0.01 |
| comp14668_c1 | - | 11.71 | 4.49 | 1.34 | 0.01 |
| comp8455_c0 | - | 15.88 | 7.21 | 1.08 | 0.01 |
| comp769027_c0 | - | 1.20 | 0.14 | 3.07 | 0.01 |
| comp14444_c2 | - | 4.05 | 1.04 | 1.93 | 0.01 |
| comp19995_c2 | - | 26.99 | 9.85 | 1.52 | 0.01 |
| comp14995_c0 | nuclear receptor, subfamily 2, group F, member 6 | 6.79 | 14.44 | -1.16 | 0.01 |
| comp14992_c0 | - | 4.52 | 1.80 | 1.29 | 0.01 |
| comp14919_c0 | - | 0.96 | 2.45 | -1.40 | 0.01 |
| comp449386_c0 | nucleoside-triphosphatase | 1.69 | 0.46 | 1.81 | 0.01 |
| comp11461_c0 | proline racemase | 0.74 | 2.04 | -1.55 | 0.01 |
| comp15480_c0 | - | 7.42 | 1.47 | 2.30 | 0.01 |
| comp180103_c0 | G protein-coupled receptor 161 | 0.80 | 2.38 | -1.61 | 0.01 |
| comp8754_c0 | - | 1.18 | 3.02 | -1.43 | 0.01 |
| comp17531_c0 | myosin I | 3.35 | 6.53 | -1.03 | 0.01 |
| comp11265_c0 | - | 19.05 | 0.48 | 5.15 | 0.01 |
| comp20614_c0 | - | 117.62 | 56.88 | 0.99 | 0.01 |
| comp3755_c0 | - | 4.09 | 0.75 | 2.39 | 0.01 |
| comp3405_c0 | replication factor A2 | 1.22 | 2.93 | -1.33 | 0.01 |
| comp19610_c1 | - | 11.32 | 5.34 | 1.05 | 0.01 |
| comp15725_c0 | platelet derived growth factor A/B | 13.02 | 5.87 | 1.06 | 0.01 |
| comp20092_c1 | - | 19.57 | 38.17 | -0.99 | 0.01 |
| comp7326_c0 | MAP kinase interacting serine/threonine kinase | 2.35 | 4.83 | -1.11 | 0.01 |
| comp16225_c2 | - | 0.14 | 1.68 | -3.59 | 0.01 |
| comp19036_c1 | - | 4.64 | 1.92 | 1.22 | 0.01 |
| comp12927_c0 | Ras-related protein Rab-20 | 2.16 | 0.69 | 1.60 | 0.01 |
| comp18044_c0 | - | 8.55 | 20.90 | -1.38 | 0.01 |
| comp16988_c1 | - | 7.02 | 16.15 | -1.28 | 0.01 |
| comp13216_c0 | F-box and leucine-rich repeat protein 2 | 7.39 | 3.43 | 1.05 | 0.01 |
| comp20037_c2 | - | 5.37 | 10.52 | -1.06 | 0.01 |
| comp19036_c2 | - | 450.06 | 229.82 | 0.98 | 0.01 |
| comp145404_c0 | - | 1.16 | 2.95 | -1.38 | 0.01 |
| comp14865_c0 | - | 21.95 | 1.62 | 3.68 | 0.01 |
| comp9439_c0 | tumor necrosis factor, alpha-induced protein 3 | 3.42 | 1.52 | 1.11 | 0.01 |
| comp16386_c0 | - | 3.63 | 7.37 | -1.09 | 0.01 |
| comp13694_c0 | - | 10.81 | 20.81 | -1.01 | 0.01 |
| comp18947_c1 | - | 31.64 | 59.83 | -0.98 | 0.01 |
| comp7282_c0 | - | 1.98 | 0.11 | 4.05 | 0.01 |
| comp1133_c0 | - | 2.04 | 0.34 | 2.51 | 0.01 |
| comp5418_c0 | - | 3.20 | 0.50 | 2.63 | 0.01 |
| comp5710_c0 | - | 0.35 | 1.91 | -2.51 | 0.01 |
| comp12543_c0 | - | 25.47 | 9.29 | 1.37 | 0.02 |
| comp560301_c0 | - | 1.56 | 0.13 | 3.57 | 0.02 |
| comp4890_c0 | - | 2.06 | 0.17 | 3.54 | 0.02 |
| comp17837_c0 | cysteine rich protein 61 | 2.61 | 5.38 | -1.11 | 0.02 |
| comp19255_c0 | - | 6.75 | 14.27 | -1.15 | 0.02 |
| comp13956_c0 | - | 30.83 | 14.82 | 0.98 | 0.02 |
| comp256091_c0 | - | 2.58 | 0.24 | 3.40 | 0.02 |
| comp9590_c1 | PH and SEC7 domain-containing protein | 0.92 | 2.28 | -1.36 | 0.02 |
| comp10183_c0 | - | 4.36 | 0.93 | 2.16 | 0.02 |
| comp636784_c0 | - | 1.13 | 0.12 | 3.14 | 0.02 |
| comp17809_c0 | myeloid/lymphoid or mixed-lineage leukemia protein 5 | 4.07 | 1.87 | 1.05 | 0.02 |
| comp17922_c0 | - | 63.14 | 30.79 | 0.99 | 0.02 |
| comp18091_c2 | - | 31.77 | 15.23 | 0.98 | 0.02 |
| comp8358_c0 | malonyl-CoA decarboxylase | 2.97 | 6.08 | -1.10 | 0.02 |
| comp19394_c0 | transient receptor potential cation channel, subfamily C, member 6 | 7.86 | 3.33 | 1.04 | 0.02 |
| comp4171_c0 | - | 15.46 | 29.87 | -1.02 | 0.02 |
| comp5028_c0 | - | 4.02 | 1.10 | 1.81 | 0.02 |
| comp12648_c0 | - | 1.87 | 3.87 | -1.12 | 0.02 |
| comp14185_c0 | - | 27.28 | 12.77 | 1.02 | 0.02 |
| comp12313_c1 | - | 30.25 | 12.71 | 1.20 | 0.02 |
| comp19628_c0 | acetyl-CoA synthetase | 20.75 | 38.94 | -0.96 | 0.02 |
| comp17310_c0 | - | 25.30 | 12.42 | 0.98 | 0.02 |
| comp9463_c0 | - | 4.12 | 1.43 | 1.46 | 0.02 |
| comp9877_c0 | histamine N-methyltransferase | 1.11 | 2.62 | -1.31 | 0.02 |
| comp17709_c0 | hepatocyte growth factor | 30.97 | 14.92 | 0.98 | 0.02 |
| comp15867_c0 | C-terminal binding protein | 16.71 | 4.79 | 1.69 | 0.02 |
| comp18091_c0 | - | 19.34 | 8.89 | 1.07 | 0.02 |
| comp210735_c0 | protein-serine/threonine kinase | 3.01 | 0.54 | 2.43 | 0.02 |
| comp13773_c0 | transcription factor CP2 and related proteins | 3.50 | 1.35 | 1.49 | 0.02 |
| comp4914_c1 | - | 3.01 | 0.36 | 2.98 | 0.02 |
| comp11841_c0 | [heparan sulfate]-glucosamine 3-sulfotransferase 3 | 16.03 | 29.90 | -0.96 | 0.02 |
| comp10224_c0 | synuclein, alpha interacting protein (synphilin) | 1.11 | 2.38 | -1.15 | 0.02 |
| comp17169_c0 | - | 2.25 | 1.00 | 1.11 | 0.02 |
| comp15311_c1 | - | 2.24 | 0.56 | 1.89 | 0.02 |
| comp19294_c0 | - | 4.70 | 2.14 | 1.08 | 0.02 |
| comp4545_c0 | - | 2.61 | 5.60 | -1.17 | 0.02 |
| comp11123_c0 | 2-hydroxyacylsphingosine 1-beta-galactosyltransferase | 14.80 | 7.50 | 0.99 | 0.02 |
| comp543874_c0 | - | 1.43 | 0.16 | 3.18 | 0.02 |
| comp17541_c1 | - | 5.48 | 2.55 | 1.03 | 0.02 |
| comp9104_c0 | sestrin | 2.60 | 1.03 | 1.30 | 0.02 |
| comp17724_c0 | - | 2.26 | 4.57 | -1.09 | 0.02 |
| comp19090_c0 | - | 61.72 | 21.80 | 1.47 | 0.02 |
| comp19818_c2 | - | 20.24 | 9.42 | 1.04 | 0.02 |
| comp15055_c0 | - | 7.31 | 3.36 | 1.06 | 0.02 |
| comp18065_c2 | - | 2.12 | 0.19 | 3.44 | 0.02 |
| comp7684_c0 | - | 21.68 | 10.41 | 0.99 | 0.02 |
| comp19670_c0 | nicotinamide phosphoribosyltransferase | 24.50 | 12.01 | 0.96 | 0.02 |
| comp9157_c0 | - | 1.45 | 3.63 | -1.39 | 0.02 |
| comp9504_c0 | purinergic receptor P2Y, G protein-coupled, 5 | 1.41 | 2.93 | -1.12 | 0.02 |
| comp16617_c0 | - | 5.02 | 9.93 | -1.04 | 0.02 |
| comp16334_c1 | - | 2.36 | 0.57 | 1.97 | 0.02 |
| comp12836_c1 | - | 4.38 | 1.82 | 1.21 | 0.02 |
| comp206963_c0 | Ras homolog gene family, member T1 | 0.98 | 2.06 | -1.13 | 0.02 |
| comp16321_c0 | - | 2.73 | 5.82 | -1.15 | 0.02 |
| comp287843_c0 | - | 0.73 | 2.06 | -1.58 | 0.02 |
| comp11026_c1 | - | 1.97 | 0.22 | 3.15 | 0.02 |
| comp12027_c0 | - | 1.36 | 0.07 | 4.25 | 0.02 |
| comp4950_c0 | - | 2.10 | 0.74 | 1.46 | 0.02 |
| comp14769_c0 | - | 49.76 | 24.22 | 0.98 | 0.02 |
| comp15475_c0 | glucuronyl/N-acetylglucosaminyl transferase EXT1 | 2.96 | 7.10 | -1.33 | 0.02 |
| comp15964_c0 | - | 8.04 | 3.28 | 1.14 | 0.02 |
| comp505720_c0 | - | 1.46 | 0.29 | 2.28 | 0.02 |
| comp116524_c0 | cyclin-dependent kinase inhibitor 1B | 4.23 | 1.66 | 1.30 | 0.02 |
| comp17449_c0 | sterol regulatory element-binding transcription factor 2 | 37.28 | 18.53 | 0.96 | 0.02 |
| comp7465_c0 | - | 11.68 | 22.00 | -0.98 | 0.02 |
| comp8994_c0 | - | 2.28 | 0.54 | 2.00 | 0.02 |
| comp9798_c0 | - | 1.70 | 0.12 | 3.77 | 0.02 |
| comp16469_c0 | - | 2.00 | 0.45 | 2.07 | 0.02 |
| comp17607_c0 | O-phospho-L-seryl-tRNASec:L-selenocysteinyl-tRNA synthase | 2.60 | 5.21 | -1.07 | 0.02 |
| comp7017_c0 | DNA polymerase lambda subunit | 0.99 | 2.33 | -1.30 | 0.02 |
| comp172621_c0 | - | 4.08 | 0.54 | 2.86 | 0.02 |
| comp9798_c2 | - | 1.76 | 0.23 | 2.83 | 0.02 |
| comp16792_c0 | - | 13.66 | 6.63 | 0.98 | 0.02 |
| comp17626_c0 | - | 6.07 | 14.79 | -1.20 | 0.02 |
| comp181808_c0 | histone-lysine N-methyltransferase SUV420H | 2.77 | 1.08 | 1.31 | 0.02 |
| comp528589_c0 | - | 1.37 | 0.21 | 2.67 | 0.02 |
| comp18118_c2 | - | 4.24 | 8.36 | -1.05 | 0.02 |
| comp14953_c0 | nuclear receptor, subfamily 1, group C, member 2 | 5.99 | 11.14 | -0.96 | 0.02 |
| comp370571_c0 | - | 0.35 | 1.93 | -2.57 | 0.02 |
| comp3209_c0 | - | 4.86 | 1.78 | 1.37 | 0.02 |
| comp311743_c0 | - | 2.49 | 0.38 | 2.65 | 0.02 |
| comp19627_c5 | - | 3.73 | 1.05 | 1.79 | 0.02 |
| comp11271_c0 | 7,8-dihydro-8-oxoguanine triphosphatase | 0.83 | 2.14 | -1.40 | 0.02 |
| comp17314_c0 | - | 15.05 | 7.25 | 0.95 | 0.02 |
| comp17136_c1 | ubiquitin carboxyl-terminal hydrolase 43 | 1.58 | 0.52 | 1.53 | 0.02 |
| comp1657_c0 | - | 1.61 | 0.30 | 2.40 | 0.02 |
| comp15817_c0 | - | 59.21 | 16.79 | 1.79 | 0.02 |
| comp14782_c0 | - | 9.80 | 3.37 | 1.51 | 0.02 |
| comp10359_c1 | - | 2.87 | 0.23 | 3.55 | 0.02 |
| comp17583_c0 | - | 15.50 | 7.56 | 0.97 | 0.02 |
| comp15249_c0 | phosphoenolpyruvate carboxykinase (GTP) | 1314.49 | 273.09 | 2.23 | 0.02 |
| comp9920_c0 | - | 1.29 | 3.41 | -1.46 | 0.02 |
| comp15359_c0 | xylosylprotein 4-beta-galactosyltransferase | 1.83 | 3.76 | -1.10 | 0.02 |
| comp9860_c1 | - | 0.17 | 1.42 | -3.09 | 0.02 |
| comp14013_c0 | - | 1.32 | 2.89 | -1.20 | 0.02 |
| comp17477_c0 | - | 16.43 | 0.47 | 5.09 | 0.02 |
| comp14185_c1 | - | 15.39 | 7.23 | 1.01 | 0.02 |
| comp7134_c0 | - | 2.04 | 0.55 | 1.89 | 0.02 |
| comp23471_c0 | - | 15.19 | 7.19 | 1.01 | 0.02 |
| comp206007_c0 | elongation factor EF-1 alpha subunit | 0.78 | 2.14 | -1.52 | 0.02 |
| comp15590_c0 | - | 13.69 | 6.71 | 0.97 | 0.02 |
| comp455529_c0 | - | 0.40 | 1.56 | -2.03 | 0.02 |
| comp20037_c3 | alpha-1,4-N-acetylglucosaminyltransferase EXTL3 | 4.73 | 8.87 | -0.98 | 0.02 |
| comp655355_c0 | - | 1.27 | 0.07 | 4.18 | 0.02 |
| comp3858_c0 | bone morphogenetic protein 2/4 | 7.96 | 14.89 | -0.97 | 0.02 |
| comp280346_c0 | - | 3.10 | 0.23 | 3.67 | 0.02 |
| comp3949_c0 | - | 9.43 | 18.77 | -1.05 | 0.02 |
| comp11454_c0 | E74-like factor 3/5 | 13.56 | 6.57 | 0.98 | 0.02 |
| comp16909_c2 | - | 1.28 | 2.95 | -1.28 | 0.02 |
| comp287_c0 | actin related protein 2/3 complex, subunit 1A/1B | 1.73 | 0.34 | 2.29 | 0.02 |
| comp12744_c1 | - | 7.91 | 3.62 | 1.07 | 0.02 |
| comp415334_c0 | - | 0.00 | 2.03 | -inf | 0.02 |
| comp328604_c0 | creatine kinase | 0.00 | 2.35 | -inf | 0.02 |
| comp19461_c1 | beta-site APP-cleaving enzyme 1 (memapsin 2) | 31.49 | 57.35 | -0.94 | 0.02 |
| comp732505_c0 | src homology 2 domain-containing transforming protein C | 1.31 | 0.10 | 3.68 | 0.02 |
| comp18935_c1 | erythrocyte membrane protein band 4.1 | 18.24 | 6.50 | 1.34 | 0.02 |
| comp12992_c2 | Notch | 0.17 | 1.65 | -3.48 | 0.03 |
| comp13900_c0 | hydroxypyruvate isomerase | 7.90 | 15.70 | -1.06 | 0.03 |
| comp7530_c0 | zinc finger and BTB domain-containing protein 16 | 26.46 | 9.99 | 1.35 | 0.03 |
| comp12052_c0 | - | 0.95 | 2.05 | -1.18 | 0.03 |
| comp16145_c0 | 3-hydroxyacyl-CoA dehydrogenase / enoyl-CoA hydratase / 3-hydroxybutyryl-CoA epimerase / enoyl-CoA isomerase [EC:1.1.1.35 | 51.62 | 93.89 | -0.94 | 0.03 |
| comp12772_c0 | - | 3.97 | 1.65 | 1.20 | 0.03 |
| comp7504_c0 | - | 15.71 | 29.77 | -0.98 | 0.03 |
| comp10462_c0 | - | 5.80 | 1.80 | 1.64 | 0.03 |
| comp10293_c1 | protein-tyrosine phosphatase | 2.10 | 0.58 | 1.82 | 0.03 |
| comp4713_c0 | cytidine deaminase | 5.99 | 11.98 | -1.08 | 0.03 |
| comp233_c0 | - | 1.70 | 0.45 | 1.87 | 0.03 |
| comp14960_c0 | peptidyl-prolyl isomerase H (cyclophilin H) | 1.81 | 4.01 | -1.20 | 0.03 |
| comp15582_c0 | - | 4.70 | 8.98 | -1.00 | 0.03 |
| comp293505_c0 | - | 2.37 | 0.48 | 2.29 | 0.03 |
| comp11725_c1 | non-specific serine/threonine protein kinase | 0.68 | 1.62 | -1.31 | 0.03 |
| comp7329_c0 | - | 6.76 | 2.60 | 1.32 | 0.03 |
| comp19985_c2 | - | 336.40 | 171.47 | 0.93 | 0.03 |
| comp18509_c0 | activating transcription factor 2 | 6.87 | 0.70 | 3.67 | 0.03 |
| comp14932_c0 | long-chain acyl-CoA synthetase | 2.08 | 0.48 | 2.03 | 0.03 |
| comp2883_c0 | - | 1.75 | 3.76 | -1.18 | 0.03 |
| comp19593_c1 | - | 21.39 | 4.46 | 2.20 | 0.03 |
| comp16416_c0 | - | 5.57 | 0.86 | 2.60 | 0.03 |
| comp19241_c4 | - | 2.31 | 5.15 | -1.21 | 0.03 |
| comp538857_c0 | - | 1.52 | 0.14 | 3.47 | 0.03 |
| comp19348_c0 | - | 1.76 | 0.18 | 3.48 | 0.03 |
| comp13016_c1 | Rap guanine nucleotide exchange factor (GEF) 1 | 1.18 | 2.35 | -1.05 | 0.03 |
| comp15265_c0 | knypek | 2.64 | 5.58 | -1.13 | 0.03 |
| comp16833_c0 | 3',5'-cyclic-nucleotide phosphodiesterase | 1.69 | 3.28 | -1.01 | 0.03 |
| comp12527_c0 | putative methylase | 2.19 | 4.44 | -1.08 | 0.03 |
| comp236116_c0 | - | 1.42 | 0.08 | 4.16 | 0.03 |
| comp16927_c0 | - | 37.42 | 9.08 | 2.01 | 0.03 |
| comp17605_c0 | - | 4.19 | 0.36 | 3.51 | 0.03 |
| comp10067_c0 | - | 3.57 | 1.06 | 1.70 | 0.03 |
| comp10061_c1 | - | 0.58 | 1.80 | -1.70 | 0.03 |
| comp15644_c1 | - | 1.52 | 3.62 | -1.34 | 0.03 |
| comp10260_c0 | - | 2.81 | 1.26 | 1.09 | 0.03 |
| comp3100_c0 | - | 1.14 | 4.46 | -2.01 | 0.03 |
| comp14558_c0 | tripartite motif-containing protein 8 | 4.36 | 7.99 | -0.95 | 0.03 |
| comp12613_c1 | - | 108.00 | 26.89 | 1.97 | 0.03 |
| comp17561_c4 | - | 9.76 | 16.33 | -0.94 | 0.03 |
| comp8606_c0 | - | 7.62 | 3.42 | 1.09 | 0.03 |
| comp17900_c0 | - | 5.37 | 0.55 | 3.17 | 0.03 |
| comp20041_c1 | - | 7.84 | 14.40 | -0.92 | 0.03 |
| comp8827_c0 | - | 4.11 | 1.60 | 1.29 | 0.03 |
| comp17343_c0 | Wiskott-Aldrich syndrome protein | 2.59 | 5.09 | -0.96 | 0.03 |
| comp15072_c0 | - | 11.20 | 5.36 | 0.99 | 0.03 |
| comp18308_c0 | - | 8.70 | 2.86 | 1.53 | 0.03 |
| comp7377_c0 | - | 2.96 | 6.43 | -1.17 | 0.03 |
| comp7489_c1 | - | 1.22 | 0.13 | 3.16 | 0.03 |
| comp13235_c0 | - | 4.83 | 2.28 | 1.03 | 0.03 |
| comp20137_c0 | - | 3.83 | 8.25 | -1.16 | 0.03 |
| comp3757_c0 | cytochrome P450, family 26, subfamily A | 2.38 | 0.96 | 1.25 | 0.03 |
| comp13213_c0 | large subunit ribosomal protein L22e | 9.66 | 4.67 | 0.97 | 0.03 |
| comp4427_c0 | Ras-related protein Rap-2C | 1.68 | 3.39 | -1.07 | 0.03 |
| comp4338_c0 | - | 7.07 | 0.78 | 3.17 | 0.03 |
| comp14214_c0 | - | 1.85 | 0.00 | inf | 0.03 |
| comp7767_c0 | - | 66.94 | 24.50 | 1.41 | 0.03 |
| comp453680_c0 | - | 2.13 | 0.25 | 3.04 | 0.03 |
| comp15891_c0 | - | 2.61 | 0.82 | 1.62 | 0.03 |
| comp18057_c0 | RNA 3'-terminal phosphate cyclase-like protein | 52.31 | 27.17 | 0.93 | 0.03 |
| comp18334_c1 | - | 13.84 | 7.57 | 0.94 | 0.03 |
| comp15265_c1 | - | 2.30 | 4.48 | -1.02 | 0.03 |
| comp8608_c0 | beta-1,3-N-acetylglucosaminyltransferase 5 | 3.66 | 7.21 | -1.04 | 0.03 |
| comp8733_c1 | - | 4.12 | 1.54 | 1.37 | 0.03 |
| comp16416_c1 | - | 3.45 | 0.54 | 2.81 | 0.03 |
| comp11075_c0 | - | 10.61 | 20.96 | -1.15 | 0.03 |
| comp12937_c0 | - | 4.42 | 8.59 | -1.03 | 0.03 |
| comp20003_c0 | - | 43.47 | 23.32 | 0.94 | 0.03 |
| comp160077_c0 | - | 2.82 | 0.51 | 2.40 | 0.03 |
| comp19099_c1 | - | 5.66 | 2.56 | 1.08 | 0.03 |
| comp48570_c0 | - | 2.01 | 3.95 | -1.02 | 0.03 |
| comp6701_c0 | - | 2.93 | 0.60 | 2.23 | 0.03 |
| comp308555_c0 | - | 3.07 | 0.00 | inf | 0.03 |
| comp2819_c0 | KRAB domain-containing zinc finger protein | 1.72 | 3.86 | -1.25 | 0.03 |
| comp9138_c1 | - | 2.44 | 0.54 | 2.10 | 0.03 |
| comp19739_c0 | - | 9.20 | 1.22 | 2.85 | 0.03 |
| comp14987_c0 | - | 40.50 | 73.34 | -0.91 | 0.03 |
| comp244564_c0 | - | 2.64 | 0.88 | 1.54 | 0.03 |
| comp15373_c0 | major histocompatibility complex, class I | 5.86 | 2.85 | 0.98 | 0.03 |
| comp5650_c0 | - | 2.89 | 0.37 | 2.91 | 0.03 |
| comp8751_c0 | - | 79.26 | 16.56 | 2.19 | 0.03 |
| comp311197_c0 | - | 1.65 | 0.34 | 2.28 | 0.03 |
| comp381165_c0 | - | 1.57 | 0.40 | 1.90 | 0.03 |
| comp5418_c1 | - | 4.03 | 0.55 | 2.84 | 0.03 |
| comp20012_c0 | - | 1.48 | 0.00 | inf | 0.03 |
| comp12923_c0 | glycerol-3-phosphate dehydrogenase (NAD+) | 2.86 | 5.45 | -0.99 | 0.03 |
| comp15267_c0 | - | 0.27 | 1.80 | -2.75 | 0.03 |
| comp442752_c0 | - | 1.90 | 0.13 | 3.83 | 0.03 |
| comp15161_c0 | - | 15.76 | 0.98 | 3.94 | 0.03 |
| comp10047_c0 | - | 1.41 | 3.01 | -1.16 | 0.03 |
| comp117_c0 | - | 1.50 | 0.00 | inf | 0.03 |
| comp9097_c0 | - | 3.60 | 1.55 | 1.13 | 0.03 |
| comp11735_c1 | - | 8.94 | 0.64 | 3.80 | 0.03 |
| comp18391_c0 | protein-serine/threonine kinase | 166.69 | 28.36 | 2.50 | 0.03 |
| comp19647_c1 | - | 14.09 | 6.88 | 0.92 | 0.03 |
| comp17891_c0 | - | 1.15 | 2.25 | -1.04 | 0.03 |
| comp384529_c0 | - | 0.29 | 2.11 | -2.87 | 0.03 |
| comp13149_c0 | ADP-ribosylation factor 6 | 2.26 | 4.67 | -1.10 | 0.03 |
| comp10767_c0 | discs large protein | 4.41 | 1.91 | 1.14 | 0.03 |
| comp19493_c0 | - | 4.82 | 1.73 | 1.42 | 0.03 |
| comp13956_c1 | - | 5.77 | 2.38 | 1.21 | 0.03 |
| comp10326_c0 | - | 0.99 | 2.70 | -1.51 | 0.03 |
| comp6584_c0 | - | 1.77 | 0.65 | 1.40 | 0.04 |
| comp16578_c0 | - | 4.36 | 1.71 | 1.31 | 0.04 |
| comp3109_c0 | - | 2.05 | 4.38 | -1.16 | 0.04 |
| comp12360_c0 | - | 0.68 | 1.84 | -1.48 | 0.04 |
| comp19256_c0 | - | 23.52 | 9.99 | 1.21 | 0.04 |
| comp47206_c0 | - | 10.73 | 4.23 | 1.28 | 0.04 |
| comp9969_c0 | - | 0.89 | 2.87 | -1.76 | 0.04 |
| comp18679_c1 | - | 91.95 | 47.48 | 0.90 | 0.04 |
| comp728_c0 | - | 1.37 | 0.04 | 4.97 | 0.04 |
| comp306479_c0 | - | 1.64 | 0.11 | 3.79 | 0.04 |
| comp19097_c0 | - | 4.55 | 1.98 | 1.13 | 0.04 |
| comp5616_c0 | basic amino acid/polyamine antiporter, APA family | 0.94 | 2.22 | -1.30 | 0.04 |
| comp19972_c0 | putative transposase | 6.64 | 0.54 | 3.69 | 0.04 |
| comp583803_c0 | - | 1.55 | 0.07 | 4.45 | 0.04 |
| comp14207_c0 | - | 1.15 | 2.71 | -1.28 | 0.04 |
| comp9733_c0 | - | 1.41 | 3.10 | -1.24 | 0.04 |
| comp18954_c1 | - | 7.10 | 3.03 | 1.18 | 0.04 |
| comp15587_c0 | SMAD, mothers against DPP 2/3 | 7.09 | 3.49 | 0.97 | 0.04 |
| comp19493_c3 | - | 12.60 | 5.65 | 1.05 | 0.04 |
| comp7641_c0 | - | 41.34 | 20.31 | 0.98 | 0.04 |
| comp587254_c0 | - | 1.65 | 0.02 | 6.41 | 0.04 |
| comp8640_c0 | SWI/SNF-related matrix-associated actin-dependent regulator of chromatin subfamily D | 3.30 | 6.28 | -1.00 | 0.04 |
| comp13518_c0 | - | 3.50 | 1.72 | 0.97 | 0.04 |
| comp19526_c0 | 3-mercaptopyruvate sulfurtransferase | 44.13 | 19.22 | 0.90 | 0.04 |
| comp14912_c0 | - | 1.36 | 4.65 | -1.84 | 0.04 |
| comp17707_c0 | vascular endothelial growth factor A/B, PGF | 9.03 | 4.41 | 0.95 | 0.04 |
| comp19860_c0 | E3 ubiquitin-protein ligase RNF128 | 15.08 | 26.00 | -0.91 | 0.04 |
| comp6388_c0 | - | 9.42 | 3.88 | 1.22 | 0.04 |
| comp13162_c0 | - | 1.62 | 3.10 | -1.00 | 0.04 |
| comp404892_c0 | DNA mismatch repair protein MSH2 | 0.49 | 1.83 | -1.95 | 0.04 |
| comp14178_c0 | pre-mRNA-processing factor 4 | 2.72 | 5.36 | -1.02 | 0.04 |
| comp4170_c0 | - | 10.35 | 4.04 | 1.29 | 0.04 |
| comp315779_c0 | - | 2.19 | 0.50 | 2.12 | 0.04 |
| comp20131_c4 | - | 11.11 | 5.82 | 0.92 | 0.04 |
| comp20153_c0 | - | 10.91 | 5.21 | 0.95 | 0.04 |
| comp16728_c1 | 3',5'-cyclic-nucleotide phosphodiesterase | 17.76 | 9.28 | 0.91 | 0.04 |
| comp19562_c1 | transcription factor Dp-1 | 9.03 | 3.60 | 1.14 | 0.04 |
| comp13726_c1 | molecular chaperone HtpG | 17.17 | 7.02 | 1.25 | 0.04 |
| comp5834_c0 | translation initiation factor eIF-4E | 0.75 | 2.19 | -1.59 | 0.04 |
| comp12580_c0 | - | 24.95 | 12.51 | 0.93 | 0.04 |
| comp10627_c0 | dystrophin | 4.61 | 2.27 | 0.96 | 0.04 |
| comp15081_c2 | 2-oxoglutarate dehydrogenase E1 component | 5.28 | 0.91 | 2.50 | 0.04 |
| comp18604_c0 | - | 5.25 | 10.26 | -1.10 | 0.04 |
| comp3678_c0 | - | 1.30 | 3.08 | -1.31 | 0.04 |
| comp12140_c0 | wingless-type MMTV integration site family, member 11 | 4.76 | 1.97 | 1.22 | 0.04 |
| comp15768_c0 | - | 1.89 | 3.88 | -1.12 | 0.04 |
| comp18390_c0 | mitochondrial carrier protein, MC family | 25.20 | 9.21 | 1.41 | 0.04 |
| comp22279_c0 | - | 16.71 | 30.61 | -0.94 | 0.04 |
| comp10901_c1 | - | 5.80 | 1.96 | 1.48 | 0.04 |
| comp19285_c0 | - | 2.72 | 0.99 | 1.39 | 0.04 |
| comp15858_c0 | sphingosine kinase | 5.83 | 2.95 | 0.94 | 0.04 |
| comp20116_c0 | chloride channel 7 | 142.68 | 68.75 | 1.01 | 0.04 |
| comp5912_c0 | - | 0.99 | 2.42 | -1.34 | 0.04 |
| comp12654_c2 | SAR1A | 40.13 | 71.48 | -0.89 | 0.04 |
| comp211386_c0 | phosphatidylinositol glycan, class C | 0.96 | 2.16 | -1.24 | 0.04 |
| comp19985_c0 | - | 9.81 | 2.97 | 1.69 | 0.04 |
| comp9202_c0 | - | 1.93 | 0.15 | 3.66 | 0.04 |
| comp392467_c0 | histone-lysine N-methyltransferase SETDB | 0.58 | 1.28 | -1.24 | 0.04 |
| comp18021_c0 | human immunodeficiency virus type I enhancer-binding protein | 4.36 | 2.24 | 0.91 | 0.04 |
| comp18992_c1 | ATP-binding cassette, subfamily C (CFTR/MRP), member 5 | 17.02 | 8.10 | 0.90 | 0.04 |
| comp9151_c0 | - | 0.65 | 2.68 | -2.08 | 0.04 |
| comp17387_c1 | - | 7.41 | 3.58 | 1.00 | 0.04 |
| comp19049_c1 | - | 42.03 | 21.67 | 0.90 | 0.04 |
| comp8127_c0 | - | 12.57 | 6.05 | 1.00 | 0.04 |
| comp17626_c1 | - | 0.52 | 2.20 | -2.13 | 0.04 |
| comp7495_c0 | glycerate kinase | 10.16 | 18.02 | -0.89 | 0.04 |
| comp8522_c0 | - | 20.29 | 4.60 | 2.08 | 0.04 |
| comp116444_c0 | [acyl-carrier-protein] S-malonyltransferase | 0.98 | 2.22 | -1.23 | 0.04 |
| comp16071_c0 | - | 23.74 | 12.21 | 0.89 | 0.04 |
| comp11040_c1 | - | 13.22 | 5.29 | 1.27 | 0.04 |
| comp14894_c3 | - | 5.23 | 2.38 | 1.09 | 0.04 |
| comp9697_c0 | elongation factor EF-1 alpha subunit | 0.75 | 2.29 | -1.71 | 0.04 |
| comp12955_c0 | beta-glucosidase | 2.99 | 5.43 | -0.92 | 0.04 |
| comp13625_c0 | - | 2.46 | 4.66 | -1.00 | 0.04 |
| comp381775_c0 | - | 0.36 | 1.53 | -2.15 | 0.04 |
| comp17508_c2 | - | 7.45 | 3.35 | 1.36 | 0.04 |
| comp304051_c0 | ataxin-7 | 0.51 | 1.97 | -1.99 | 0.04 |
| comp18474_c0 | heat shock 70kDa protein 4 | 34.55 | 17.54 | 0.93 | 0.04 |
| comp8220_c0 | - | 7.18 | 3.49 | 0.99 | 0.04 |
| comp17119_c0 | - | 4.94 | 2.44 | 0.99 | 0.04 |
| comp3438_c0 | - | 3.77 | 1.05 | 1.81 | 0.04 |
| comp13182_c0 | tubulin gamma | 2.06 | 4.06 | -1.04 | 0.04 |
| comp15426_c0 | polo-like kinase 2 | 7.19 | 3.61 | 0.93 | 0.04 |
| comp10092_c0 | - | 2.85 | 0.55 | 2.27 | 0.04 |
| comp16164_c0 | claudin | 53.02 | 93.46 | -0.87 | 0.04 |
| comp20012_c1 | KRAB and SCAN domains-containing zinc finger protein | 10.81 | 15.26 | -0.89 | 0.04 |
| comp815127_c0 | - | 1.26 | 0.00 | inf | 0.04 |
| comp18805_c0 | - | 7.69 | 13.88 | -0.92 | 0.04 |
| comp10225_c1 | - | 1.25 | 2.87 | -1.26 | 0.04 |
| comp15907_c0 | nuclear receptor, subfamily 1, group D, member 1 | 1.70 | 4.69 | -1.50 | 0.04 |
| comp13958_c1 | - | 1.00 | 3.18 | -1.73 | 0.04 |
| comp9331_c0 | - | 0.90 | 2.00 | -1.20 | 0.04 |
| comp415866_c0 | - | 1.68 | 0.28 | 2.52 | 0.05 |
| comp4443_c0 | - | 1.74 | 3.40 | -1.04 | 0.05 |
| comp12899_c0 | ribose-phosphate pyrophosphokinase | 2.20 | 4.28 | -1.02 | 0.05 |
| comp9263_c1 | cyclin D1 | 1.99 | 3.70 | -0.97 | 0.05 |
| comp7210_c0 | - | 2.92 | 5.43 | -0.96 | 0.05 |
| comp9284_c0 | - | 2.10 | 4.18 | -1.06 | 0.05 |
| comp19668_c0 | ornithine--oxo-acid transaminase | 18.99 | 9.95 | 0.87 | 0.05 |
| comp2893_c0 | - | 4.09 | 1.05 | 1.88 | 0.05 |
| comp346616_c0 | - | 0.45 | 1.67 | -1.95 | 0.05 |
| comp17606_c1 | - | 7.94 | 3.82 | 1.00 | 0.05 |
| comp768819_c0 | kinesin family member 18/19 | 1.31 | 0.10 | 3.60 | 0.05 |
| comp19882_c0 | - | 9.41 | 4.73 | 0.94 | 0.05 |
| comp31463_c0 | - | 7.63 | 13.99 | -0.94 | 0.05 |
| comp10058_c0 | - | 1.91 | 0.32 | 2.49 | 0.05 |
| comp17309_c0 | - | 1.53 | 2.99 | -1.03 | 0.05 |
| comp2895_c0 | chromobox protein 7 | 3.59 | 1.71 | 0.99 | 0.05 |
| comp17725_c0 | MFS transporter, MCP family, solute carrier family 16 (monocarboxylic acid transporters), member 7 | 10.18 | 23.48 | -1.25 | 0.05 |
| comp17822_c0 | 5-aminolevulinate synthase | 176.66 | 94.00 | 0.86 | 0.05 |
| comp12130_c0 | nucleophosmin 1 | 2.64 | 4.83 | -0.94 | 0.05 |
| comp8107_c0 | - | 1.99 | 0.53 | 1.85 | 0.05 |
| comp20020_c0 | - | 2.30 | 0.13 | 4.01 | 0.05 |
| comp8993_c0 | forkhead box protein O1 | 2.70 | 1.16 | 1.16 | 0.05 |
| comp5802_c0 | NADPH oxidase | 2.14 | 0.54 | 1.93 | 0.05 |
| comp182877_c0 | - | 0.80 | 2.41 | -1.63 | 0.05 |
| comp36082_c0 | - | 5.87 | 2.48 | 1.19 | 0.05 |
| comp17041_c0 | - | 23.85 | 0.06 | 8.85 | 0.05 |
| comp665868_c0 | integrin alpha 6 | 1.27 | 0.29 | 2.08 | 0.05 |
| comp5675_c0 | - | 3.63 | 0.91 | 1.92 | 0.05 |
| comp8360_c0 | - | 4.60 | 8.81 | -1.00 | 0.05 |
| comp15672_c0 | - | 11.92 | 5.83 | 0.99 | 0.05 |
| comp11372_c0 | - | 2.40 | 4.96 | -1.10 | 0.05 |
| comp3335_c0 | - | 3.69 | 1.38 | 1.37 | 0.05 |
| comp7533_c0 | - | 31.90 | 16.16 | 0.92 | 0.05 |
| comp106330_c0 | - | 1.76 | 0.33 | 2.29 | 0.05 |
| comp19117_c1 | - | 9.91 | 2.92 | 1.72 | 0.05 |
| comp15353_c1 | ubiquitin carboxyl-terminal hydrolase 25/28 | 24.11 | 12.75 | 0.87 | 0.05 |
| comp11038_c0 | - | 2.83 | 1.37 | 0.98 | 0.05 |
| comp17278_c0 | - | 2.66 | 4.81 | -0.90 | 0.05 |
| comp12253_c0 | - | 1.71 | 0.29 | 2.53 | 0.05 |

Table S5 Overview of reads for sRNA-seq from raw data to high quality reads, and quality filtering.

|  |  | **P0_a** |  |  |  | **P0_b** |  |  |  | **P0_c** |  |  |  |
| --- | --- | --- | --- | --- | --- | --- | --- | --- | --- | --- | --- | --- | --- |
| **lib** | type | Total | % of Total | uniq | % of uniq | Total | % of Total | uniq | % of uniq | Total | % of Total | uniq | % of uniq |
| **Raw reads** | NA | 10,645,238 | 100.00 | 781,670 | 100 | 12,000,222 | 100.00 | 649,199 | 100.00 | 10,938,770 | 100.00 | 935,118 | 100.00 |
| **3ADT&length filter** | Sequence type | 1,406,305 | 13.21 | 345,557 | 44.21 | 684,242 | 5.70 | 283,755 | 43.71 | 3,751,144 | 34.29 | 631,375 | 67.52 |
| **Junk reads** | Sequence type | 12,607 | 0.12 | 4,163 | 0.53 | 10,786 | 0.09 | 3,761 | 0.58 | 6,887 | 0.06 | 3,269 | 0.35 |
| **Rfam** | RNA class | 968,386 | 9.10 | 39,423 | 5.04 | 644,001 | 5.37 | 31,181 | 4.80 | 370,148 | 3.38 | 26,730 | 2.86 |
| **Repeats** | RNA class | 1,437 | 0.01 | 384 | 0.05 | 811 | 0.01 | 306 | 0.05 | 333 | 0.00 | 174 | 0.02 |
| **valid reads** | Sequence type | 8,256,726 | 77.56 | 392,297 | 50.19 | 10,660,530 | 88.84 | 330,314 | 50.88 | 6,810,317 | 62.26 | 273,623 | 29.26 |
|  |  |  |  |  |  |  |  |  |  |  |  |  |  |
| **rRNA** | RNA class | 816,961 | 7.67 | 21,758 | 0.2 | 530,989 | 4.42 | 16,788 | 0.14 | 291,423 | 2.66 | 15,661 | 0.14 |
| **tRNA** | RNA class | 54,828 | 0.52 | 7,125 | 0.07 | 43,825 | 0.37 | 5,712 | 0.05 | 41,764 | 0.38 | 4,732 | 0.04 |
| **snoRNA** | RNA class | 17,591 | 0.17 | 2,458 | 0.02 | 18,245 | 0.15 | 2,028 | 0.02 | 8,689 | 0.08 | 1,238 | 0.01 |
| **snRNA** | RNA class | 9,715 | 0.09 | 2,878 | 0.03 | 8,010 | 0.07 | 2,440 | 0.02 | 5,150 | 0.05 | 1,737 | 0.02 |
| **other Rfam RNA** | RNA class | 69,291 | 0.65 | 5,204 | 0.05 | 42,932 | 0.36 | 4,213 | 0.04 | 23,122 | 0.21 | 3,362 | 0.03 |

|  |  | **P4_a** |  |  |  | **P4_b** |  |  |  | **P4_c** |  |  |  |
| --- | --- | --- | --- | --- | --- | --- | --- | --- | --- | --- | --- | --- | --- |
| **lib** | type | Total | % of Total | uniq | % of uniq | Total | % of Total | uniq | % of uniq | Total | % of Total | uniq | % of uniq |
| **Raw reads** | NA | 12,000,222 | 100.00 | 603,412 | 100.00 | 10,487,994 | 100.00 | 497,103 | 100.00 | 13,328,741 | 100.00 | 964,817 | 100.00 |
| **3ADT&length filter** | Sequence type | 789,459 | 6.58 | 273,895 | 45.39 | 467,301 | 4.46 | 199,476 | 40.13 | 3,007,139 | 22.56 | 613,257 | 63.56 |
| **Junk reads** | Sequence type | 9,708 | 0.08 | 2,998 | 0.50 | 7,636 | 0.07 | 2,676 | 0.54 | 7,520 | 0.06 | 3,306 | 0.34 |
| **Rfam** | RNA class | 713,818 | 5.95 | 30,186 | 5.00 | 505,540 | 4.82 | 27,533 | 5.54 | 612,791 | 4.60 | 30,167 | 3.13 |
| **Repeats** | RNA class | 1,037 | 0.01 | 342 | 0.06 | 794 | 0.01 | 306 | 0.06 | 541 | 0.00 | 243 | 0.03 |
| **valid reads** | Sequence type | 10,486,414 | 87.39 | 296,161 | 49.08 | 9,506,894 | 90.65 | 267,240 | 53.76 | 9,700,859 | 72.78 | 317,938 | 32.95 |
|  |  |  |  |  |  |  |  |  |  |  |  |  |  |
| **rRNA** | RNA class | 607,925 | 5.07 | 16,228 | 0.14 | 419,278 | 4.00 | 14,629 | 0.14 | 511,870 | 3.84 | 17,338 | 0.13 |
| **tRNA** | RNA class | 40,964 | 0.34 | 5,270 | 0.04 | 40,907 | 0.39 | 5,137 | 0.05 | 47,012 | 0.35 | 5,031 | 0.04 |
| **snoRNA** | RNA class | 12,350 | 0.10 | 1,939 | 0.02 | 9,982 | 0.10 | 1,759 | 0.02 | 9,652 | 0.07 | 1,541 | 0.01 |
| **snRNA** | RNA class | 7,194 | 0.06 | 2,345 | 0.02 | 6,377 | 0.06 | 2,095 | 0.02 | 5,763 | 0.04 | 2,095 | 0.02 |
| **other Rfam RNA** | RNA class | 45,385 | 0.38 | 4,404 | 0.04 | 28,996 | 0.28 | 3,913 | 0.04 | 38,494 | 0.29 | 4,162 | 0.03 |

| Overview of reads from raw data to cleaned sequences. |
| --- |
| 3ADT&length filter: reads removed due to 3ADT not found and length with <18 nt and >25 nt were removed(for plants); length with<18 and >26 were remove(for animals) |
| Junk reads:Junk: >=2N, >=7A, >=8C, >=6G, >=7T, >=10Dimer, >=6Trimer, or >=5Tetramer |
| Rfam:Collection of many common non-coding RNA families except micro RNA; http://rfam.janelia.org |
| Repeats:Prototypic sequences representing repetitive DNA from different eukaryotic species; http://www.girinst.org/repbase. |
| Notes:valid reads may not be equal to raw reads - 3ADT&length filter - Junk reads ¨C mRNA ¨C Rfam - Repeats, because there are overlapped sequences between mRNA£¬Rfam and Repeats, details please refer to _comp_others.txt in fold 2_MappedData. |

Table S6 List of know-miRNA and novel-miRNA of P. vachelli.

| miR_name | miR_seq | group |
| --- | --- | --- |
| ssa-miR-122-5p_R-1 | TGGAGTGTGACAATGGTGTTT | gp1 |
| ssa-miR-122-2-3p_R-1 | AACGCCATTATCACACTAAAT | gp1 |
| ipu-miR-199a-5p | CCCAGTGTTCAGACTACCTGTTC | gp1 |
| ssa-miR-199a-3p_1ss10TC | ACAGTAGTCCGCACATTGGTT | gp1 |
| dre-miR-451_R-1 | AAACCGTTACCATTACTGAGT | gp1 |
| dre-mir-451-p3 | TTTAGTAATGGTAAGGGTTCT | gp1 |
| ssc-mir-1285-p5_1ss23CA | GTGGGATCGCGCCTGTGAATAGAC | gp1 |
| ssc-mir-1285-p3 | CACTGTACTCCAGCCTGGGCAACA | gp1 |
| dre-miR-1306_R-1 | CCACCTCCCCTGCAAACGTCC | gp1 |
| bta-mir-2887-2-p5 | CGGGGTCCGGTGCGGAGAGC | gp1 |
| bta-mir-2887-2-p3 | CGGAGAGCCCTTCGTCCCGGGAC | gp1 |
| bta-mir-3533-p5_1ss2TC | GCGGCATCCATGAGACCACCTTCAAC | gp1 |
| bta-mir-3533-p3_1ss20TC | TCAACTCCATCATGAAGTGCGACG | gp1 |
| ipu-miR-3618 | GATTTCCAATAATTGAGACAGT | gp1 |
| hsa-miR-3618_1ss21GA | TGTCTACATTAATGAAAAGAAC | gp1 |
| ipu-mir-7560-p3_1ss1AG | GATGGCACACTGTAGTTTCCTGAGG | gp1 |
| ipu-miR-122_R+3 | TGGAGTGTGACAATGGTGTTTGTA | gp2 |
| ssa-miR-122-3p_R+1_2ss12TC19TA | AACGCCATTATCACACTAAATAT | gp2 |
| dre-miR-451_R-3_1ss19AT | AAACCGTTACCATTACTGT | gp2 |
| aca-miR-451-3p_L+2R+1 | TTTAGTAATGGTAAGGGTTCTC | gp2 |
| mmu-mir-466i-p5 | TGTGTGTGTGTGTGTGTATATATA | gp2 |
| mmu-mir-466i-p3_1ss21TC | TATATATATACACACACACACA | gp2 |
| mmu-miR-466i-5p_L+1R+3_1ss13GA | GTGTGTGTGTGTATGTGTGTGTAT | gp2 |
| gga-miR-466_R-1_3ss20AC21GA22AC | ATATATACACACACACATACAC | gp2 |
| mmu-mir-466i-p3_1ss10GA | TGTGTGTGTATGTATATATATA | gp2 |
| mmu-mir-467g-p5_1ss13TC | TGTGTGTGTGTACATATATAT | gp2 |
| mmu-mir-669f-p5 | ATACATATACATACACACACA | gp2 |
| ssa-miR-722-3p_R-3_1ss19AT | TTTGCAGAAACGTTTCAGT | gp2 |
| ssa-miR-722-3p_R-3_1ss19AT | TTTGCAGAAACGTTTCAGT | gp2 |
| mmu-mir-1187-p5_1ss11AG | ACACACACATGTATATATATAT | gp2 |
| mmu-mir-467g-p3_1ss17TC | TATATATATGTGTGTGCGT | gp2 |
| mmu-mir-1187-p5_1ss11AG | ACACACACATGTATATATATAT | gp2 |
| mmu-mir-467g-p3_1ss17TC | TATATATATGTGTGTGCGT | gp2 |
| aca-miR-1a-2-5p_R+3 | ACATACTTCTTTATATGCCCATA | gp3 |
| aca-miR-1a-3p | TGGAATGTAAAGAAGTATGTAT | gp3 |
| aca-miR-1a-2-5p_R+3 | ACATACTTCTTTATATGCCCATA | gp3 |
| aca-miR-1a-3p | TGGAATGTAAAGAAGTATGTAT | gp3 |
| pma-miR-1a-3p_R-1_1ss18CT | TGGAATGTAAAGAAGTATGTT | gp3 |
| ccr-miR-7b_R+1 | TGGAAGACTTGTGATTTTGTTGTT | gp3 |
| rno-miR-7a-5p | TGGAAGACTAGTGATTTTGTTGT | gp3 |
| aca-miR-9-5p_R+3 | TCTTTGGTTATCTAGCTGTATGA | gp3 |
| aca-miR-9-1-3p_R+2 | ATAAAGCTAGATAACCGAAAGT | gp3 |
| ccr-miR-10c_R-1 | TACCCTGTAGATCCGGATTTGT | gp3 |
| dre-miR-10c-3p_L+1R-1 | CAAATTCGTATCTAGGGGAGT | gp3 |
| dre-miR-10b-5p_R-1 | TACCCTGTAGAACCGAATTTGT | gp3 |
| dre-miR-10b-3p_L-1 | CAGATTCGATTCTAGGGGAGT | gp3 |
| ccr-miR-15b | TAGCAGCACATCATGGTTTGT | gp3 |
| pma-miR-15b_R-1_1ss21CT | TAGCAGCACATCATGGATTGT | gp3 |
| dre-miR-15c_R+1_1ss10GA | AAGCAGCGCATCATGGTTTTCA | gp3 |
| fru-miR-16 | TAGCAGCACGTAAATATTGGAG | gp3 |
| ssa-miR-16a-3p_R+1_2ss10TA11TC | CCAGTATTGACCGTGCTGCTGAA | gp3 |
| tgu-miR-16b-5p | TAGCAGCACGTAAATATTGGAG | gp3 |
| ipu-miR-16c_R+1_1ss1CT | TCAGCAGCACGGTCAATACTGG | gp3 |
| ccr-miR-17-5p | CAAAGTGCTTACAGTGCAGGTAG | gp3 |
| ccr-miR-17-3p | ACTGCAGTGGAGGCACTTCTAGC | gp3 |
| ccr-miR-17-5p | CAAAGTGCTTACAGTGCAGGTAG | gp3 |
| ccr-miR-17-3p | ACTGCAGTGGAGGCACTTCTAGC | gp3 |
| dre-miR-18a_1ss11CT | TAAGGTGCATTTAGTGCAGATA | gp3 |
| ssa-miR-18a-3p_R-3_2ss10GA11TG | ACTGCCCTAAGTGCTCCTTCT | gp3 |
| ccr-miR-18c_R-3 | TAAGGTGCATCTTGTGTAGT | gp3 |
| ssa-miR-19c-4-5p_R+1_2ss17TA21AT | AGTTTTGCTGGTTTGCATTCTGC | gp3 |
| dre-miR-19b-3p_R-1 | TGTGCAAATCCATGCAAAACTG | gp3 |
| ssa-miR-19c-4-5p_R+1_2ss17TA21AT | AGTTTTGCTGGTTTGCATTCTGC | gp3 |
| ssa-miR-19c-3p | TGTGCAAATCCATGCAAAACTG | gp3 |
| aca-miR-19a-5p_L-1R+1_1ss20TC | AGTTTTGCATAGTTGCACCAC | gp3 |
| aca-miR-19a-3p_R+1 | TGTGCAAATCTATGCAAAACTGA | gp3 |
| ssa-miR-20a-5p | TAAAGTGCTTATAGTGCAGGTAG | gp3 |
| ssa-miR-20a-2-3p_R-1 | ACTGCAGTGTGAGCACTTGAAG | gp3 |
| fru-miR-21 | TAGCTTATCAGACTGGTGTTGGC | gp3 |
| ssa-miR-21b-3p_L-1R+1 | GACAACAGTCTGTAGGCTGTCT | gp3 |
| mdo-miR-21-5p_L+1_1ss17AG | ATAGCTTATCAGACTGGTGTTGA | gp3 |
| mmu-miR-21c_R-2_1ss19CA | TAGCTTATCAGACTGGTAA | gp3 |
| dre-miR-22a-5p_R-1 | AGTTCTTCACTGGCAAGCTTT | gp3 |
| dre-miR-22a-3p | AAGCTGCCAGCTGAAGAACTGT | gp3 |
| pma-miR-23b_R-2 | ATCACATTGCCAGGGATTACC | gp3 |
| ssa-miR-24b-5p_1ss17AT | TGCCTACTGAGCTGATTACAGT | gp3 |
| dre-miR-24_R+1 | TGGCTCAGTTCAGCAGGAACAGT | gp3 |
| ssa-miR-25-5p_2ss9TA23CT | AGGCGGAGACTTGGGCAATTGCT | gp3 |
| bta-miR-25 | CATTGCACTTGTCTCGGTCTGA | gp3 |
| pma-miR-25b-3p_R-2_1ss10AT | CATTGCACTTGTCTCAGTCT | gp3 |
| dre-miR-26a-5p | TTCAAGTAATCCAGGATAGGCT | gp3 |
| dre-miR-26a-2-3p_4ss4AG8AT11AG19CT | CCTGTTCTTGGTTACTTGTACT | gp3 |
| hsa-miR-26a-5p | TTCAAGTAATCCAGGATAGGCT | gp3 |
| hsa-miR-26a-2-3p_R+1_1ss20TG | CCTATTCTTGATTACTTGTGTCT | gp3 |
| dre-miR-27b-5p_R-1 | AGAGCTTAGCTGATTGGTGAAC | gp3 |
| dre-miR-27b-3p_R-1 | TTCACAGTGGCTAAGTTCTGC | gp3 |
| cgr-miR-27a-3p_R+1_1ss19CT | TTCACAGTGGCTAAGTTCTGCT | gp3 |
| tni-miR-27e_R-1_1ss19AT | TTCACAGTGGCTAAGTTCTGT | gp3 |
| bta-miR-28 | AAGGAGCTCACAGTCTATTGAG | gp3 |
| hsa-miR-28-5p | AAGGAGCTCACAGTCTATTGAG | gp3 |
| ssa-miR-29b-2-5p_L-2_2ss12CT20TC | ACTGATTTCTTCTGGTGCTTAGA | gp3 |
| ccr-miR-29a | TAGCACCATTTGAAATCGGTTA | gp3 |
| hsa-miR-29b-2-5p_R+1_1ss18CA | CTGGTTTCACATGGTGGATTAGA | gp3 |
| hsa-miR-29b-3p_R-1 | TAGCACCATTTGAAATCAGTGT | gp3 |
| cgr-miR-29c-5p_1ss11CT | ACCGATTTCTTCTGGTGTTCAGA | gp3 |
| cgr-miR-29c-3p_R+1 | TAGCACCATTTGAAATCGGTTA | gp3 |
| ssa-miR-29b-1-5p_1ss18TC | ACTGATTTCTTCTGGTGCTTAGA | gp3 |
| ssa-miR-29b-3p | TAGCACCATTTGAAATCGGTTA | gp3 |
| ipu-miR-29b_1ss11GA | GCTGAATTCAAATGGTGCCATAGA | gp3 |
| ssa-miR-30a-5p | TGTAAACATCCTACACTCTCAGC | gp3 |
| ssa-miR-30a-4-3p_1ss12GA | CTGGGAGAGGGATGTTTACGCT | gp3 |
| dre-miR-30e-5p_R+2 | TGTAAACATCCTTGACTGGAAGCT | gp3 |
| dre-miR-30e-3p | CTTTCAGTCGGATGTTTGCAGC | gp3 |
| dre-miR-30e-5p_R+2 | TGTAAACATCCTTGACTGGAAGCT | gp3 |
| dre-miR-30e-3p | CTTTCAGTCGGATGTTTGCAGC | gp3 |
| ipu-miR-30d | CTTTCAGTTGGATGTTTGCTGT | gp3 |
| dre-miR-31_L+1R+1 | TGGCAAGATGTTGGCATAGCTGT | gp3 |
| aca-miR-31-5p_R+2_1ss1AT | TGGCAAGATGTTGGCATAGCTGA | gp3 |
| aca-miR-32-5p_R-1 | TATTGCACATTACTAAGTTGC | gp3 |
| gga-miR-33-5p | GTGCATTGTAGTTGCATTGC | gp3 |
| gga-miR-33-3p_L+1_1ss20TC | CAATGTTCCTGCAGTGCAGCA | gp3 |
| ssa-miR-33b-5p_R-1 | GTGCATTGTAGTTGCATTGC | gp3 |
| ssa-miR-33b-3p_R-2 | CAATGTTCCTGCAGTGCAA | gp3 |
| dre-miR-34a | TGGCAGTGTCTTAGCTGGTTGT | gp3 |
| dre-miR-34b_L-1R+1 | AGGCAGTGTTGTTAGCTGATTGT | gp3 |
| hsa-miR-34c-5p | AGGCAGTGTAGTTAGCTGATTGC | gp3 |
| dre-miR-34c-3p_1ss22GC | AATCACTAACCTCACTACCAGC | gp3 |
| bbe-miR-71-5p_R-1 | TGAAAGACATGGGTAGTGAGA | gp3 |
| xtr-miR-92a_R+1 | TATTGCACTTGTCCCGGCCTGT | gp3 |
| dre-miR-92a-3p | TATTGCACTTGTCCCGGCCTGT | gp3 |
| sko-miR-92c_R+2_1ss10CT | TATTGCACTTGTCCCGGCCTGTAT | gp3 |
| sha-miR-92a_R+4 | TATTGCACTTGTCCCGGCCTGT | gp3 |
| bbe-miR-92d-3p_1ss15TC | TATTGCACTTATCCCGGCCTGT | gp3 |
| csa-miR-92c_R-2_1ss9CT | TATTGCACTTGTCCCGGCCG | gp3 |
| cin-miR-92c-3p_1ss18TC | TATTGCACTCGTCCCGGCCTAT | gp3 |
| odi-miR-92b_R-4_1ss10GT | TATTGCACTTGTCCCGAC | gp3 |
| dre-miR-93_R+1 | AAAAGTGCTGTTTGTGCAGGTAG | gp3 |
| dre-miR-96-5p | TTTGGCACTAGCACATTTTTGCT | gp3 |
| dre-miR-96-3p_R+1 | CAATTATGTGTAGTGCCAATATT | gp3 |
| efu-miR-99b_R-3 | CACCCGTAGAACCGACCTTGCG | gp3 |
| ssa-miR-99-5p | AACCCGTAGATCCGATCTTGTG | gp3 |
| bbe-miR-100-5p | AACCCGTAGATCCGAACTTGTG | gp3 |
| bbe-miR-100-3p_1ss10GC | CAAGCTCGTCTCTATGGGTCT | gp3 |
| ssa-miR-101a-5p_R-1_1ss12TA | TCAGTTATCACAGTGCTGATGC | gp3 |
| dre-miR-101a | TACAGTACTGTGATAACTGAAG | gp3 |
| cin-miR-101_R-3_1ss17AC | TACAGTACTGTGATAACTA | gp3 |
| pma-miR-103a_R-2_1ss9CT | AGCAGCATTGTACAGGGCTTG | gp3 |
| cgr-miR-106b-5p_R-2_1ss10GT | TAAAGTGCTTACAGTGCAGA | gp3 |
| cgr-miR-106b-3p_L+2R-2 | TACCGCACTGTGGGTACTTGCT | gp3 |
| ssa-miR-107-5p_R+1_3ss4TC12AG16TC | AGCCTCTTTACGGTGCTGCCTTGT | gp3 |
| fru-miR-103 | AGCAGCATTGTACAGGGCTATGA | gp3 |
| dre-miR-124-5p | CGTGTTCACAGCGGACCTTGAT | gp3 |
| aca-miR-124b_R-1 | TAAGGCACGCGGTGAATGCT | gp3 |
| oha-miR-124-5p | CGTGTTCACAGCGGACCTTGAT | gp3 |
| oha-miR-124-4-3p_R-2 | TAAGGCACGCGGTGAATGCT | gp3 |
| sha-miR-125a | TCCCTGAGACCCTAACTTGTGA | gp3 |
| ssa-miR-125a-2-3p_3ss20CG21GC22CT | ACGGGTTAGGCTCTTGGGAGCT | gp3 |
| ssa-miR-125a-5p | TCCCTGAGACCCTAACTTGTGA | gp3 |
| efu-miR-125a | ACGGGTTAGGCTCTTGGGAGCT | gp3 |
| aca-miR-125a-5p | TCCCTGAGACCCTTAACCTGTG | gp3 |
| bfl-miR-125b_L-2R+2 | ACAAGTTAGGGTCTCAGGGATT | gp3 |
| cin-miR-125-5p_R+1_1ss16AC | TCCCTGAGACCCTAACACGTGA | gp3 |
| ola-miR-126-5p | CATTATTACTTTTGGTACGCG | gp3 |
| ola-miR-126-3p_R+2 | TCGTACCGTGAGTAATAATGCA | gp3 |
| ola-miR-126-5p | CATTATTACTTTTGGTACGCG | gp3 |
| ola-miR-126-3p_R+2 | TCGTACCGTGAGTAATAATGCA | gp3 |
| bta-miR-127 | TCGGATCCGTCTGAGCTTGGCT | gp3 |
| oha-miR-128-3p_R-1 | TCACAGTGAACCGGTCTCTTT | gp3 |
| bta-miR-129_R-1 | CTTTTTGCGGTCTGGGCTTGC | gp3 |
| bta-miR-129-3p | AAGCCCTTACCCCAAAAAGCAT | gp3 |
| bta-miR-129_R-1 | CTTTTTGCGGTCTGGGCTTGC | gp3 |
| bta-miR-129-3p | AAGCCCTTACCCCAAAAAGCAT | gp3 |
| ssa-miR-130d-2-5p_2ss9AT13CT | GCCCTTTTTCTGTTGTACTACT | gp3 |
| ccr-miR-130c_R-1 | CAGTGCAATATTAAAAGGGCA | gp3 |
| dre-miR-130c-5p | GCCCTTTTTCTGTTGTACTACT | gp3 |
| dre-miR-130c-3p_R-1 | CAGTGCAATATTAAAAGGGCA | gp3 |
| ssa-miR-130a-5p_1ss9CT | ACTCTTTCTCTGTTGCACTACT | gp3 |
| ssa-miR-130a-2-3p_R-1 | CAGTGCAATAATGAAAGGGCAT | gp3 |
| ssa-miR-130a-5p_1ss9CT | ACTCTTTCTCTGTTGCACTACT | gp3 |
| dre-miR-130b | CAGTGCAATAATGAAAGGGCAT | gp3 |
| ipu-miR-132a | ACCGTGGCTTTAGATTGTTACT | gp3 |
| xtr-miR-132 | TAACAGTCTACAGCCATGGTCG | gp3 |
| ccr-miR-132b_R-1 | ACCATGGCTGTAGACTGTTAC | gp3 |
| ssa-miR-133a-5p_R+1 | AGCTGGTAAAATGGAACCAAAT | gp3 |
| aca-miR-133a_L-1R+1 | TTGGTCCCCTTCAACCAGCTGT | gp3 |
| ccr-miR-133a-5p_R+1 | AGCTGGTAAAATGGAACCAAAT | gp3 |
| ccr-miR-133a-3p | TTGGTCCCCTTCAACCAGCTGT | gp3 |
| oha-miR-133b-3p_R-2 | TTTGGTCCCCTTCAACCAGCT | gp3 |
| bta-miR-133c_L-1R+2 | TTTGGTTCCATTTTACCAGCTT | gp3 |
| rno-miR-133c_L+1R+2 | ACAGCTGGTTGAAGGGGACCAATT | gp3 |
| ccr-miR-135c | TATGGCTTTCTATTCCTATGTGA | gp3 |
| ssa-miR-135b-3-3p_R+1_1ss10CT | ACATAGGGTTCAAAGCCATTGG | gp3 |
| dre-miR-135b-5p_R+1 | TATGGCTTTTTATTCCTATCTGA | gp3 |
| dre-miR-135b-3p_L-1 | TATAGGGATGGAAGCCATGCA | gp3 |
| ssa-miR-137-5p_R+1 | ACGGGTATTCTTGGGTTGATAAT | gp3 |
| tni-miR-137_R+1 | TTATTGCTTGAGAATACGCGTAG | gp3 |
| ola-miR-137_L+3R-2 | TTATTGCTTGAGAATACGCGTAG | gp3 |
| xbo-miR-137_L+1R-1_1ss18AG | TTATTGCTTGAGAATACGCGTT | gp3 |
| aca-miR-138-5p_R+1 | AGCTGGTGTTGTGAATCAGGCCG | gp3 |
| ssa-miR-138-3p_R+1 | GCTATTTCACAACACCAGGGTT | gp3 |
| ola-miR-139_R+2 | TCTACAGTGCATGTGTCTCCAGT | gp3 |
| tgu-miR-139-5p | TCTACAGTGCATGTGTCTCCAGT | gp3 |
| oan-miR-140-5p_R+2 | CAGTGGTTTTACCCTATGGTAG | gp3 |
| oan-miR-140-3p | TACCACAGGGTAGAACCACGGA | gp3 |
| ssa-miR-140-5p | CAGTGGTTTTACCCTATGGTAG | gp3 |
| sha-miR-140_L+1 | TACCACAGGGTAGAACCACGGA | gp3 |
| dre-miR-142a-5p_L+2R-1 | TCCATAAAGTAGAAAGCACTAC | gp3 |
| dre-miR-142a-3p_R-1 | TGTAGTGTTTCCTACTTTATGG | gp3 |
| dre-miR-142a-5p_L+2R-1 | TCCATAAAGTAGAAAGCACTAC | gp3 |
| dre-miR-142a-3p_R-1 | TGTAGTGTTTCCTACTTTATGG | gp3 |
| mmu-miR-142b_R+2_1ss16AG | TCCATAAAGTAGGAAGCACTAC | gp3 |
| ssa-miR-143-5p | GGTGCAGTGCTGCATCTCTGGT | gp3 |
| ccr-miR-143_R+1_1ss20TA | TGAGATGAAGCACTGTAGCAC | gp3 |
| ssa-miR-144-5p_R-1 | GGATATCATCATATACTGTAAGT | gp3 |
| cfa-miR-144_L+1R-3 | CTACAGTATAGATGATGTAC | gp3 |
| gga-miR-144-5p_R+1 | GGATATCATCATATACTGTAAGT | gp3 |
| gga-miR-144-3p_R-2 | CTACAGTATAGATGATGTAC | gp3 |
| pma-miR-144-5p_L-1R-2_1ss10AC | GATATCATCCTATACTGTA | gp3 |
| pma-miR-144-3p_R-2_1ss18GA | TACAGTATATATGATGTACT | gp3 |
| mdo-miR-145-5p_R-1 | GTCCAGTTTTCCCAGGAATCCC | gp3 |
| mdo-miR-145-3p_L+2 | GGATTCCTGGAAATACTGTTCT | gp3 |
| mmu-miR-145b_R-2_1ss18GA | GTCCAGTTTTCCCAGGAAA | gp3 |
| ccr-miR-146a | TGAGAACTGAATTCCATAGATGG | gp3 |
| ssa-miR-146b-3p_R-1_2ss14AG20CT | ATCTATGGACTCAGTTCTTTT | gp3 |
| dre-miR-146b_R+1 | TGAGAACTGAATTCCAAGGGTGT | gp3 |
| hsa-miR-146b-5p_R-3 | TGAGAACTGAATTCCATAG | gp3 |
| dre-miR-148_1ss10TA | TCAGTGCATAACAGAACTTTGT | gp3 |
| dre-miR-150_R+1 | TCTCCCAATCCTTGTACCAGTGT | gp3 |
| bta-miR-151-5p_R+1 | TCGAGGAGCTCACAGTCTAGTA | gp3 |
| bta-miR-151-3p_R+2_1ss10AG | CTAGACTGAGGCTCCTTGAGGAA | gp3 |
| bta-miR-151-5p_R+1 | TCGAGGAGCTCACAGTCTAGTA | gp3 |
| bta-miR-151-3p_R+2_1ss10AG | CTAGACTGAGGCTCCTTGAGGAA | gp3 |
| ssa-miR-153a-2-5p_2ss5CT12AG | TCATTTTTGTGGTTTGCAGCT | gp3 |
| ccr-miR-153b | TTGCATAGTCACAAAAATGAGC | gp3 |
| aca-miR-155-5p_R+1 | TTAATGCTAATCGTGATAGGGGT | gp3 |
| pma-miR-181a-5p | AACATTCAACGCTGTCGGTGAGT | gp3 |
| pma-miR-181a-3p | ACCATCGACCGTTGACTGTACC | gp3 |
| pma-miR-181a-5p | AACATTCAACGCTGTCGGTGAGT | gp3 |
| pma-miR-181a-3p | ACCATCGACCGTTGACTGTACC | gp3 |
| gga-miR-181b-5p_R+1 | AACATTCATTGCTGTCGGTGGGT | gp3 |
| gga-miR-181b-2-3p_L+1 | CTCACTGATCAATGAATGCAAA | gp3 |
| chi-miR-181b-5p | AACATTCATTGCTGTCGGTGGGT | gp3 |
| aca-miR-182-5p_1ss10GA | TTTGGCAATAGTAGAACTCACA | gp3 |
| aca-miR-182-3p | TGGTTCTAGACTTGCCAACT | gp3 |
| aca-miR-182-5p_1ss10GA | TTTGGCAATAGTAGAACTCACA | gp3 |
| aca-miR-182-3p | TGGTTCTAGACTTGCCAACT | gp3 |
| bta-miR-183 | TATGGCACTGGTAGAATTCACTG | gp3 |
| ccr-miR-184 | TGGACGGAGAACTGATAAGGGC | gp3 |
| bta-miR-185 | TGGAGAGAAAGGCAGTTCCTGA | gp3 |
| cgr-miR-185-5p | TGGAGAGAAAGGCAGTTCCTGA | gp3 |
| mdo-miR-186-5p | CAAAGAATTCTCCTTTTGGGCTT | gp3 |
| gga-miR-187-5p_L+1_2ss6AG23AT | GGGCTGCAACACAGGACATGGGT | gp3 |
| ccr-miR-187 | TCGTGTCTTGTGTTGCAGCCAGT | gp3 |
| tni-miR-190_R+1 | TGATATGTTTGATATATTAGGTT | gp3 |
| ssa-miR-190a-3p | ACTATATATCAAACATATTCCT | gp3 |
| oha-miR-191-5p_R-1 | CAACGGAATCCCAAAAGCAGCT | gp3 |
| oha-miR-191-5p_R-1 | CAACGGAATCCCAAAAGCAGCT | gp3 |
| ccr-miR-192 | ATGACCTATGAATTGACAGCC | gp3 |
| ssa-miR-192a-3p_1ss11AC | CCTGTCAGTTCTGTAGGCCACT | gp3 |
| ola-miR-192-5p_L+1R-1 | ATGACCTATGAATTGACAGCC | gp3 |
| ola-miR-192-3p_R-1 | CCTGTCAGTTCTGTAGGCCACT | gp3 |
| gga-miR-193a-5p_R-1 | TGGGTCTTTGCGGGCGAGATG | gp3 |
| gga-miR-193a-3p_R-2 | AACTGGCCTACAAAGTCCCA | gp3 |
| gga-miR-193a-5p_R-1 | TGGGTCTTTGCGGGCGAGATG | gp3 |
| gga-miR-193a-3p_R-2 | AACTGGCCTACAAAGTCCCA | gp3 |
| gga-miR-193b-5p_1ss22AT | CGGGGTTTTGGGGGCGAGATGT | gp3 |
| gga-miR-193b-3p_1ss10AG | AACTGGCCCGCAAAGTCCCGCTTT | gp3 |
| dre-miR-193b-3p | AACTGGCCCGCAAAGTCCCGCT | gp3 |
| aca-miR-194-5p | TGTAACAGCAACTCCATGTGGA | gp3 |
| ssa-miR-194a-3p_R-2_2ss10AC20CT | CCAGTGGAGCTGCTGTTACTT | gp3 |
| aca-miR-194-5p | TGTAACAGCAACTCCATGTGGA | gp3 |
| aca-miR-194-2-3p_R-2_1ss11GT | CCAGTGGAGCTGCTGTTAT | gp3 |
| bta-miR-195_1ss11GT | TAGCAGCACATAAATATTGGCA | gp3 |
| age-miR-196_R+1 | TAGGTAGTTTCATGTTGTTGGGA | gp3 |
| bta-miR-197 | TTCACCACCTTCTCCACCCAGC | gp3 |
| pma-miR-199a-5p_L+3 | ATCCCCAGTGTTCAGACTACCTGTTC | gp3 |
| pma-miR-199b-3p_L+2R-1 | TACAGTAGTCTGCACATTGGTT | gp3 |
| pma-miR-199a-5p_L+3 | ATCCCCAGTGTTCAGACTACCTGTTC | gp3 |
| pma-miR-199b-3p_L+2R-1 | TACAGTAGTCTGCACATTGGTT | gp3 |
| gga-miR-199b_L+1_1ss18TG | TCAGTAGTCTGCACATTGGGT | gp3 |
| ssa-miR-200b-5p | CATCTTACCTGACAGTGCTGGA | gp3 |
| fru-miR-200a_R+1 | TAACACTGTCTGGTAACGATGTT | gp3 |
| ssa-miR-200a-2-5p_2ss9GT10AG | CATCTTACTGGGCAGCATTGGA | gp3 |
| xtr-miR-200b | TAATACTGCCTGGTAATGATGAT | gp3 |
| aca-miR-200b-5p_R+2 | CATCTTACTGGGCAGCATTGGA | gp3 |
| aca-miR-200b-3p_R+1 | TAATACTGCCTGGTAATGATGAT | gp3 |
| bta-miR-200c_R-1_1ss22GA | TAATACTGCCGGGTAATGATGA | gp3 |
| pmi-miR-200-3p_1ss9TC | TAATACTGCCTGGTAATGATGT | gp3 |
| dre-miR-202-5p_R-1 | TTCCTATGCATATACCTCTTT | gp3 |
| dre-miR-202-3p_L+2R-2 | AAAGAGGCATAGGGCATGGGAA | gp3 |
| dre-miR-202-5p_R-1 | TTCCTATGCATATACCTCTTT | gp3 |
| dre-miR-202-3p_L+2R-2 | AAAGAGGCATAGGGCATGGGAA | gp3 |
| oha-miR-202-5p_R-1 | TTCCTATGCATATACTTCTTT | gp3 |
| ccr-miR-203b-5p_R-1 | AGTGGTTCTTAACAGTTCAACA | gp3 |
| ccr-miR-203a | GTGAAATGTTTAGGACCACTTG | gp3 |
| ccr-miR-203b-5p_R-1 | AGTGGTTCTTAACAGTTCAACA | gp3 |
| ccr-miR-203a | GTGAAATGTTTAGGACCACTTG | gp3 |
| ipu-miR-203c | TTGAACTGTTAAGAACCACTGC | gp3 |
| oha-miR-204-5p | TTCCCTTTGTCATCCTATGCCT | gp3 |
| aca-miR-204a-5p | TTCCCTTTGTCATCCTATGCCT | gp3 |
| oan-miR-205-5p_R+2 | TCCTTCATTCCACCGGAGTCTGT | gp3 |
| ssa-miR-205b-2-3p_R-2_1ss20GA | GATTTCAGTGGTGTGAAGAA | gp3 |
| ssa-miR-205b-5p_R+1 | TCCTTCATTCCACCGGAGTCTGT | gp3 |
| ssa-miR-205b-2-3p_1ss20GA | GATTTCAGTGGTGTGAAGAATA | gp3 |
| ssa-miR-206-5p_R+1 | ACATGCTTCCTTATATCCCCAT | gp3 |
| ola-miR-206_R+1 | TGGAATGTAAGGAAGTGTGTGG | gp3 |
| ola-miR-210-5p_R+2_1ss20TC | AGCCACTGACTAACGCACACTG | gp3 |
| ola-miR-210-3p_L-1R+3 | CTGTGCGTGTGACAGCGGCT | gp3 |
| cgr-miR-210-3p_R-2 | CTGTGCGTGTGACAGCGGCT | gp3 |
| ipu-miR-212 | ACCTTGGCTCTAGACTGCTTACT | gp3 |
| ssa-miR-212a-3p | TAACAGTCTACAGTCATGGCT | gp3 |
| ssa-miR-212b-5p | ACCTTGGCTCTAGACTGCTTACT | gp3 |
| xtr-miR-212 | TAACAGTCTACAGTCATGGCT | gp3 |
| hsa-miR-212-5p | ACCTTGGCTCTAGACTGCTTACT | gp3 |
| aca-miR-214-5p | TGCCTGTCTACACTTGCTGTGC | gp3 |
| aca-miR-214-3p | ACAGCAGGCACAGACAGGCAGT | gp3 |
| csa-miR-216b | TAATCTCTGCAGGCAACTGTGA | gp3 |
| pma-miR-216b_R-1_1ss1AT | TAATCTCTGCAGGCAACTGTGG | gp3 |
| bta-miR-217_R-1 | TACTGCATCAGGAACTGATTGGA | gp3 |
| ccr-miR-218a_R+1 | TTGTGCTTGATCTAACCATGTGT | gp3 |
| ssa-miR-218-3p_L+2R-3 | TCACATGGTTCCGTCAAGCACC | gp3 |
| dre-miR-219-5p_R-1 | TGATTGTCCAAACGCAATTCT | gp3 |
| ssa-miR-219a-3p_1ss11AC | AGAATTGTGCCTGGACATCTGT | gp3 |
| dre-miR-219-5p_R-1 | TGATTGTCCAAACGCAATTCT | gp3 |
| ssa-miR-219b-5p_R-2 | TGATTGTCCAAACGCAATTCT | gp3 |
| ipu-miR-219a | AGAATTGTGCCTGGACATCTGT | gp3 |
| hsa-miR-219a-5p | TGATTGTCCAAACGCAATTCT | gp3 |
| hsa-miR-219b-3p_L+1R-3_1ss21CT | AAGAATTGCGTTTGGACAATT | gp3 |
| gga-miR-219b_L-3R+2 | AAGAATTGCGTTTGGACAATT | gp3 |
| ssa-miR-221-3p_R-1_1ss5AG | ACCTGGCATACAATGTAGATTT | gp3 |
| bta-miR-221 | AGCTACATTGTCTGCTGGGTTT | gp3 |
| cgr-miR-221-5p_R-4 | ACCTGGCATACAATGTAGATTT | gp3 |
| cgr-miR-221-3p_R-1 | AGCTACATTGTCTGCTGGGTTT | gp3 |
| dre-miR-222a-5p | TGCTCAGTAGTCAGTGTAGATCC | gp3 |
| dre-miR-222a-3p_R-1 | AGCTACATCTGGCTACTGGGTCT | gp3 |
| dre-miR-222a-5p | TGCTCAGTAGTCAGTGTAGATCC | gp3 |
| dre-miR-222a-3p_R-1 | AGCTACATCTGGCTACTGGGTCT | gp3 |
| gga-miR-222b-5p_R-7 | TGCTCAGTAGTCAGTGTAG | gp3 |
| gga-miR-222b-3p_R-1 | AGCTACATCTGATTACTGGGTCA | gp3 |
| bta-miR-223 | TGTCAGTTTGTCAAATACCCCA | gp3 |
| ipu-miR-223_1ss20GT | GAGTATTTGACAGACTGTGT | gp3 |
| bta-miR-224_R+3 | CAAGTCACTAGTGGTTCCGTTTAGTT | gp3 |
| sko-miR-252a_R-1 | CTAAGTACTAGTGCCGCAGGAG | gp3 |
| lva-miR-252a-5p_R-4_1ss18AT | CTAAGTACTAGTGCCGTT | gp3 |
| sko-miR-278_1ss17TC | TCGGTGGGACTTTCGTCCGTTT | gp3 |
| odi-miR-281_1ss21GT | TGTCATGGAATTGCTCTCTTT | gp3 |
| hsa-miR-296-5p | AGGGCCCCCCCTCAATCCTGT | gp3 |
| dre-miR-301c-5p_R+1_1ss4CT | GCTTTGACGATGTTGCACTACT | gp3 |
| dre-miR-301c-3p_R+1 | CAGTGCAATAGTATTGTCATAGC | gp3 |
| dre-miR-301c-5p_R+1_1ss4CT | GCTTTGACGATGTTGCACTACT | gp3 |
| ssa-miR-301a-3p | CAGTGCAATAGTATTGTCATAGC | gp3 |
| sko-miR-315_R+2 | TTTTGATTGTTGCTCAGAAAGCC | gp3 |
| bta-miR-320a | AAAAGCTGGGTTGAGAGGGCGA | gp3 |
| bta-miR-324_R-1 | CGCATCCCCTAGGGCATTGGTG | gp3 |
| cgr-miR-324-5p | CGCATCCCCTAGGGCATTGGTG | gp3 |
| bta-miR-328 | CTGGCCCTCTCTGCCCTTCCGT | gp3 |
| mmu-miR-329-5p | AGAGGTTTTCTGGGTCTCTGTT | gp3 |
| hsa-miR-331-5p_R-1 | CTAGGTATGGTCCCAGGGATC | gp3 |
| bta-miR-335 | TCAAGAGCAATAACGAAAAATGT | gp3 |
| ssa-miR-338a-5p_1ss20AC | AACAATATCCTGATGCTGCCTGAGT | gp3 |
| dre-miR-338_R-1 | TCCAGCATCAGTGATTTTGTT | gp3 |
| ppy-miR-338-3p_R-1 | TCCAGCATCAGTGATTTTGTT | gp3 |
| hsa-miR-339-5p_R-4 | TCCCTGTCCTCCAGGAGCT | gp3 |
| hsa-miR-339-3p_L-2_1ss3AT | TGCGCCTCGACGACAGAGCCG | gp3 |
| hsa-miR-339-5p_R-4 | TCCCTGTCCTCCAGGAGCT | gp3 |
| ggo-miR-339_L-2_1ss3AT | TGCGCCTCGACGACAGAGCCG | gp3 |
| cfa-miR-340 | TTATAAAGCAATGAGACTGATT | gp3 |
| hsa-miR-340-5p | TTATAAAGCAATGAGACTGATT | gp3 |
| cgr-miR-342-3p_R-1 | TCTCACACAGAAATCGCACCCGT | gp3 |
| chi-miR-361-5p | TTATCAGAATCTCCAGGGGTAC | gp3 |
| chi-miR-361-3p_R+2 | TCCCCCAGGTGTGATTCTGATTTG | gp3 |
| efu-miR-361 | TTATCAGAATCTCCAGGGGTAC | gp3 |
| aca-miR-363-5p | GTGGATCACGATGCAATTTTGA | gp3 |
| aca-miR-363-3p | AATTGCACGGTATCCATCTGT | gp3 |
| hsa-miR-363-3p_R-1 | AATTGCACGGTATCCATCTGT | gp3 |
| ssa-miR-365-5p_R+3 | AGGGACTTTTAGGGGCAGCTGTG | gp3 |
| dre-miR-365_1ss11AG | TAATGCCCCTGAAAATCCTTAT | gp3 |
| oha-miR-365a-2-5p_1ss10CT | AGGGACTTTTAGGGGCAGCTGTG | gp3 |
| oha-miR-365a-3p_1ss11AG | TAATGCCCCTGAAAATCCTTAT | gp3 |
| ssc-miR-369 | AATAATACATGGTTGATCTTT | gp3 |
| bta-miR-374b_R-1 | ATATAATACAACCTGCTAAGT | gp3 |
| hsa-miR-374a-5p | TTATAATACAACCTGATAAGTG | gp3 |
| tni-miR-375_R-1 | TTTGTTCGTTCGGCTCGCGTT | gp3 |
| bta-miR-378 | ACTGGACTTGGAGTCAGAAGGC | gp3 |
| bta-miR-379 | TGGTAGACTATGGAACGTAGG | gp3 |
| aca-miR-383-5p_R+1 | AGATCAGAAGGTGATTGTGGCT | gp3 |
| aca-miR-383-5p_R+1 | AGATCAGAAGGTGATTGTGGCT | gp3 |
| bta-miR-410 | AATATAACACAGATGGCCTGT | gp3 |
| chi-miR-421-3p_1ss23CT | ATCAACAGACATTAATTGGGCGT | gp3 |
| mmu-miR-421-3p_1ss23CT | ATCAACAGACATTAATTGGGCGT | gp3 |
| bta-miR-423-5p | TGAGGGGCAGAGAGCGAGACTTT | gp3 |
| bta-miR-423-3p_L-1 | AGCTCGGTCTGAGGCCCCTCAGT | gp3 |
| bta-miR-423-5p | TGAGGGGCAGAGAGCGAGACTTT | gp3 |
| bta-miR-423-3p_L-1 | AGCTCGGTCTGAGGCCCCTCAGT | gp3 |
| chi-miR-424-5p | CAGCAGCAATTCATGTTTTGA | gp3 |
| tgu-miR-425-5p_R+3 | AATGACACGATCACTCCCGCTGAGC | gp3 |
| tgu-miR-425-3p_R+1_1ss20TG | CATCGGGGATGTCGTGTCTGTC | gp3 |
| bta-miR-425-5p_L+1R-1 | AATGACACGATCACTCCCGTTG | gp3 |
| ssa-miR-429-5p_R+1 | GTCTTACCAGACATGGTTAGA | gp3 |
| xtr-miR-429 | TAATACTGTCTGGTAATGCCGT | gp3 |
| ccr-miR-430 | TAAGTGCTATTTGTTGGGGTAG | gp3 |
| dre-miR-430a-3p | TAAGTGCTATTTGTTGGGGTAG | gp3 |
| bta-miR-431_R-3 | TGTCTTGCAGGCCGTCATGC | gp3 |
| mmu-miR-450b-3p_L-1R-1_1ss3TG | TGGGGAACATTTTGCATGCA | gp3 |
| aca-miR-451-3p_L+2R+1_1ss18TC | TTTAGTAATGGTAAGGGCTCTT | gp3 |
| pma-miR-451_R-4_1ss16TC | AAACCGTTACCATTACTGTA | gp3 |
| ssa-miR-454-5p | ACCCTATCAATATTGCCTCTGC | gp3 |
| aca-miR-454-3p | TAGTGCAATATTGCTTATAGGGT | gp3 |
| tni-miR-455 | TATGTGCCCTTGGACTACATCG | gp3 |
| ssa-miR-455-3p | GCAGTCCATGGGCATATACACT | gp3 |
| dre-miR-455-5p | TATGTGCCCTTGGACTACATCG | gp3 |
| dre-miR-455-3p_L-2R+1 | GCAGTCCATGGGCATATACACT | gp3 |
| gga-miR-456-5p_L+1R-4_1ss13TC | GCAGGCATCTTCCCAGCCTACA | gp3 |
| gga-miR-456-3p_1ss22AT | CAGGCTGGTTAGATGGTTGTCT | gp3 |
| ccr-miR-457a_L+1R+1 | TAGCAGCACATCAATATTGGCA | gp3 |
| dre-miR-458-5p_R-2 | AGCGCCATTTACAGAGCTAT | gp3 |
| dre-miR-458-3p | ATAGCTCTTTGAATGGTACTGC | gp3 |
| ccr-miR-459-5p_L+2R-1 | TCAGTAACAAGGATTCATCCTGT | gp3 |
| ccr-miR-459-3p_R+1_1ss5GA | CAGGAAATCTCTGTTACTGGGG | gp3 |
| ssa-miR-460-5p_1ss15AG | CCTGCATTGTACACGCTGTGCG | gp3 |
| ssa-miR-460-3p | CACAGCGCATACAATGTGGATG | gp3 |
| dre-miR-462_R+1 | TAACGGAACCCATAATGCAGCTG | gp3 |
| mmu-miR-466m-5p_2ss14AT22AG | TGTGTGCATGTGCTTGTGTGTGT | gp3 |
| mmu-miR-466i-5p_L-2 | TGTGTGTGTGTGTGTGTG | gp3 |
| mmu-miR-466i-5p_L-2 | TGTGTGTGTGTGTGTGTG | gp3 |
| rno-miR-466b-4-3p_L+2_1ss18TC | ACATATACATACACACACACACA | gp3 |
| mmu-miR-466g_L-3_1ss6GC | CACACACATGCACACACA | gp3 |
| mmu-miR-467g_L+1 | ATATACATACACACACATATAT | gp3 |
| bta-miR-484 | TCAGGCTCAGTCCCCTCCCGAT | gp3 |
| mmu-miR-486a-5p | TCCTGTACTGAGCTGCCCCGAG | gp3 |
| mmu-miR-486b-3p_R+1 | CGGGGCAGCTCAGTACAGGAT | gp3 |
| mmu-miR-486a-5p | TCCTGTACTGAGCTGCCCCGAG | gp3 |
| mmu-miR-486b-3p_R+1 | CGGGGCAGCTCAGTACAGGAT | gp3 |
| aca-miR-489-5p | TGGTCGTATGTATGACGTCATT | gp3 |
| aca-miR-489-3p_L-1R+1 | TGACATCATATGTACGGCTGCT | gp3 |
| aca-miR-490-3p | CAACCTGGAGGACTCCATGCTGT | gp3 |
| bta-miR-494_R+2 | TGAAACATACACGGGAAACCTCTA | gp3 |
| hsa-miR-494-3p_R+2 | TGAAACATACACGGGAAACCTCTA | gp3 |
| aca-miR-499-5p_R-1 | TTAAGACTTGCAGTGATGTTT | gp3 |
| aca-miR-499-3p_R-1 | AACATCACTTTAAGTCTGTGC | gp3 |
| ssa-miR-499b-5p_R-1 | TTAAGACTTGCAGTGATGTTT | gp3 |
| ola-miR-499 | AACATCACTTTAAGTCTGTGC | gp3 |
| cgr-miR-499-5p_R-1 | TTAAGACTTGCAGTGATGTTT | gp3 |
| cgr-miR-500_R-1 | AATGCACCTGGGCAAGGGTT | gp3 |
| bta-miR-503-5p_R+3 | TAGCAGCGGGAACAGTACTGCAG | gp3 |
| bta-miR-503-5p_R+3 | TAGCAGCGGGAACAGTACTGCAG | gp3 |
| bta-miR-532_R-2 | CATGCCTTGAGTGTAGGACC | gp3 |
| cgr-miR-532-5p_R-2 | CATGCCTTGAGTGTAGGACC | gp3 |
| mmu-miR-541-5p_R-5 | AAGGGATTCTGATGTTGGTC | gp3 |
| hsa-miR-545-3p_1ss22CT | TCAGCAAACATTTATTGTGTGT | gp3 |
| ccr-miR-551_R-1 | GCGACCCATCCTTGGTTTCT | gp3 |
| ggo-miR-574 | CACGCTCATGCACACACCCACA | gp3 |
| ggo-miR-584 | TTATGGTTTGCCTGGGACTGA | gp3 |
| cfa-miR-590_L+1 | TTAATTTTATGTATAAGCTAGT | gp3 |
| eca-miR-590-3p_L+1 | TTAATTTTATGTATAAGCTAGT | gp3 |
| bta-miR-652_R+1 | AATGGCGCCACTAGGGTTGTGT | gp3 |
| cgr-miR-652-3p_R+1 | AATGGCGCCACTAGGGTTGTGT | gp3 |
| bta-miR-669_L-3_1ss5GT | GTGTGTGTGCATGTGCGTG | gp3 |
| bta-miR-708_R+1 | AAGGAGCTTACAATCTAGCTGGGA | gp3 |
| mmu-miR-721_R-3_1ss18GC | CAGTGCAATTAAAAGGGC | gp3 |
| ssa-miR-722-5p_L-1R+1 | TTTGAAACGTTTTAGCCAAAA | gp3 |
| ccr-miR-722_L-2R+3 | TTTTGCAGAAACGTTTCAGATT | gp3 |
| ssa-miR-723-3p | AGACATCAGATAAATCTGTGCT | gp3 |
| ssa-miR-724-5p | TTAAAGGGAATTTGCGACTGTT | gp3 |
| ssa-miR-724-3p | CAGCCACACCTTCCTTTTAAGA | gp3 |
| ssa-miR-725-3p | TTCAGTCATTGTTTCTGGTAGT | gp3 |
| aca-miR-727-5p_R+2 | TCAGTCTTCAATTCCTCCCAGC | gp3 |
| aca-miR-727-3p_L-1R+3 | TTGAGGCGAGTTGAAGACTAAA | gp3 |
| ccr-miR-730_R+1 | TCCTCATTGTGCATGCTGTGTGT | gp3 |
| ssa-miR-730a-3p | CACAGCGCCTGCAATGTGGAGG | gp3 |
| ola-miR-731_R+5 | AATGACACGTTTTCTCCCGGATT | gp3 |
| ccr-miR-734_R+1_1ss1TC | CAAATGCTGCAGAATCGTACCGT | gp3 |
| ssa-miR-734-5p | GAACTATTCTGCAACATTTGTT | gp3 |
| dre-miR-737-5p_R+1_1ss2TG | GGTTTTTTAGGTTTTGATTTTT | gp3 |
| dre-miR-737-3p | AATCAAAACCTAAAGAAAATA | gp3 |
| bta-miR-744_R-1 | TGCGGGGCTAGGGCTAACAGC | gp3 |
| bta-miR-877_1ss20GA | GTAGAGGAGATGGCGCAGGA | gp3 |
| pmi-miR-981-3p_R+1_1ss21AC | TTCGTTGTCGACGAAACCTGCA | gp3 |
| cgr-miR-1260 | ATCCCACCGCTGCCACCA | gp3 |
| cfa-miR-1271 | CTTGGCACCTAGTAAGCACT | gp3 |
| hsa-miR-1277-5p_L-3R+2 | TATATATATATATGTACGTATGT | gp3 |
| hsa-miR-1285-3p_R-1 | TCTGGGCAACAAAGTGAGACC | gp3 |
| bta-miR-1296_R+1 | TTAGGGCCCTGGCTCCATCTCCT | gp3 |
| cgr-miR-1306-3p_L+1 | GACGTTGGCTCTGGTGGTGATG | gp3 |
| hsa-miR-1307-5p | TCGACCGGACCTCGACCGGCT | gp3 |
| chi-miR-1307-5p_R+4 | TCGACCGGACCTCGACCGGCT | gp3 |
| oan-miR-1352_R+1_1ss15TG | TGGAGTGTGACATTGGTGA | gp3 |
| oan-miR-1357_L+3_1ss21CT | AAGATTATGAGATCTGAGGGTC | gp3 |
| oan-miR-1386_L+3 | CGGCTCCTGGCTGGCTCGCCA | gp3 |
| oan-miR-1388-5p | AGGACTGTCTAACCTGAGAATG | gp3 |
| oan-miR-1388-3p | ATCTCAGGTTCGTCAGCCCATG | gp3 |
| oan-miR-1388-5p | AGGACTGTCTAACCTGAGAATG | gp3 |
| oan-miR-1388-3p | ATCTCAGGTTCGTCAGCCCATG | gp3 |
| gga-miR-1451-5p_R+1 | TCGCACAGGAGCAAGTTACCGCT | gp3 |
| gga-miR-1552-5p_R+1_1ss10GA | TTAGTGCGCAGTAAGCTAGGGTGT | gp3 |
| gga-miR-1559-5p | TTCGATGCTTGTATGCTACTCC | gp3 |
| gga-miR-1565_L-2R-3_1ss10TG | AGGGTCGGGCCTGGTTTT | gp3 |
| gga-miR-1662 | TTGACATCATCATACTTGGGAT | gp3 |
| tgu-miR-1677-3p | TTGACTTCAATAGGAGCAGGATT | gp3 |
| gga-miR-1730-5p_L-4R-1_1ss14TA | TAACAGAGGAGGAACCTT | gp3 |
| dre-miR-1788-5p | GGCTTGTTTTAAGTTGCCTGCG | gp3 |
| dre-miR-1788-3p_R+1 | CAGGCAGCTAAAGCAAGTCTGT | gp3 |
| dre-miR-1788-5p | GGCTTGTTTTAAGTTGCCTGCG | gp3 |
| dre-miR-1788-3p_R+1 | CAGGCAGCTAAAGCAAGTCTGT | gp3 |
| bta-miR-1814c_R-1_1ss15TA | GTTTTGTTTGGGTTAGTTT | gp3 |
| bta-miR-1839_L-1R-1 | AGGTAGATAGAACAGGTCTTGT | gp3 |
| cgr-miR-1973_1ss10AG | TGACCGTGCGAAGGTAGC | gp3 |
| gga-miR-2131-5p | ATGCAGAAGTGCACGGAAACAGCT | gp3 |
| ssa-miR-2187-5p_L-1 | TTAATTAGTATAGCCTGTATT | gp3 |
| ssa-miR-2187-3p_R-1 | TTACAGGCTATGCTAATCTATG | gp3 |
| dre-miR-2188-5p | AAGGTCCAACCTCACATGTCC | gp3 |
| dre-miR-2188-3p_L+1 | GCTGTGTGAGGTTAGACCTATC | gp3 |
| bta-miR-2354_R-3_1ss6GA | TAGTAAGTTGTGTTGTTT | gp3 |
| bta-miR-2478_L-2 | ATCCCACTTCTGACACCA | gp3 |
| bta-miR-2481_1ss4GA | TACATTGTCTGCTGGGTTTT | gp3 |
| bta-miR-2887_L+2R+3 | GTCGGGACCGGGGTCCGGTGCGGAG | gp3 |
| tgu-miR-2954-5p_L+1 | TGCTGAGAGGGCTTGGGGAGAGGA | gp3 |
| tgu-miR-2954-3p | CATCCCCATTCCACTCCTAGCAG | gp3 |
| tgu-miR-2957 | TCGGAGTGTCACAGAACTTTGC | gp3 |
| tgu-miR-2970-5p_R+1 | GACAGTCAGCAGTTGGTCTGGT | gp3 |
| tgu-miR-2976_R-3_1ss9GC | GCGGAGCGCAGCGGAGCG | gp3 |
| tgu-miR-2995_L-1_1ss2GC | CGCACTGTTCGTAACCTGTT | gp3 |
| rno-miR-3084d_L-3_1ss19GT | TGCCAGTCCCCTTCATACA | gp3 |
| rno-miR-3084d_L-3_1ss19GT | TGCCAGTCCCCTTCATACA | gp3 |
| hsa-miR-3120-5p_L-1R+2 | CTGTCTGTGCCTGCTGTACAGG | gp3 |
| hsa-miR-3120-3p_R+2 | CACAGCAAGTGTAGACAGGCAGT | gp3 |
| hsa-miR-3120-5p_L-1R+2 | CTGTCTGTGCCTGCTGTACAGG | gp3 |
| hsa-miR-3120-3p_R+2 | CACAGCAAGTGTAGACAGGCAGT | gp3 |
| hsa-miR-3591-5p_L-1 | TTAGTGTGATAATGGCGTTTGA | gp3 |
| hsa-miR-3591-3p_1ss22CT | AAACACCATTGTCACACTCCAT | gp3 |
| aja-miR-3596_L+3_1ss8AT | CAACTATTCAATCTACTACCTCA | gp3 |
| mmu-miR-3968_L-3_1ss14AT | ATCCCACTCCTGACACCA | gp3 |
| cin-miR-4185-3p_L-2R-2_1ss19GT | TATTCATACTGTCTGATC | gp3 |
| hsa-miR-4286_R+1 | ACCCCACTCCTGGTACCA | gp3 |
| hsa-miR-4443_L+1 | CTTGGAGGCGTGGGTTTT | gp3 |
| hsa-miR-4448_1ss6CG | GGCTCGTTGGTCTAGGGGTA | gp3 |
| hsa-miR-4454_L+1_1ss9AG | CGGATCCGGGTCACGGCACCA | gp3 |
| hsa-miR-4492_L+1 | CGGGGCTGGGCGCGCGCC | gp3 |
| hsa-miR-4508_R+1 | GCGGGGCTGGGCGCGCGC | gp3 |
| hsa-miR-4697-3p_L-2R-3_1ss5AT | TCTGTGACTCCTGCCCCTT | gp3 |
| hsa-miR-4732-5p_R-2 | TGTAGAGCAGGGAGCAGGAAG | gp3 |
| bbe-miR-4864-3p_L-3R-1_1ss16CA | CAGACCTGATCTACCTGC | gp3 |
| mmu-miR-5126_L-4_1ss19GC | GCGGGGCCGGGGGCCGGG | gp3 |
| hsa-miR-6089_L-5R-1_1ss16GT | CCGGGGTGGGTCGGGGCG | gp3 |
| mml-miR-6134_R+3_1ss18GT | TGAGGTAGTAGGATGTATAGTT | gp3 |
| mmu-miR-6238_L-1R-3_1ss12TC | TATTAGTCAGCGGAGGAA | gp3 |
| mmu-miR-6412_R-2_1ss15AT | TCGAAACCATCCTCTGCTAC | gp3 |
| bta-miR-6526 | TCCTGTGCCTCGAATGGGTATG | gp3 |
| bta-miR-6529a | GAGAGATCAGAGGCGCAGAGT | gp3 |
| mml-miR-6529-5p | GAGAGATCAGAGGCGCAGAGT | gp3 |
| gga-miR-6595-5p_L-5R-2_1ss17TG | TGATGAATCCTGTCTGAT | gp3 |
| hsa-miR-6726-3p_L+1R-2 | GCTCGCCCTGTCTCCCGCT | gp3 |
| hsa-miR-6818-5p_L+1R+1 | TTTGTGTGAGTACAGAGAGCATCT | gp3 |
| hsa-miR-6867-5p_L-2R-3_1ss13GA | TGTGTGTAGAAGAAGAAG | gp3 |
| hsa-miR-6873-3p_R-3_1ss1TC | CTCTCTCTGTCTTTCTCTCT | gp3 |
| mmu-miR-6937-5p_L-4R-2_1ss20TG | TGTAAGGGCTGGGTCGGT | gp3 |
| ssa-miR-7132b-5p_1ss10AT | GACTTGGTCTAAGCTCCTCAGC | gp3 |
| ccr-miR-7132_1ss11AT | TGAGGAGTTTTGAGCAAGTAA | gp3 |
| ssc-miR-7134-5p | ATGTCCGCGGGTTCCCTATCC | gp3 |
| ssc-miR-7134-3p | ATGCGGAACCTGCGGATACGG | gp3 |
| dre-miR-7147 | TGTACCATGCTGGTAGCCAGT | gp3 |
| mmu-miR-7233-5p_L-1_1ss19GT | GTTAGGGACAGATAGATT | gp3 |
| ipu-miR-7547_R-2 | AGCGGCGTCAGAAGCGATGG | gp3 |
| ipu-miR-7548_1ss12TG | AGCCGCGGCTGGAGGAGC | gp3 |
| ipu-miR-7550 | ATCCGGCTCGAAGGACCA | gp3 |
| ssa-miR-7552a-5p | TTACAATTAAAGGATATTTCTT | gp3 |
| ssa-miR-7552a-5p | TTACAATTAAAGGATATTTCTT | gp3 |
| ipu-miR-7553 | TGACGTCATTAGCGACCCGACC | gp3 |
| ipu-miR-7562_1ss11CG | CACACACACTGATGAACACACA | gp3 |
| ipu-miR-7573_L-1R-1_1ss5TC | GGCCGAGCCTGATGGCACTGA | gp3 |
| hsa-miR-7977_1ss6AG | TTCCCGGCCAACGCACCA | gp3 |
| ssa-miR-8160-5p_R-2 | AGAATAATGCCAGCAGTCGG | gp3 |
| cfa-miR-8873a_L-3R-2_1ss11AG | TGAAGGCGGTGGGGTGTA | gp3 |
| efu-miR-9226_L-4_1ss22GA | GTCCCTGTTCGGGCGCCA | gp3 |
| ssc-miR-9804-5p_L-1R-2_1ss16TA | CCAGGATCAGAGGAAGGA | gp3 |
| mdo-let-7a-5p | TGAGGTAGTAGGTTGTATAGTT | gp3 |
| ssa-let-7g-3p_2ss8GA22CT | CTATACAATCTACTGTCTTTCT | gp3 |
| gga-let-7a-5p | TGAGGTAGTAGGTTGTATAGTT | gp3 |
| gga-let-7a-3p | CTATACAATCTACTGTCTTTCC | gp3 |
| gga-let-7a-5p | TGAGGTAGTAGGTTGTATAGTT | gp3 |
| tni-let-7i | TGAGGTAGTAGTTTGTGCTGTT | gp3 |
| ssa-let-7i-2-3p_2ss9GT22CT | CTGCGCAATCTACTGCCTTGCT | gp3 |
| rno-let-7b-5p | TGAGGTAGTAGGTTGTGTGGTT | gp3 |
| rno-let-7b-3p | CTATACAACCTACTGCCTTCCC | gp3 |
| hsa-let-7d-5p_1ss16CT | AGAGGTAGTAGGTTGTATAGTT | gp3 |
| cfa-let-7d_R-3 | CTATACGACCTGCTGCCTTTCT | gp3 |
| aca-let-7i-5p | TGAGGTAGTAGTTTGTGCTGTT | gp3 |
| aca-let-7i-3p_R-1_1ss9GT | CTGCGCAATCTACTGCCTTGCT | gp3 |
| ccr-let-7j | TGAGGTAGTTGTTTGTACAGTT | gp3 |
| pma-let-7b-5p_1ss15TG | TGAGGTAGTAGGTTGTGTAGTT | gp3 |
| odi-let-7d_R-2_1ss19CA | TGAGGTAGTGGGTTGTATAG | gp3 |
| mmu-let-7k_R+1_1ss9GT | TGAGGTAGTAGGTTGTGTGC | gp3 |
| pmi-let-7-5p_1ss10CA | TGAGGTAGTAGGTTGTAAAGA | gp3 |
| PC-5p-101341_6 | TATTTTACTGTGCGCGAGGA | gp4 |
| PC-3p-148475_3 | TTCGGCAGGATTAAGCGACA | gp4 |
| PC-5p-101341_6 | TATTTTACTGTGCGCGAGGA | gp4 |
| PC-3p-148475_3 | TTCGGCAGGATTAAGCGACA | gp4 |
| PC-5p-71408_13 | TGACTACCGTCCCATAGCACT | gp4 |
| PC-3p-56629_24 | TGCTTTGAGAGGCTAGTCATG | gp4 |
| PC-3p-56629_24 | TGCTTTGAGAGGCTAGTCATG | gp4 |
| PC-5p-71408_13 | TGACTACCGTCCCATAGCACT | gp4 |
| PC-5p-97332_7 | TCGGCTCTTGCGGCGCTGAGT | gp4 |
| PC-3p-199362_2 | CTGAACTCGACAAAACTGCT | gp4 |
| PC-5p-97332_7 | TCGGCTCTTGCGGCGCTGAGT | gp4 |
| PC-3p-199362_2 | CTGAACTCGACAAAACTGCT | gp4 |
| PC-5p-22531_124 | TGCCCCATGAGAACATTTGTAA | gp4 |
| PC-3p-70248_14 | TCAGAAGTAATCATGGGGGAGC | gp4 |
| PC-5p-22531_124 | TGCCCCATGAGAACATTTGTAA | gp4 |
| PC-3p-70248_14 | TCAGAAGTAATCATGGGGGAGC | gp4 |
| PC-5p-45012_40 | AGCCCCATTCACTTCCATAGT | gp4 |
| PC-3p-41121_48 | TGTGGAGGTGAATGGGGCTCAT | gp4 |
| PC-5p-45012_40 | AGCCCCATTCACTTCCATAGT | gp4 |
| PC-3p-41121_48 | TGTGGAGGTGAATGGGGCTCAT | gp4 |
| PC-5p-78795_11 | ACTTGGCCATCGGATCTCTCT | gp4 |
| PC-5p-78795_11 | ACTTGGCCATCGGATCTCTCT | gp4 |
| PC-5p-254699_2 | TGATAAACTCTGAAAACCA | gp4 |
| PC-3p-280604_2 | GATAAACTCTGAAAACCA | gp4 |
| PC-5p-36494_60 | CGGATTGGTGGTGTTTGTGA | gp4 |
| PC-3p-2180_946 | CCCGAACTCCACCCATCTTTGG | gp4 |
| PC-5p-48126_34 | CATGACTAGCCTCTCAAAGCAC | gp4 |
| PC-5p-48126_34 | CATGACTAGCCTCTCAAAGCAC | gp4 |
| PC-5p-67856_15 | GCTTTGGCTCTGCTGTAAACTGC | gp4 |
| PC-3p-203937_2 | TCACTCGTGTGTCTGCCTTCAAT | gp4 |
| PC-5p-95740_7 | ACCTGTTGCTTGCTAGTGTGA | gp4 |
| PC-3p-106798_5 | TACCCTAGTGTAGCAATGGTGA | gp4 |
| PC-5p-147477_3 | TGTGATGCTGAATCTCTCTCTAT | gp4 |
| PC-3p-88112_8 | AGATGAGACTCAGCATCACAGA | gp4 |
| PC-5p-81440_10 | TCATTGGACGAGACGACTGTCA | gp4 |
| PC-3p-75877_11 | GAAGGGTGTCTCGTCCGATGAT | gp4 |
| PC-5p-117918_5 | GAGCCCTGAAAGTTAACAAAT | gp4 |
| PC-3p-46255_37 | TTTGGTTAGGTTTCAGGGCTAC | gp4 |
| PC-5p-126353_4 | TGAAGGGTGGTGTAAATGATGA | gp4 |
| PC-3p-165932_3 | TACATCACTGCAGCATTCCTTG | gp4 |
| PC-5p-115727_5 | ATTCGGTCTGCGCTACCTGGGA | gp4 |
| PC-3p-248952_2 | GAACGGAAATCCGAGCACACGAAAA | gp4 |
| PC-5p-94006_7 | CTTTATATTGAATTATTTGGTG | gp4 |
| PC-3p-46781_37 | TCCAAAATAATTCTCTGTAATC | gp4 |
| PC-5p-132635_4 | AGGTTGGAGACAGGCTGGA | gp4 |
| PC-3p-245481_2 | AAACTGGGTGAATGCTATCAAC | gp4 |
| PC-5p-79649_10 | ATGACTACCGTCCCATAGCACTT | gp4 |
| PC-3p-36625_60 | TGCTTTGAGAGGCTAGTCATGA | gp4 |
| PC-5p-101262_6 | ACTACTTCGGCCTGATCACCTT | gp4 |
| PC-3p-123676_4 | TGTCTGGAACCCTGAACTCCTCCAAG | gp4 |
| PC-5p-46219_38 | ACAGGTGTCTTGTCCGATGAT | gp4 |
| PC-5p-46219_38 | ACAGGTGTCTTGTCCGATGAT | gp4 |
| PC-5p-118506_4 | AACCTCATCTACCCTCCTCTGTT | gp4 |
| PC-3p-150621_3 | GTGGTGGTAGGTGGGCTC | gp4 |
| PC-5p-83983_9 | GCTTATTGTGAGTCCCGGGTA | gp4 |
| PC-5p-83983_9 | GCTTATTGTGAGTCCCGGGTA | gp4 |
| PC-5p-482_5789 | AGGAAAGAGTGGACAATAGCA | gp4 |
| PC-3p-33291_71 | CTATTGTCCACTCTGTCCTC | gp4 |
| PC-5p-166872_3 | CTGTGTAGGATAGGTGGGA | gp4 |
| PC-3p-93657_7 | GTAGGATAGGTGGGAGGC | gp4 |
| PC-5p-27588_95 | ATGACTACCGTCCCATAGCAC | gp4 |
| PC-5p-27588_95 | ATGACTACCGTCCCATAGCAC | gp4 |
| PC-5p-118086_5 | CTCTGGAAGCTCAGTGATGTTC | gp4 |
| PC-5p-118086_5 | CTCTGGAAGCTCAGTGATGTTC | gp4 |
| PC-5p-60839_20 | TTTGTTTGGGACTGAGGCTTCT | gp4 |
| PC-3p-103142_6 | AAGGCTTCACTCCCAAACTGGA | gp4 |
| PC-5p-115182_5 | AGTGGCGGCTCTCAGTAAGGTG | gp4 |
| PC-3p-199109_2 | TGCTAAAGCGACGGCTTGG | gp4 |
| PC-5p-74436_12 | TCAGCTGTTGCTTCTCGTATCC | gp4 |
| PC-3p-83996_9 | ATACGACAGACGAGAGCTGATC | gp4 |
| PC-3p-273727_2 | GAGATGACAGAGAAAAAGAT | gp4 |
| PC-5p-235135_2 | TGATTTCCAATAATTGAGACAGA | gp4 |
| PC-3p-182432_2 | TGGAGCTGCTCTGGACACCATCCAGT | gp4 |
| PC-5p-76231_11 | TGAAGGTTGTTGTGTGAACTGA | gp4 |
| PC-3p-26418_100 | CTGAGAACGGTCTGGGATTAAT | gp4 |
| PC-5p-1932_1060 | ATGACTACCGTCCCATAGCACT | gp4 |
| PC-3p-95588_7 | TCAGACACATTACTGAAACAGA | gp4 |
| PC-5p-192240_2 | ATGGCGGCGGTGACGACA | gp4 |
| PC-5p-46804_37 | TATGGGCACAGCAAGCTAATTG | gp4 |
| PC-5p-139502_3 | ACACAGCTAGCTGTTAGCTCT | gp4 |
| PC-5p-182537_2 | GATGAGACGGTTAGCACTGCAGC | gp4 |
| PC-5p-108905_5 | TGCAGTCTGTTCCTGTCCTGT | gp4 |
| PC-3p-64470_17 | TTATCGTGTTGGTTTGGTGTAA | gp4 |
| PC-5p-89171_8 | TAAGGTACAGTTGAATAAAGAT | gp4 |
| PC-5p-71280_13 | TTAAGGCACAGTTGAATTAAGAT | gp4 |
| PC-5p-49518_32 | TCTTCCTTATACGCTGTGCTC | gp4 |
| PC-5p-62015_19 | CATGACTAGCCTCTCAAAGCA | gp4 |
| PC-3p-88558_8 | TCTCTGTAGCTGAAATCACCA | gp4 |
| PC-5p-30932_80 | TGGTGGAGTCTGAGGAGA | gp4 |
| PC-5p-280344_2 | AAGTGGCGTCCGGAGAACTG | gp4 |
| PC-3p-179146_2 | GAGAGTGGGGCTGAAGATGGCA | gp4 |
| PC-3p-410541_1 | GTGTAGTATGACAGAGTCCATTATTG | gp4 |
| PC-5p-336263_1 | TGAGAGTGTGTGTGTGAGAGT | gp4 |
| PC-5p-64350_17 | AGCGGACATGAGCATGTGACAT | gp4 |
| PC-3p-53505_27 | CTCCAGCCGGAGGCGAACCAGA | gp4 |
| PC-3p-108349_5 | ATTCTCCCTCCGTACTCGTTGC | gp4 |
| PC-3p-116019_5 | CTCCTCCTCCTCCTCTAGCCC | gp4 |
| PC-5p-283554_2 | TGTACTGACTGGTTACAGAGA | gp4 |
| PC-5p-119589_4 | TCGTTTCTTGCAGCGCCGAAC | gp4 |
| PC-5p-58465_22 | CATGACTAGCCTCTCAAAGCACT | gp4 |
| PC-5p-181690_2 | TTTCAAACGCTTCTGGACATGT | gp4 |

| Item | Description |
| --- | --- |
| miR_name | The miR_name is composed of the 1st known miR name in a cluster, a underscore, and a matching annotation: such as |
|  | L-n means the miRNA_seq (detected) is n base less than known rep_miRSeq in the left side; |
|  | R-n means the miRNA_seq (detected) is n base less than known rep_miRSeq in the right side; |
|  | L+n means the miRNA_seq (detected) is n base more than known rep_miRSeq in the left side; |
|  | R+n means the miRNA_seq (detected) is n base more than known rep_miRSeq in the right side; |
|  | 2ss5TC13TA means 2 substitutin (ss), which are T->C at position 5 and T->A at position 13 |
|  | if there is no matching annotation, the miRNA_seq (detected) is exactly same as known rep_miRSeq. |
|  | New discovered 5p/3p sequence has been annotated as p3/p5: which is directly differentiate with the reported sequences, |
| Sequence in miRbase | New, miRNA identified in this study and not reported in miRBase,mainly for new reported 5p or 3p sequence; Diff, confirming miRNA sequences in miRBase, but different sequences are reported in our study; and Yes, confirming miRNA sequences in miRBase. |
|  | gp1:Reads were mapped to miRNAs/pre-miRNAs of specific species in miRbase and the pre-miRNAs were further mapped to genome & EST. |
|  | gp2:Reads were mapped to miRNAs/pre-miRNAs of selected species in miRbase and the mapped pre-miRNAs were not further mapped to genome, but the reads (and of course the miRNAs of the pre-miRNAs) were mapped to genome. The extended genome sequences from the genome loci may form hairpins. |
|  | gp3:Reads were mapped to miRNAs/pre-miRNAs of selected species in miRbase and the mapped pre-miRNAs were not further mapped to genome, and the reads were not mapped to genome, either. but the reads were mapped to the miRNAS(Matures) |
|  | gp4:Reads were not mapped to pre-miRNAs of selected species in miRbase. But the reads were mapped to genome & the extended genome sequences from genome may form hairpins. |

Table S7 List of miRNA member in each family in P. vachelli.

| AC | ID | MI |
| --- | --- | --- |
| MIPF0000001 | mir-17 | dre-miR-18a_1ss11CT,dre-miR-93_R+1,cgr-miR-106b-5p_R-2_1ss10GT,cgr-miR-106b-3p_L+2R-2,ccr-miR-17-5p,ccr-miR-17-3p  ,ccr-miR-17-5p,ccr-miR-17-3p  ,ccr-miR-18c_R-3,ssa-miR-18a-3p_R-3_2ss10GA11TG,ssa-miR-20a-2-3p_R-1,ssa-miR-20a-5p |
| MIPF0000002 | let-7 | hsa-let-7d-5p_1ss16CT,rno-let-7b-5p,rno-let-7b-3p,gga-let-7a-5p,gga-let-7a-5p,gga-let-7a-3p,tni-let-7i,mdo-let-7a-5p,pma-let-7b-5p_1ss15TG,aca-let-7i-5p,  aca-let-7i-3p_R-1_1ss9GT,ccr-let-7j,pmi-let-7-5p_1ss10CA,ssa-let-7g-3p_2ss8GA22CT,ssa-let-7i-2-3p_2ss9GT22CT,cfa-let-7d_R-3 |
| MIPF0000003 | mir-430 | dre-miR-430a-3p,ccr-miR-430 |
| MIPF0000005 | mir-30 | dre-miR-30e-5p_R+2,dre-miR-30e-3p,dre-miR-30e-5p_R+2,dre-miR-30e-3p,ipu-miR-30d,ssa-miR-30a-5p,ssa-miR-30a-4-3p_1ss12GA |
| MIPF0000006 | mir-15 | fru-miR-16,dre-miR-15c_R+1_1ss10GA,bta-miR-195_1ss11GT,tgu-miR-16b-5p,pma-miR-15b_R-1_1ss21CT,ccr-miR-15b,ccr-miR-457a_L+1R+1,  ssa-miR-16a-3p_R+1_2ss10TA11TC |
| MIPF0000007 | mir-181 | gga-miR-181b-5p_R+1,gga-miR-181b-2-3p_L+1,pma-miR-181a-5p,pma-miR-181a-3p,pma-miR-181a-5p,pma-miR-181a-3p,chi-miR-181b-5p |
| MIPF0000009 | mir-29 | hsa-miR-29b-3p_R-1,hsa-miR-29b-2-5p_R+1_1ss18CA,cgr-miR-29c-5p_1ss11CT,cgr-miR-29c-3p_R+1,ccr-miR-29a,ipu-miR-29b_1ss11GA,  ssa-miR-29b-1-5p_1ss18TC,ssa-miR-29b-2-5p_L-2_2ss12CT20TC,ssa-miR-29b-3p |
| MIPF0000011 | mir-19 | dre-miR-19b-3p_R-1,aca-miR-19a-5p_L-1R+1_1ss20TC,aca-miR-19a-3p_R+1,ssa-miR-19c-3p,ssa-miR-19c-4-5p_R+1_2ss17TA21AT,  ssa-miR-19c-4-5p_R+1_2ss17TA21AT |
| MIPF0000013 | mir-25 | dre-miR-92a-3p,xtr-miR-92a_R+1,bta-miR-25,sko-miR-92c_R+2_1ss10CT,pma-miR-25b-3p_R-2_1ss10AT,bbe-miR-92d-3p_1ss15TC,  ssa-miR-25-5p_2ss9TA23CT |
| MIPF0000014 | mir-9 | aca-miR-9-5p_R+3,aca-miR-9-1-3p_R+2 |
| MIPF0000018 | mir-154 | hsa-miR-494-3p_R+2,bta-miR-410,bta-miR-494_R+2,ssc-miR-369 |
| MIPF0000019 | mir-8 | fru-miR-200a_R+1,xtr-miR-200b,xtr-miR-429,bta-miR-200c_R-1_1ss22GA,aca-miR-200b-5p_R+2,aca-miR-200b-3p_R+1,  pmi-miR-200-3p_1ss9TC,ssa-miR-200a-2-5p_2ss9GT10AG,ssa-miR-200b-5p,ssa-miR-429-5p_R+1 |
| MIPF0000021 | mir-124 | dre-miR-124-5p,aca-miR-124b_R-1,oha-miR-124-5p,oha-miR-124-4-3p_R-2 |
| MIPF0000022 | mir-7 | rno-miR-7a-5p,ccr-miR-7b_R+1 |
| MIPF0000024 | mir-103 | fru-miR-103,pma-miR-103a_R-2_1ss9CT,ssa-miR-107-5p_R+1_3ss4TC12AG16TC |
| MIPF0000026 | mir-218 | ccr-miR-218a_R+1,ssa-miR-218-3p_L+2R-3 |
| MIPF0000027 | mir-23 | pma-miR-23b_R-2 |
| MIPF0000028 | mir-135 | dre-miR-135b-5p_R+1,dre-miR-135b-3p_L-1,ccr-miR-135c,ssa-miR-135b-3-3p_R+1_1ss10CT |
| MIPF0000029 | mir-133 | rno-miR-133c_L+1R+2,aca-miR-133a_L-1R+1,ccr-miR-133a-5p_R+1,ccr-miR-133a-3p,bta-miR-133c_L-1R+2,ssa-miR-133a-5p_R+1,  oha-miR-133b-3p_R-2 |
| MIPF0000031 | mir-196 | age-miR-196_R+1 |
| MIPF0000033 | mir-10 | dre-miR-10b-5p_R-1,dre-miR-10b-3p_L-1,dre-miR-10c-3p_L+1R-1,bfl-miR-125b_L-2R+2,aca-miR-125a-5p,sha-miR-125a,ccr-miR-10c_R-1,  bbe-miR-100-5p,bbe-miR-100-3p_1ss10GC,ssa-miR-125a-5p,ssa-miR-125a-2-3p_3ss20CG21GC22CT,ssa-miR-99-5p,efu-miR-125a,efu-miR-99b_R-3 |
| MIPF0000034 | mir-130 | dre-miR-130b,dre-miR-130c-5p,dre-miR-130c-3p_R-1,dre-miR-301c-5p_R+1_1ss4CT,dre-miR-301c-3p_R+1,dre-miR-301c-5p_R+1_1ss4CT,  ccr-miR-130c_R-1,ssa-miR-130a-5p_1ss9CT,ssa-miR-130a-2-3p_R-1,ssa-miR-130a-5p_1ss9CT,ssa-miR-130d-2-5p_2ss9AT13CT,ssa-miR-301a-3p |
| MIPF0000036 | mir-27 | dre-miR-27b-5p_R-1,dre-miR-27b-3p_R-1,tni-miR-27e_R-1_1ss19AT,cgr-miR-27a-3p_R+1_1ss19CT |
| MIPF0000038 | mir-1 | pma-miR-1a-3p_R-1_1ss18CT,aca-miR-1a-3p,aca-miR-1a-3p,aca-miR-1a-2-5p_R+3,aca-miR-1a-2-5p_R+3,ola-miR-206_R+1,ssa-miR-206-5p_R+1 |
| MIPF0000039 | mir-34 | hsa-miR-34c-5p,dre-miR-34a,dre-miR-34b_L-1R+1,dre-miR-34c-3p_1ss22GC |
| MIPF0000040 | mir-199 | pma-miR-199a-5p_L+3,pma-miR-199a-5p_L+3,pma-miR-199b-3p_L+2R-1,pma-miR-199b-3p_L+2R-1,ipu-miR-199a-5p,ssa-miR-199a-3p_1ss10TC |
| MIPF0000041 | mir-24 | dre-miR-24_R+1,ssa-miR-24b-5p_1ss17AT |
| MIPF0000042 | mir-204 | aca-miR-204a-5p,oha-miR-204-5p |
| MIPF0000043 | mir-26 | hsa-miR-26a-5p,hsa-miR-26a-2-3p_R+1_1ss20TG,dre-miR-26a-5p,dre-miR-26a-2-3p_4ss4AG8AT11AG19CT |
| MIPF0000044 | mir-219 | hsa-miR-219a-5p,dre-miR-219-5p_R-1,dre-miR-219-5p_R-1,gga-miR-219b_L-3R+2,hsa-miR-219b-3p_L+1R-3_1ss21CT,ipu-miR-219a,  ssa-miR-219a-3p_1ss11AC,ssa-miR-219b-5p_R-2 |
| MIPF0000046 | mir-101 | dre-miR-101a,ssa-miR-101a-5p_R-1_1ss12TA |
| MIPF0000048 | mir-128 | oha-miR-128-3p_R-1 |
| MIPF0000050 | mir-153 | ccr-miR-153b,ssa-miR-153a-2-5p_2ss5CT12AG |
| MIPF0000051 | mir-221 | dre-miR-222a-5p,dre-miR-222a-3p_R-1,dre-miR-222a-5p,dre-miR-222a-3p_R-1,bta-miR-221,cgr-miR-221-5p_R-4,cgr-miR-221-3p_R-1,  gga-miR-222b-5p_R-7,gga-miR-222b-3p_R-1,ssa-miR-221-3p_R-1_1ss5AG |
| MIPF0000053 | mir-22 | dre-miR-22a-5p_R-1,dre-miR-22a-3p |
| MIPF0000054 | mir-216 | pma-miR-216b_R-1_1ss1AT |
| MIPF0000055 | mir-194 | aca-miR-194-5p,aca-miR-194-5p,aca-miR-194-2-3p_R-2_1ss11GT,ssa-miR-194a-3p_R-2_2ss10AC20CT |
| MIPF0000056 | mir-148 | dre-miR-148_1ss10TA |
| MIPF0000057 | mir-28 | hsa-miR-28-5p,bta-miR-151-5p_R+1,bta-miR-151-3p_R+2_1ss10AG,bta-miR-151-5p_R+1,bta-miR-151-3p_R+2_1ss10AG,bta-miR-28 |
| MIPF0000058 | mir-205 | oan-miR-205-5p_R+2,ssa-miR-205b-2-3p_R-2_1ss20GA,ssa-miR-205b-2-3p_1ss20GA,ssa-miR-205b-5p_R+1 |
| MIPF0000059 | mir-184 | ccr-miR-184 |
| MIPF0000060 | mir-21 | fru-miR-21,mdo-miR-21-5p_L+1_1ss17AG,ssa-miR-21b-3p_L-1R+1 |
| MIPF0000061 | mir-365 | dre-miR-365_1ss11AG,ssa-miR-365-5p_R+3,oha-miR-365a-3p_1ss11AG,oha-miR-365a-2-5p_1ss10CT |
| MIPF0000062 | mir-214 | hsa-miR-3120-5p_L-1R+2,hsa-miR-3120-3p_R+2,hsa-miR-3120-5p_L-1R+2,hsa-miR-3120-3p_R+2,aca-miR-214-5p,aca-miR-214-3p |
| MIPF0000063 | mir-192 | ola-miR-192-5p_L+1R-1,ola-miR-192-3p_R-1,ccr-miR-192,ssa-miR-192a-3p_1ss11AC |
| MIPF0000064 | mir-31 | dre-miR-31_L+1R+1,aca-miR-31-5p_R+2_1ss1AT |
| MIPF0000065 | mir-132 | hsa-miR-212-5p,xtr-miR-132,xtr-miR-212,ipu-miR-132a,ipu-miR-212,ssa-miR-212a-3p,ssa-miR-212b-5p |
| MIPF0000066 | mir-183 | bta-miR-183 |
| MIPF0000067 | mir-223 | bta-miR-223,ipu-miR-223_1ss20GT |
| MIPF0000069 | mir-32 | aca-miR-32-5p_R-1 |
| MIPF0000070 | mir-33 | gga-miR-33-5p,gga-miR-33-3p_L+1_1ss20TC,ssa-miR-33b-5p_R-1,ssa-miR-33b-3p_R-2 |
| MIPF0000072 | mir-96 | dre-miR-96-5p,dre-miR-96-3p_R+1 |
| MIPF0000073 | mir-129 | bta-miR-129_R-1,bta-miR-129-3p,bta-miR-129_R-1,bta-miR-129-3p |
| MIPF0000075 | mir-138 | aca-miR-138-5p_R+1,ssa-miR-138-3p_R+1 |
| MIPF0000076 | mir-190 | tni-miR-190_R+1,ssa-miR-190a-3p |
| MIPF0000077 | mir-217 | bta-miR-217_R-1 |
| MIPF0000078 | mir-187 | gga-miR-187-5p_L+1_2ss6AG23AT,ccr-miR-187 |
| MIPF0000079 | mir-145 | mdo-miR-145-5p_R-1,mdo-miR-145-3p_L+2 |
| MIPF0000080 | mir-127 | bta-miR-127 |
| MIPF0000082 | mir-193 | dre-miR-193b-3p,gga-miR-193b-5p_1ss22AT,gga-miR-193b-3p_1ss10AG,gga-miR-193a-5p_R-1,gga-miR-193a-3p_R-2,  gga-miR-193a-5p_R-1,gga-miR-193a-3p_R-2 |
| MIPF0000084 | mir-142 | dre-miR-142a-5p_L+2R-1,dre-miR-142a-5p_L+2R-1,dre-miR-142a-3p_R-1,dre-miR-142a-3p_R-1,mmu-miR-142b_R+2_1ss16AG |
| MIPF0000085 | mir-140 | oan-miR-140-5p_R+2,oan-miR-140-3p,sha-miR-140_L+1,ssa-miR-140-5p |
| MIPF0000086 | mir-210 | ola-miR-210-5p_R+2_1ss20TC,ola-miR-210-3p_L-1R+3,cgr-miR-210-3p_R-2 |
| MIPF0000087 | mir-46 | odi-miR-281_1ss21GT |
| MIPF0000088 | mir-224 | bta-miR-224_R+3 |
| MIPF0000093 | mir-144 | gga-miR-144-5p_R+1,gga-miR-144-3p_R-2,cfa-miR-144_L+1R-3,pma-miR-144-5p_L-1R-2_1ss10AC,pma-miR-144-3p_R-2_1ss18GA,  ssa-miR-144-5p_R-1 |
| MIPF0000094 | mir-143 | ccr-miR-143_R+1_1ss20TA,ssa-miR-143-5p |
| MIPF0000095 | mir-122 | hsa-miR-3591-5p_L-1,hsa-miR-3591-3p_1ss22CT,ipu-miR-122_R+3,ssa-miR-122-3p_R+1_2ss12TC19TA,ssa-miR-122-5p_R-1,ssa-miR-122-2-3p_R-1 |
| MIPF0000097 | mir-338 | dre-miR-338_R-1,ppy-miR-338-3p_R-1,ssa-miR-338a-5p_1ss20AC |
| MIPF0000098 | mir-95 | hsa-miR-545-3p_1ss22CT,mmu-miR-421-3p_1ss23CT,chi-miR-421-3p_1ss23CT |
| MIPF0000103 | mir-146 | dre-miR-146b_R+1,hsa-miR-146b-5p_R-3,ccr-miR-146a,ssa-miR-146b-3p_R-1_2ss14AG20CT |
| MIPF0000106 | mir-137 | tni-miR-137_R+1,xbo-miR-137_L+1R-1_1ss18AG,ola-miR-137_L+3R-2,ssa-miR-137-5p_R+1 |
| MIPF0000108 | mir-203 | ccr-miR-203a,ccr-miR-203a,ccr-miR-203b-5p_R-1,ccr-miR-203b-5p_R-1,ipu-miR-203c |
| MIPF0000109 | mir-186 | mdo-miR-186-5p |
| MIPF0000110 | mir-329 | mmu-miR-329-5p |
| MIPF0000111 | mir-489 | aca-miR-489-5p,aca-miR-489-3p_L-1R+1 |
| MIPF0000113 | mir-188 | bta-miR-532_R-2,cgr-miR-532-5p_R-2 |
| MIPF0000114 | mir-375 | tni-miR-375_R-1 |
| MIPF0000115 | mir-126 | ola-miR-126-5p,ola-miR-126-3p_R+2,ola-miR-126-5p,ola-miR-126-3p_R+2 |
| MIPF0000116 | mir-182 | aca-miR-182-5p_1ss10GA,aca-miR-182-3p,aca-miR-182-5p_1ss10GA,aca-miR-182-3p |
| MIPF0000117 | mir-139 | tgu-miR-139-5p,ola-miR-139_R+2 |
| MIPF0000121 | mir-202 | dre-miR-202-5p_R-1,dre-miR-202-3p_L+2R-2,dre-miR-202-5p_R-1,dre-miR-202-3p_L+2R-2,oha-miR-202-5p_R-1 |
| MIPF0000123 | mir-197 | bta-miR-197 |
| MIPF0000126 | mir-379 | bta-miR-379 |
| MIPF0000128 | mir-450 | mmu-miR-450b-3p_L-1R-1_1ss3TG |
| MIPF0000129 | mir-455 | dre-miR-455-5p,tni-miR-455,dre-miR-455-3p_L-2R+1,ssa-miR-455-3p |
| MIPF0000134 | mir-460 | ccr-miR-730_R+1,ssa-miR-460-5p_1ss15AG,ssa-miR-460-3p,ssa-miR-730a-3p |
| MIPF0000137 | mir-383 | aca-miR-383-5p_R+1,aca-miR-383-5p_R+1 |
| MIPF0000138 | mir-363 | hsa-miR-363-3p_R-1,aca-miR-363-5p,aca-miR-363-3p,sha-miR-92a_R+4 |
| MIPF0000139 | mir-500 | cgr-miR-500_R-1 |
| MIPF0000141 | mir-315 | sko-miR-315_R+2 |
| MIPF0000142 | mir-431 | bta-miR-431_R-3 |
| MIPF0000148 | mir-451 | dre-miR-451_R-1,dre-mir-451-p3,dre-miR-451_R-3_1ss19AT,pma-miR-451_R-4_1ss16TC,aca-miR-451-3p_L+2R+1,aca-miR-451-3p_L+2R+1_1ss18TC |
| MIPF0000157 | mir-155 | aca-miR-155-5p_R+1 |
| MIPF0000159 | mir-296 | hsa-miR-296-5p |
| MIPF0000163 | mir-320 | bta-miR-320a |
| MIPF0000164 | mir-322 | chi-miR-424-5p |
| MIPF0000165 | mir-324 | bta-miR-324_R-1,cgr-miR-324-5p |
| MIPF0000168 | mir-378 | bta-miR-378 |
| MIPF0000172 | mir-361 | efu-miR-361,chi-miR-361-5p,chi-miR-361-3p_R+2 |
| MIPF0000173 | mir-499 | aca-miR-499-5p_R-1,aca-miR-499-3p_R-1,ola-miR-499,cgr-miR-499-5p_R-1,ssa-miR-499b-5p_R-1 |
| MIPF0000174 | mir-454 | aca-miR-454-3p,ssa-miR-454-5p |
| MIPF0000179 | mir-458 | dre-miR-458-5p_R-2,dre-miR-458-3p |
| MIPF0000183 | mir-503 | bta-miR-503-5p_R+3,bta-miR-503-5p_R+3 |
| MIPF0000190 | mir-342 | cgr-miR-342-3p_R-1 |
| MIPF0000191 | mir-340 | hsa-miR-340-5p,cfa-miR-340 |
| MIPF0000193 | mir-339 | hsa-miR-339-5p_R-4,hsa-miR-339-3p_L-2_1ss3AT,hsa-miR-339-5p_R-4,ggo-miR-339_L-2_1ss3AT |
| MIPF0000194 | mir-191 | oha-miR-191-5p_R-1,oha-miR-191-5p_R-1 |
| MIPF0000196 | mir-335 | bta-miR-335 |
| MIPF0000197 | mir-150 | dre-miR-150_R+1 |
| MIPF0000199 | mir-331 | hsa-miR-331-5p_R-1 |
| MIPF0000202 | mir-185 | bta-miR-185,cgr-miR-185-5p |
| MIPF0000203 | mir-328 | bta-miR-328 |
| MIPF0000208 | mir-466 | gga-miR-466_R-1_3ss20AC21GA22AC |
| MIPF0000213 | mir-541 | mmu-miR-541-5p_R-5 |
| MIPF0000219 | mir-484 | bta-miR-484 |
| MIPF0000220 | mir-486 | mmu-miR-486a-5p,mmu-miR-486b-3p_R+1,mmu-miR-486a-5p,mmu-miR-486b-3p_R+1 |
| MIPF0000229 | mir-490 | aca-miR-490-3p |
| MIPF0000242 | mir-425 | bta-miR-425-5p_L+1R-1,tgu-miR-425-5p_R+3,tgu-miR-425-3p_R+1_1ss20TG |
| MIPF0000278 | mir-71 | bbe-miR-71-5p_R-1 |
| MIPF0000285 | mir-252 | sko-miR-252a_R-1,lva-miR-252a-5p_R-4_1ss18AT |
| MIPF0000288 | mir-374 | hsa-miR-374a-5p,bta-miR-374b_R-1 |
| MIPF0000316 | mir-467 | mmu-miR-466g_L-3_1ss6GC,rno-miR-466b-4-3p_L+2_1ss18TC,mmu-mir-466i-p5,mmu-mir-466i-p3_1ss21TC,  mmu-miR-466i-5p_L+1R+3_1ss13GA,mmu-mir-466i-p3_1ss10GA,mmu-miR-466i-5p_L-2,mmu-miR-466i-5p_L-2,mmu-mir-669f-p5,  mmu-mir-467g-p5_1ss13TC,mmu-mir-467g-p3_1ss17TC,mmu-mir-467g-p3_1ss17TC,mmu-miR-467g_L+1,bta-miR-669_L-3_1ss5GT,  mmu-miR-466m-5p_2ss14AT22AG |
| MIPF0000329 | mir-423 | bta-miR-423-5p,bta-miR-423-3p_L-1,bta-miR-423-5p,bta-miR-423-3p_L-1 |
| MIPF0000333 | mir-652 | cgr-miR-652-3p_R+1,bta-miR-652_R+1 |
| MIPF0000341 | mir-456 | gga-miR-456-5p_L+1R-4_1ss13TC,gga-miR-456-3p_1ss22AT |
| MIPF0000360 | mir-551 | ccr-miR-551_R-1 |
| MIPF0000392 | mir-877 | bta-miR-877_1ss20GA |
| MIPF0000397 | mir-708 | bta-miR-708_R+1 |
| MIPF0000418 | mir-590 | cfa-miR-590_L+1,eca-miR-590-3p_L+1 |
| MIPF0000419 | mir-574 | ggo-miR-574 |
| MIPF0000431 | mir-744 | bta-miR-744_R-1 |
| MIPF0000483 | mir-1271 | cfa-miR-1271 |
| MIPF0000509 | mir-92 | cin-miR-92c-3p_1ss18TC,csa-miR-92c_R-2_1ss9CT |
| MIPF0000531 | mir-1306 | cgr-miR-1306-3p_L+1,dre-miR-1306_R-1 |
| MIPF0000533 | mir-584 | ggo-miR-584 |
| MIPF0000558 | mir-1307 | hsa-miR-1307-5p,chi-miR-1307-5p_R+4 |
| MIPF0000559 | mir-1285 | hsa-miR-1285-3p_R-1 |
| MIPF0000579 | mir-1273 | ssc-mir-1285-p5_1ss23CA,ssc-mir-1285-p3 |
| MIPF0000649 | mir-1296 | bta-miR-1296_R+1 |
| MIPF0000702 | mir-1839 | bta-miR-1839_L-1R-1 |
| MIPF0000710 | mir-981 | pmi-miR-981-3p_R+1_1ss21AC |
| MIPF0000728 | mir-278_2 | sko-miR-278_1ss17TC |
| MIPF0000792 | mir-1788 | dre-miR-1788-5p,dre-miR-1788-3p_R+1,dre-miR-1788-5p,dre-miR-1788-3p_R+1 |
| MIPF0000805 | mir-1388 | oan-miR-1388-5p,oan-miR-1388-3p,oan-miR-1388-5p,oan-miR-1388-3p |
| MIPF0000812 | mir-2188 | dre-miR-2188-5p,dre-miR-2188-3p_L+1 |
| MIPF0000849 | mir-1677 | tgu-miR-1677-3p |
| MIPF0000893 | mir-2976 | tgu-miR-2976_R-3_1ss9GC |
| MIPF0000918 | mir-1662 | gga-miR-1662 |
| MIPF0001027 | mir-2957 | tgu-miR-2957 |
| MIPF0001031 | mir-1559 | gga-miR-1559-5p |
| MIPF0001032 | mir-2954 | tgu-miR-2954-5p_L+1,tgu-miR-2954-3p |
| MIPF0001042 | mir-2887 | bta-mir-2887-2-p5,bta-mir-2887-2-p3,bta-miR-2887_L+2R+3 |
| MIPF0001049 | mir-1451 | gga-miR-1451-5p_R+1 |
| MIPF0001322 | mir-459 | ccr-miR-459-5p_L+2R-1,ccr-miR-459-3p_R+1_1ss5GA |
| MIPF0001328 | mir-2970 | tgu-miR-2970-5p_R+1 |
| MIPF0001354 | mir-731 | ola-miR-731_R+5 |
| MIPF0001355 | mir-725 | ssa-miR-725-3p |
| MIPF0001371 | mir-727 | aca-miR-727-5p_R+2,aca-miR-727-3p_L-1R+3 |
| MIPF0001431 | mir-6526 | bta-miR-6526 |
| MIPF0001445 | mir-6134 | mml-miR-6134_R+3_1ss18GT |
| MIPF0001510 | mir-6089 | hsa-miR-6089_L-5R-1_1ss16GT |
| MIPF0001536 | mir-3084 | rno-miR-3084d_L-3_1ss19GT,rno-miR-3084d_L-3_1ss19GT |
| MIPF0001543 | mir-734 | ccr-miR-734_R+1_1ss1TC |
| MIPF0001612 | mir-722 | ccr-miR-722_L-2R+3,ssa-miR-722-3p_R-3_1ss19AT,ssa-miR-722-3p_R-3_1ss19AT,ssa-miR-722-5p_L-1R+1 |
| MIPF0001616 | mir-724 | ssa-miR-724-5p,ssa-miR-724-3p |
| MIPF0001631 | mir-2131 | gga-miR-2131-5p |
| MIPF0001689 | mir-737 | dre-miR-737-5p_R+1_1ss2TG,dre-miR-737-3p |
| MIPF0001702 | mir-4864 | bbe-miR-4864-3p_L-3R-1_1ss16CA |
| MIPF0001705 | mir-7147 | dre-miR-7147 |
| MIPF0001710 | mir-3618 | hsa-miR-3618_1ss21GA,ipu-miR-3618 |
| MIPF0001755 | mir-462 | dre-miR-462_R+1 |
| MIPF0001771 | mir-6529 | bta-miR-6529a,mml-miR-6529-5p |
| MIPF0001831 | mir-723 | ssa-miR-723-3p |
| MIPF0001916 | mir-7552 | ssa-miR-7552a-5p,ssa-miR-7552a-5p |
| MIPF0001937 | mir-1277 | hsa-miR-1277-5p_L-3R+2 |
| MIPF0001952 | mir-8873 | cfa-miR-8873a_L-3R-2_1ss11AG |
| MIPF0002063 | mir-3533 | bta-mir-3533-p5_1ss2TC,bta-mir-3533-p3_1ss20TC |
| MIPF0002098 | mir-7132 | ccr-miR-7132_1ss11AT |
| MIPF0002102 | mir-1814 | bta-miR-1814c_R-1_1ss15TA |

Table S8 List of differentially expressed miRNAs of *P. vachelli* in response to hypoxia.

| Index | miR_name | miR_seq | up/down | log2(fold_change) | pvalue(t_test) | P0(mean) | P4(mean) | Expression level |
| --- | --- | --- | --- | --- | --- | --- | --- | --- |
| 1 | ccr-miR-187 | TCGTGTCTTGTGTTGCAGCCAGT | up | 1.32 | 2.20E-03 | 5 | 12 | middle |
| 2 | dre-miR-301c-3p_R+1 | CAGTGCAATAGTATTGTCATAGC | down | -0.95 | 1.14E-02 | 273 | 141 | middle |
| 3 | ssa-miR-301a-3p | CAGTGCAATAGTATTGTCATAGC | down | -0.95 | 1.14E-02 | 273 | 141 | middle |
| 4 | dre-miR-193b-3p | AACTGGCCCGCAAAGTCCCGCT | up | 0.73 | 1.19E-02 | 59 | 98 | middle |
| 5 | PC-5p-48126_34 | CATGACTAGCCTCTCAAAGCAC | down | -1.09 | 1.48E-02 | 4 | 2 | low |
| 6 | ssa-miR-20a-5p | TAAAGTGCTTATAGTGCAGGTAG | down | -0.73 | 2.63E-02 | 14,527 | 8,731 | middle |
| 7 | PC-3p-36625_60 | TGCTTTGAGAGGCTAGTCATGA | down | -0.98 | 2.81E-02 | 17 | 9 | middle |
| 8 | PC-5p-83983_9 | GCTTATTGTGAGTCCCGGGTA | down | -1.93 | 2.99E-02 | 7 | 2 | low |
| 9 | ola-miR-210-5p_R+2_1ss20TC | AGCCACTGACTAACGCACACTG | up | 2.28 | 3.10E-02 | 465 | 2,253 | middle |
| 10 | dre-miR-338_R-1 | TCCAGCATCAGTGATTTTGTT | down | -0.52 | 3.14E-02 | 318 | 221 | middle |
| 11 | ppy-miR-338-3p_R-1 | TCCAGCATCAGTGATTTTGTT | down | -0.52 | 3.14E-02 | 318 | 221 | middle |
| 12 | pma-miR-181a-3p | ACCATCGACCGTTGACTGTACC | up | 0.74 | 3.62E-02 | 550 | 919 | middle |
| 13 | ccr-miR-143_R+1_1ss20TA | TGAGATGAAGCACTGTAGCAC | up | 0.40 | 3.63E-02 | 18,398 | 24,325 | high |
| 14 | ssa-miR-16a-3p_R+1_2ss10TA11TC | CCAGTATTGACCGTGCTGCTGAA | down | -1.09 | 3.68E-02 | 16 | 8 | middle |
| 15 | hsa-miR-3618_1ss21GA | TGTCTACATTAATGAAAAGAAC | down | -inf | 4.10E-02 | 1 | 0 | low |
| 16 | ccr-miR-17-5p | CAAAGTGCTTACAGTGCAGGTAG | down | -0.62 | 4.67E-02 | 17,035 | 11,047 | high |
| 17 | dre-miR-27b-3p_R-1 | TTCACAGTGGCTAAGTTCTGC | up | 0.57 | 4.84E-02 | 20,505 | 30,381 | high |
| 18 | ssa-miR-200b-5p | CATCTTACCTGACAGTGCTGGA | up | 0.47 | 4.88E-02 | 40 | 56 | middle |

Table S9 List of miRNA-mRNA pairs with positive and negative correlation.

| miR_name | miR_seq | regulation | Accession | annotation | regulation |
| --- | --- | --- | --- | --- | --- |
| ssa-miR-20a-5p | TAAAGTGCTTATAGTGCAGGTAG | down | comp10260_c0 | - | up |
| PC-5p-83983_9 | GCTTATTGTGAGTCCCGGGTA | down | comp10326_c0 | - | down |
| PC-5p-83983_9 | GCTTATTGTGAGTCCCGGGTA | down | comp10699_c0 | - | up |
| ccr-miR-187 | TCGTGTCTTGTGTTGCAGCCAGT | up | comp10699_c0 | - | up |
| ccr-miR-17-5p | CAAAGTGCTTACAGTGCAGGTAG | down | comp10699_c2 | - | up |
| ssa-miR-20a-5p | TAAAGTGCTTATAGTGCAGGTAG | down | comp10699_c2 | - | up |
| hsa-miR-3618_1ss21GA | TGTCTACATTAATGAAAAGAAC | down | comp11189_c0 | - | up |
| PC-3p-36625_60 | TGCTTTGAGAGGCTAGTCATGA | down | comp11248_c0 | methylsterol monooxygenase | up |
| ccr-miR-17-5p | CAAAGTGCTTACAGTGCAGGTAG | down | comp11248_c0 | methylsterol monooxygenase | up |
| ssa-miR-20a-5p | TAAAGTGCTTATAGTGCAGGTAG | down | comp11248_c0 | methylsterol monooxygenase | up |
| ccr-miR-143_R+1_1ss20TA | TGAGATGAAGCACTGTAGCAC | up | comp11294_c0 | layilin | down |
| PC-3p-36625_60 | TGCTTTGAGAGGCTAGTCATGA | down | comp11635_c0 | - | down |
| dre-miR-27b-3p_R-1 | TTCACAGTGGCTAAGTTCTGC | up | comp11803_c0 | fatty acid desaturase 2 (delta-6 desaturase) | up |
| ssa-miR-16a-3p_R+1_2ss10TA11TC | CCAGTATTGACCGTGCTGCTGAA | down | comp11803_c0 | fatty acid desaturase 2 (delta-6 desaturase) | up |
| PC-3p-36625_60 | TGCTTTGAGAGGCTAGTCATGA | down | comp11888_c0 | protein phosphatase 3, regulatory subunit | up |
| ccr-miR-17-5p | CAAAGTGCTTACAGTGCAGGTAG | down | comp11983_c0 | glucose-6-phosphatase | down |
| ssa-miR-20a-5p | TAAAGTGCTTATAGTGCAGGTAG | down | comp11983_c0 | glucose-6-phosphatase | down |
| PC-5p-83983_9 | GCTTATTGTGAGTCCCGGGTA | down | comp12230_c0 | carbonic anhydrase | up |
| ccr-miR-143_R+1_1ss20TA | TGAGATGAAGCACTGTAGCAC | up | comp12230_c0 | carbonic anhydrase | up |
| PC-5p-83983_9 | GCTTATTGTGAGTCCCGGGTA | down | comp12233_c0 | myosin IX | up |
| ssa-miR-16a-3p_R+1_2ss10TA11TC | CCAGTATTGACCGTGCTGCTGAA | down | comp12317_c0 | diphosphoinositol-polyphosphate diphosphatase | up |
| PC-3p-36625_60 | TGCTTTGAGAGGCTAGTCATGA | down | comp12482_c0 | apoptosis regulator BCL-2 | up |
| dre-miR-27b-3p_R-1 | TTCACAGTGGCTAAGTTCTGC | up | comp12539_c1 | - | down |
| dre-miR-338_R-1 | TCCAGCATCAGTGATTTTGTT | down | comp12539_c1 | - | down |
| hsa-miR-3618_1ss21GA | TGTCTACATTAATGAAAAGAAC | down | comp12539_c1 | - | down |
| ppy-miR-338-3p_R-1 | TCCAGCATCAGTGATTTTGTT | down | comp12539_c1 | - | down |
| ccr-miR-143_R+1_1ss20TA | TGAGATGAAGCACTGTAGCAC | up | comp12627_c0 | nuclear factor of kappa light polypeptide gene enhancer in B-cells inhibitor, alpha | up |
| PC-5p-83983_9 | GCTTATTGTGAGTCCCGGGTA | down | comp12673_c0 | - | up |
| dre-miR-27b-3p_R-1 | TTCACAGTGGCTAAGTTCTGC | up | comp12753_c0 | - | up |
| dre-miR-301c-3p_R+1 | CAGTGCAATAGTATTGTCATAGC | down | comp12753_c0 | - | up |
| ssa-miR-301a-3p | CAGTGCAATAGTATTGTCATAGC | down | comp12753_c0 | - | up |
| PC-5p-48126_34 | CATGACTAGCCTCTCAAAGCAC | down | comp12927_c0 | Ras-related protein Rab-20 | up |
| dre-miR-27b-3p_R-1 | TTCACAGTGGCTAAGTTCTGC | up | comp12927_c0 | Ras-related protein Rab-20 | up |
| ola-miR-210-5p_R+2_1ss20TC | AGCCACTGACTAACGCACACTG | up | comp12927_c0 | Ras-related protein Rab-20 | up |
| dre-miR-301c-3p_R+1 | CAGTGCAATAGTATTGTCATAGC | down | comp13162_c0 | - | down |
| hsa-miR-3618_1ss21GA | TGTCTACATTAATGAAAAGAAC | down | comp13162_c0 | - | down |
| ssa-miR-301a-3p | CAGTGCAATAGTATTGTCATAGC | down | comp13162_c0 | - | down |
| ccr-miR-17-5p | CAAAGTGCTTACAGTGCAGGTAG | down | comp13182_c0 | tubulin gamma | down |
| ssa-miR-20a-5p | TAAAGTGCTTATAGTGCAGGTAG | down | comp13182_c0 | tubulin gamma | down |
| ccr-miR-143_R+1_1ss20TA | TGAGATGAAGCACTGTAGCAC | up | comp13235_c0 | - | up |
| dre-miR-301c-3p_R+1 | CAGTGCAATAGTATTGTCATAGC | down | comp13235_c0 | - | up |
| ssa-miR-301a-3p | CAGTGCAATAGTATTGTCATAGC | down | comp13235_c0 | - | up |
| ssa-miR-16a-3p_R+1_2ss10TA11TC | CCAGTATTGACCGTGCTGCTGAA | down | comp13255_c1 | bromodomain-containing protein 4 | up |
| ssa-miR-20a-5p | TAAAGTGCTTATAGTGCAGGTAG | down | comp13393_c0 | histone H1/5 | down |
| ccr-miR-17-5p | CAAAGTGCTTACAGTGCAGGTAG | down | comp13563_c0 | mitochondrial brown fat uncoupling protein 1 | up |
| ssa-miR-20a-5p | TAAAGTGCTTATAGTGCAGGTAG | down | comp13563_c0 | mitochondrial brown fat uncoupling protein 1 | up |
| PC-3p-36625_60 | TGCTTTGAGAGGCTAGTCATGA | down | comp13907_c0 | angiopoietin-like 4 | up |
| ccr-miR-17-5p | CAAAGTGCTTACAGTGCAGGTAG | down | comp13915_c0 | phosphoglycerate mutase | up |
| ssa-miR-20a-5p | TAAAGTGCTTATAGTGCAGGTAG | down | comp13915_c0 | phosphoglycerate mutase | up |
| ccr-miR-143_R+1_1ss20TA | TGAGATGAAGCACTGTAGCAC | up | comp13969_c0 | - | down |
| dre-miR-301c-3p_R+1 | CAGTGCAATAGTATTGTCATAGC | down | comp13969_c0 | - | down |
| dre-miR-338_R-1 | TCCAGCATCAGTGATTTTGTT | down | comp13969_c0 | - | down |
| ppy-miR-338-3p_R-1 | TCCAGCATCAGTGATTTTGTT | down | comp13969_c0 | - | down |
| ssa-miR-301a-3p | CAGTGCAATAGTATTGTCATAGC | down | comp13969_c0 | - | down |
| PC-3p-36625_60 | TGCTTTGAGAGGCTAGTCATGA | down | comp14143_c0 | - | down |
| ssa-miR-20a-5p | TAAAGTGCTTATAGTGCAGGTAG | down | comp14207_c0 | - | down |
| ssa-miR-16a-3p_R+1_2ss10TA11TC | CCAGTATTGACCGTGCTGCTGAA | down | comp14343_c0 | cysteamine dioxygenase | up |
| ssa-miR-20a-5p | TAAAGTGCTTATAGTGCAGGTAG | down | comp14380_c0 | NAD+ kinase | down |
| ccr-miR-143_R+1_1ss20TA | TGAGATGAAGCACTGTAGCAC | up | comp14388_c0 | - | down |
| PC-3p-36625_60 | TGCTTTGAGAGGCTAGTCATGA | down | comp14450_c0 | - | down |
| dre-miR-301c-3p_R+1 | CAGTGCAATAGTATTGTCATAGC | down | comp14576_c0 | protein-tyrosine phosphatase | up |
| ssa-miR-301a-3p | CAGTGCAATAGTATTGTCATAGC | down | comp14576_c0 | protein-tyrosine phosphatase | up |
| PC-5p-48126_34 | CATGACTAGCCTCTCAAAGCAC | down | comp14617_c0 | - | up |
| dre-miR-27b-3p_R-1 | TTCACAGTGGCTAAGTTCTGC | up | comp14617_c0 | - | up |
| ssa-miR-16a-3p_R+1_2ss10TA11TC | CCAGTATTGACCGTGCTGCTGAA | down | comp14617_c0 | - | up |
| dre-miR-27b-3p_R-1 | TTCACAGTGGCTAAGTTCTGC | up | comp14714_c0 | ephrin-B | up |
| dre-miR-301c-3p_R+1 | CAGTGCAATAGTATTGTCATAGC | down | comp14797_c0 | MFS transporter, MCP family, solute carrier family 16 (monocarboxylic acid transporters), member 3 | up |
| ssa-miR-301a-3p | CAGTGCAATAGTATTGTCATAGC | down | comp14797_c0 | MFS transporter, MCP family, solute carrier family 16 (monocarboxylic acid transporters), member 3 | up |
| dre-miR-338_R-1 | TCCAGCATCAGTGATTTTGTT | down | comp14901_c0 | LIM domain kinase 2 | up |
| ppy-miR-338-3p_R-1 | TCCAGCATCAGTGATTTTGTT | down | comp14901_c0 | LIM domain kinase 2 | up |
| dre-miR-338_R-1 | TCCAGCATCAGTGATTTTGTT | down | comp14912_c1 | - | down |
| ppy-miR-338-3p_R-1 | TCCAGCATCAGTGATTTTGTT | down | comp14912_c1 | - | down |
| dre-miR-27b-3p_R-1 | TTCACAGTGGCTAAGTTCTGC | up | comp14947_c0 | - | up |
| dre-miR-27b-3p_R-1 | TTCACAGTGGCTAAGTTCTGC | up | comp14960_c0 | peptidyl-prolyl isomerase H (cyclophilin H) | down |
| ssa-miR-16a-3p_R+1_2ss10TA11TC | CCAGTATTGACCGTGCTGCTGAA | down | comp14960_c0 | peptidyl-prolyl isomerase H (cyclophilin H) | down |
| dre-miR-338_R-1 | TCCAGCATCAGTGATTTTGTT | down | comp14976_c0 | - | down |
| ola-miR-210-5p_R+2_1ss20TC | AGCCACTGACTAACGCACACTG | up | comp14976_c0 | - | down |
| ppy-miR-338-3p_R-1 | TCCAGCATCAGTGATTTTGTT | down | comp14976_c0 | - | down |
| PC-5p-83983_9 | GCTTATTGTGAGTCCCGGGTA | down | comp15086_c1 | phospholipase C, beta | down |
| dre-miR-301c-3p_R+1 | CAGTGCAATAGTATTGTCATAGC | down | comp15086_c1 | phospholipase C, beta | down |
| ssa-miR-301a-3p | CAGTGCAATAGTATTGTCATAGC | down | comp15086_c1 | phospholipase C, beta | down |
| ccr-miR-17-5p | CAAAGTGCTTACAGTGCAGGTAG | down | comp15265_c1 | - | down |
| ssa-miR-20a-5p | TAAAGTGCTTATAGTGCAGGTAG | down | comp15265_c1 | - | down |
| ccr-miR-17-5p | CAAAGTGCTTACAGTGCAGGTAG | down | comp15311_c0 | TNF receptor-associated factor 4 | up |
| ssa-miR-20a-5p | TAAAGTGCTTATAGTGCAGGTAG | down | comp15311_c0 | TNF receptor-associated factor 4 | up |
| dre-miR-301c-3p_R+1 | CAGTGCAATAGTATTGTCATAGC | down | comp15330_c2 | - | up |
| ssa-miR-301a-3p | CAGTGCAATAGTATTGTCATAGC | down | comp15330_c2 | - | up |
| dre-miR-27b-3p_R-1 | TTCACAGTGGCTAAGTTCTGC | up | comp15594_c0 | 3',5'-cyclic-nucleotide phosphodiesterase | down |
| ola-miR-210-5p_R+2_1ss20TC | AGCCACTGACTAACGCACACTG | up | comp15700_c0 | - | down |
| ssa-miR-16a-3p_R+1_2ss10TA11TC | CCAGTATTGACCGTGCTGCTGAA | down | comp15700_c0 | - | down |
| ssa-miR-200b-5p | CATCTTACCTGACAGTGCTGGA | up | comp15725_c0 | platelet derived growth factor A/B | up |
| dre-miR-193b-3p | AACTGGCCCGCAAAGTCCCGCT | up | comp15877_c0 | glycogen(starch) synthase | up |
| ssa-miR-16a-3p_R+1_2ss10TA11TC | CCAGTATTGACCGTGCTGCTGAA | down | comp15877_c0 | glycogen(starch) synthase | up |
| PC-3p-36625_60 | TGCTTTGAGAGGCTAGTCATGA | down | comp15963_c0 | mitogen-activated protein kinase kinase kinase | down |
| dre-miR-301c-3p_R+1 | CAGTGCAATAGTATTGTCATAGC | down | comp15963_c0 | mitogen-activated protein kinase kinase kinase | down |
| ssa-miR-301a-3p | CAGTGCAATAGTATTGTCATAGC | down | comp15963_c0 | mitogen-activated protein kinase kinase kinase | down |
| ccr-miR-143_R+1_1ss20TA | TGAGATGAAGCACTGTAGCAC | up | comp16016_c1 | - | down |
| dre-miR-338_R-1 | TCCAGCATCAGTGATTTTGTT | down | comp16016_c1 | - | down |
| ppy-miR-338-3p_R-1 | TCCAGCATCAGTGATTTTGTT | down | comp16016_c1 | - | down |
| dre-miR-338_R-1 | TCCAGCATCAGTGATTTTGTT | down | comp16131_c0 | von Hippel-Lindau disease tumor supressor | up |
| ppy-miR-338-3p_R-1 | TCCAGCATCAGTGATTTTGTT | down | comp16131_c0 | von Hippel-Lindau disease tumor supressor | up |
| ccr-miR-17-5p | CAAAGTGCTTACAGTGCAGGTAG | down | comp16210_c0 | - | up |
| dre-miR-301c-3p_R+1 | CAGTGCAATAGTATTGTCATAGC | down | comp16210_c0 | - | up |
| ssa-miR-20a-5p | TAAAGTGCTTATAGTGCAGGTAG | down | comp16210_c0 | - | up |
| ssa-miR-301a-3p | CAGTGCAATAGTATTGTCATAGC | down | comp16210_c0 | - | up |
| PC-5p-83983_9 | GCTTATTGTGAGTCCCGGGTA | down | comp16219_c0 | histone demethylase JMJD3 | up |
| dre-miR-338_R-1 | TCCAGCATCAGTGATTTTGTT | down | comp16342_c1 | shingomyelin synthase | up |
| ppy-miR-338-3p_R-1 | TCCAGCATCAGTGATTTTGTT | down | comp16342_c1 | shingomyelin synthase | up |
| ccr-miR-17-5p | CAAAGTGCTTACAGTGCAGGTAG | down | comp16358_c0 | actinin alpha | down |
| ssa-miR-20a-5p | TAAAGTGCTTATAGTGCAGGTAG | down | comp16358_c0 | actinin alpha | down |
| ccr-miR-17-5p | CAAAGTGCTTACAGTGCAGGTAG | down | comp16360_c0 | dual specificity phosphatase | up |
| PC-3p-36625_60 | TGCTTTGAGAGGCTAGTCATGA | down | comp16469_c0 | - | up |
| ccr-miR-17-5p | CAAAGTGCTTACAGTGCAGGTAG | down | comp16593_c0 | cAMP response element modulator | up |
| dre-miR-27b-3p_R-1 | TTCACAGTGGCTAAGTTCTGC | up | comp16593_c0 | cAMP response element modulator | up |
| ssa-miR-20a-5p | TAAAGTGCTTATAGTGCAGGTAG | down | comp16593_c0 | cAMP response element modulator | up |
| ccr-miR-17-5p | CAAAGTGCTTACAGTGCAGGTAG | down | comp16833_c0 | 3',5'-cyclic-nucleotide phosphodiesterase | down |
| hsa-miR-3618_1ss21GA | TGTCTACATTAATGAAAAGAAC | down | comp16833_c0 | 3',5'-cyclic-nucleotide phosphodiesterase | down |
| ssa-miR-16a-3p_R+1_2ss10TA11TC | CCAGTATTGACCGTGCTGCTGAA | down | comp16833_c0 | 3',5'-cyclic-nucleotide phosphodiesterase | down |
| ssa-miR-20a-5p | TAAAGTGCTTATAGTGCAGGTAG | down | comp16833_c0 | 3',5'-cyclic-nucleotide phosphodiesterase | down |
| dre-miR-27b-3p_R-1 | TTCACAGTGGCTAAGTTCTGC | up | comp16979_c0 | - | up |
| dre-miR-301c-3p_R+1 | CAGTGCAATAGTATTGTCATAGC | down | comp16979_c0 | - | up |
| ssa-miR-301a-3p | CAGTGCAATAGTATTGTCATAGC | down | comp16979_c0 | - | up |
| dre-miR-27b-3p_R-1 | TTCACAGTGGCTAAGTTCTGC | up | comp16985_c0 | 3',5'-cyclic-nucleotide phosphodiesterase | up |
| ssa-miR-20a-5p | TAAAGTGCTTATAGTGCAGGTAG | down | comp16985_c0 | 3',5'-cyclic-nucleotide phosphodiesterase | up |
| pma-miR-181a-3p | ACCATCGACCGTTGACTGTACC | up | comp17028_c0 | - | down |
| ssa-miR-16a-3p_R+1_2ss10TA11TC | CCAGTATTGACCGTGCTGCTGAA | down | comp17057_c0 | - | down |
| PC-5p-83983_9 | GCTTATTGTGAGTCCCGGGTA | down | comp17124_c0 | - | up |
| dre-miR-27b-3p_R-1 | TTCACAGTGGCTAAGTTCTGC | up | comp17124_c0 | - | up |
| dre-miR-301c-3p_R+1 | CAGTGCAATAGTATTGTCATAGC | down | comp17163_c0 | purine-nucleoside phosphorylase | up |
| ssa-miR-301a-3p | CAGTGCAATAGTATTGTCATAGC | down | comp17163_c0 | purine-nucleoside phosphorylase | up |
| ccr-miR-17-5p | CAAAGTGCTTACAGTGCAGGTAG | down | comp17169_c0 | - | up |
| dre-miR-27b-3p_R-1 | TTCACAGTGGCTAAGTTCTGC | up | comp17169_c0 | - | up |
| ssa-miR-20a-5p | TAAAGTGCTTATAGTGCAGGTAG | down | comp17169_c0 | - | up |
| ccr-miR-143_R+1_1ss20TA | TGAGATGAAGCACTGTAGCAC | up | comp17201_c0 | F-box and leucine-rich repeat protein 20 | up |
| dre-miR-301c-3p_R+1 | CAGTGCAATAGTATTGTCATAGC | down | comp17214_c0 | RUN and FYVE domain-containing protein 1 | up |
| ssa-miR-301a-3p | CAGTGCAATAGTATTGTCATAGC | down | comp17214_c0 | RUN and FYVE domain-containing protein 1 | up |
| PC-5p-48126_34 | CATGACTAGCCTCTCAAAGCAC | down | comp17281_c0 | death-associated protein kinase | down |
| dre-miR-27b-3p_R-1 | TTCACAGTGGCTAAGTTCTGC | up | comp17281_c0 | death-associated protein kinase | down |
| hsa-miR-3618_1ss21GA | TGTCTACATTAATGAAAAGAAC | down | comp17281_c0 | death-associated protein kinase | down |
| PC-5p-83983_9 | GCTTATTGTGAGTCCCGGGTA | down | comp17306_c1 | arylsulfatase I/J | up |
| ccr-miR-143_R+1_1ss20TA | TGAGATGAAGCACTGTAGCAC | up | comp17339_c0 | protein-tyrosine phosphatase | up |
| ssa-miR-16a-3p_R+1_2ss10TA11TC | CCAGTATTGACCGTGCTGCTGAA | down | comp17381_c0 | - | up |
| ccr-miR-143_R+1_1ss20TA | TGAGATGAAGCACTGTAGCAC | up | comp17415_c0 | ethanolamine-phosphate cytidylyltransferase | up |
| ssa-miR-16a-3p_R+1_2ss10TA11TC | CCAGTATTGACCGTGCTGCTGAA | down | comp17433_c0 | - | down |
| ccr-miR-17-5p | CAAAGTGCTTACAGTGCAGGTAG | down | comp17531_c0 | myosin I | down |
| ssa-miR-20a-5p | TAAAGTGCTTATAGTGCAGGTAG | down | comp17531_c0 | myosin I | down |
| ccr-miR-143_R+1_1ss20TA | TGAGATGAAGCACTGTAGCAC | up | comp17541_c1 | - | up |
| dre-miR-301c-3p_R+1 | CAGTGCAATAGTATTGTCATAGC | down | comp17601_c0 | - | up |
| ssa-miR-20a-5p | TAAAGTGCTTATAGTGCAGGTAG | down | comp17601_c0 | - | up |
| ssa-miR-301a-3p | CAGTGCAATAGTATTGTCATAGC | down | comp17601_c0 | - | up |
| PC-3p-36625_60 | TGCTTTGAGAGGCTAGTCATGA | down | comp17626_c0 | - | down |
| dre-miR-27b-3p_R-1 | TTCACAGTGGCTAAGTTCTGC | up | comp17626_c0 | - | down |
| ccr-miR-17-5p | CAAAGTGCTTACAGTGCAGGTAG | down | comp17629_c0 | cadherin 23 | up |
| dre-miR-27b-3p_R-1 | TTCACAGTGGCTAAGTTCTGC | up | comp17629_c0 | cadherin 23 | up |
| dre-miR-301c-3p_R+1 | CAGTGCAATAGTATTGTCATAGC | down | comp17629_c0 | cadherin 23 | up |
| ssa-miR-20a-5p | TAAAGTGCTTATAGTGCAGGTAG | down | comp17629_c0 | cadherin 23 | up |
| ssa-miR-301a-3p | CAGTGCAATAGTATTGTCATAGC | down | comp17629_c0 | cadherin 23 | up |
| dre-miR-27b-3p_R-1 | TTCACAGTGGCTAAGTTCTGC | up | comp17651_c0 | - | up |
| ccr-miR-143_R+1_1ss20TA | TGAGATGAAGCACTGTAGCAC | up | comp17724_c0 | - | down |
| dre-miR-338_R-1 | TCCAGCATCAGTGATTTTGTT | down | comp17724_c0 | - | down |
| ppy-miR-338-3p_R-1 | TCCAGCATCAGTGATTTTGTT | down | comp17724_c0 | - | down |
| hsa-miR-3618_1ss21GA | TGTCTACATTAATGAAAAGAAC | down | comp17809_c0 | myeloid/lymphoid or mixed-lineage leukemia protein 5 | up |
| ccr-miR-143_R+1_1ss20TA | TGAGATGAAGCACTGTAGCAC | up | comp17857_c0 | coproporphyrinogen III oxidase | up |
| dre-miR-338_R-1 | TCCAGCATCAGTGATTTTGTT | down | comp17891_c0 | - | down |
| ppy-miR-338-3p_R-1 | TCCAGCATCAGTGATTTTGTT | down | comp17891_c0 | - | down |
| dre-miR-27b-3p_R-1 | TTCACAGTGGCTAAGTTCTGC | up | comp17903_c0 | - | up |
| ssa-miR-16a-3p_R+1_2ss10TA11TC | CCAGTATTGACCGTGCTGCTGAA | down | comp17908_c0 | neurotransmitter:Na+ symporter, NSS family | up |
| dre-miR-338_R-1 | TCCAGCATCAGTGATTTTGTT | down | comp17989_c1 | thymidine phosphorylase | down |
| ppy-miR-338-3p_R-1 | TCCAGCATCAGTGATTTTGTT | down | comp17989_c1 | thymidine phosphorylase | down |
| dre-miR-27b-3p_R-1 | TTCACAGTGGCTAAGTTCTGC | up | comp18036_c0 | protein kinase N | up |
| dre-miR-27b-3p_R-1 | TTCACAGTGGCTAAGTTCTGC | up | comp18045_c0 | tumor necrosis factor receptor superfamily, member 10 | up |
| dre-miR-338_R-1 | TCCAGCATCAGTGATTTTGTT | down | comp18105_c0 | - | up |
| ppy-miR-338-3p_R-1 | TCCAGCATCAGTGATTTTGTT | down | comp18105_c0 | - | up |
| ssa-miR-16a-3p_R+1_2ss10TA11TC | CCAGTATTGACCGTGCTGCTGAA | down | comp18226_c0 | insulin receptor substrate | up |
| ccr-miR-143_R+1_1ss20TA | TGAGATGAAGCACTGTAGCAC | up | comp18265_c1 | monocyte to macrophage differentiation protein | up |
| ccr-miR-187 | TCGTGTCTTGTGTTGCAGCCAGT | up | comp18265_c1 | monocyte to macrophage differentiation protein | up |
| PC-3p-36625_60 | TGCTTTGAGAGGCTAGTCATGA | down | comp18285_c0 | - | up |
| PC-3p-36625_60 | TGCTTTGAGAGGCTAGTCATGA | down | comp18308_c1 | - | up |
| dre-miR-27b-3p_R-1 | TTCACAGTGGCTAAGTTCTGC | up | comp18308_c1 | - | up |
| ccr-miR-17-5p | CAAAGTGCTTACAGTGCAGGTAG | down | comp18342_c2 | mannosyl-oligosaccharide alpha-1,2-mannosidase | down |
| ssa-miR-20a-5p | TAAAGTGCTTATAGTGCAGGTAG | down | comp18342_c2 | mannosyl-oligosaccharide alpha-1,2-mannosidase | down |
| PC-3p-36625_60 | TGCTTTGAGAGGCTAGTCATGA | down | comp18390_c0 | mitochondrial carrier protein, MC family | up |
| dre-miR-338_R-1 | TCCAGCATCAGTGATTTTGTT | down | comp18391_c0 | protein-serine/threonine kinase | up |
| ppy-miR-338-3p_R-1 | TCCAGCATCAGTGATTTTGTT | down | comp18391_c0 | protein-serine/threonine kinase | up |
| ssa-miR-16a-3p_R+1_2ss10TA11TC | CCAGTATTGACCGTGCTGCTGAA | down | comp18391_c0 | protein-serine/threonine kinase | up |
| PC-5p-83983_9 | GCTTATTGTGAGTCCCGGGTA | down | comp18432_c0 | Ras homolog gene family, member U | down |
| dre-miR-27b-3p_R-1 | TTCACAGTGGCTAAGTTCTGC | up | comp18432_c0 | Ras homolog gene family, member U | down |
| ssa-miR-200b-5p | CATCTTACCTGACAGTGCTGGA | up | comp18432_c0 | Ras homolog gene family, member U | down |
| PC-3p-36625_60 | TGCTTTGAGAGGCTAGTCATGA | down | comp18458_c0 | - | down |
| dre-miR-27b-3p_R-1 | TTCACAGTGGCTAAGTTCTGC | up | comp18460_c0 | transglutaminase 2 | down |
| hsa-miR-3618_1ss21GA | TGTCTACATTAATGAAAAGAAC | down | comp18460_c0 | transglutaminase 2 | down |
| ccr-miR-143_R+1_1ss20TA | TGAGATGAAGCACTGTAGCAC | up | comp18470_c0 | fos-like antigen 2 | up |
| ccr-miR-143_R+1_1ss20TA | TGAGATGAAGCACTGTAGCAC | up | comp18509_c0 | activating transcription factor 2 | up |
| ccr-miR-17-5p | CAAAGTGCTTACAGTGCAGGTAG | down | comp18509_c0 | activating transcription factor 2 | up |
| ssa-miR-20a-5p | TAAAGTGCTTATAGTGCAGGTAG | down | comp18509_c0 | activating transcription factor 2 | up |
| PC-5p-83983_9 | GCTTATTGTGAGTCCCGGGTA | down | comp18641_c1 | NUAK family, SNF1-like kinase | up |
| dre-miR-27b-3p_R-1 | TTCACAGTGGCTAAGTTCTGC | up | comp18665_c0 | lipoprotein lipase | down |
| ssa-miR-16a-3p_R+1_2ss10TA11TC | CCAGTATTGACCGTGCTGCTGAA | down | comp18665_c0 | lipoprotein lipase | down |
| ssa-miR-200b-5p | CATCTTACCTGACAGTGCTGGA | up | comp18715_c0 | RAD54-like protein 2 | up |
| ccr-miR-17-5p | CAAAGTGCTTACAGTGCAGGTAG | down | comp18721_c0 | alcohol sulfotransferase | down |
| dre-miR-338_R-1 | TCCAGCATCAGTGATTTTGTT | down | comp18721_c0 | alcohol sulfotransferase | down |
| ppy-miR-338-3p_R-1 | TCCAGCATCAGTGATTTTGTT | down | comp18721_c0 | alcohol sulfotransferase | down |
| ssa-miR-20a-5p | TAAAGTGCTTATAGTGCAGGTAG | down | comp18721_c0 | alcohol sulfotransferase | down |
| dre-miR-27b-3p_R-1 | TTCACAGTGGCTAAGTTCTGC | up | comp18749_c0 | - | up |
| dre-miR-193b-3p | AACTGGCCCGCAAAGTCCCGCT | up | comp18935_c1 | erythrocyte membrane protein band 4.1 | up |
| ssa-miR-16a-3p_R+1_2ss10TA11TC | CCAGTATTGACCGTGCTGCTGAA | down | comp18992_c2 | - | up |
| dre-miR-301c-3p_R+1 | CAGTGCAATAGTATTGTCATAGC | down | comp19049_c0 | - | up |
| dre-miR-338_R-1 | TCCAGCATCAGTGATTTTGTT | down | comp19049_c0 | - | up |
| pma-miR-181a-3p | ACCATCGACCGTTGACTGTACC | up | comp19049_c0 | - | up |
| ppy-miR-338-3p_R-1 | TCCAGCATCAGTGATTTTGTT | down | comp19049_c0 | - | up |
| ssa-miR-301a-3p | CAGTGCAATAGTATTGTCATAGC | down | comp19049_c0 | - | up |
| dre-miR-27b-3p_R-1 | TTCACAGTGGCTAAGTTCTGC | up | comp19098_c1 | - | up |
| ssa-miR-16a-3p_R+1_2ss10TA11TC | CCAGTATTGACCGTGCTGCTGAA | down | comp19098_c1 | - | up |
| PC-5p-83983_9 | GCTTATTGTGAGTCCCGGGTA | down | comp19104_c0 | lipoprotein lipase | down |
| ccr-miR-17-5p | CAAAGTGCTTACAGTGCAGGTAG | down | comp19104_c0 | lipoprotein lipase | down |
| dre-miR-301c-3p_R+1 | CAGTGCAATAGTATTGTCATAGC | down | comp19104_c0 | lipoprotein lipase | down |
| ssa-miR-20a-5p | TAAAGTGCTTATAGTGCAGGTAG | down | comp19104_c0 | lipoprotein lipase | down |
| ssa-miR-301a-3p | CAGTGCAATAGTATTGTCATAGC | down | comp19104_c0 | lipoprotein lipase | down |
| dre-miR-27b-3p_R-1 | TTCACAGTGGCTAAGTTCTGC | up | comp19211_c0 | hexokinase | up |
| hsa-miR-3618_1ss21GA | TGTCTACATTAATGAAAAGAAC | down | comp19211_c0 | hexokinase | up |
| pma-miR-181a-3p | ACCATCGACCGTTGACTGTACC | up | comp19211_c0 | hexokinase | up |
| ssa-miR-200b-5p | CATCTTACCTGACAGTGCTGGA | up | comp19211_c0 | hexokinase | up |
| hsa-miR-3618_1ss21GA | TGTCTACATTAATGAAAAGAAC | down | comp19218_c0 | MFS transporter, SP family, solute carrier family 2 (facilitated glucose transporter), member 1 | up |
| dre-miR-193b-3p | AACTGGCCCGCAAAGTCCCGCT | up | comp19255_c0 | - | down |
| ccr-miR-17-5p | CAAAGTGCTTACAGTGCAGGTAG | down | comp192759_c0 | transcription factor AP-4 | down |
| ssa-miR-20a-5p | TAAAGTGCTTATAGTGCAGGTAG | down | comp192759_c0 | transcription factor AP-4 | down |
| ssa-miR-16a-3p_R+1_2ss10TA11TC | CCAGTATTGACCGTGCTGCTGAA | down | comp19300_c0 | - | up |
| ssa-miR-16a-3p_R+1_2ss10TA11TC | CCAGTATTGACCGTGCTGCTGAA | down | comp19394_c0 | transient receptor potential cation channel, subfamily C, member 6 | up |
| ssa-miR-16a-3p_R+1_2ss10TA11TC | CCAGTATTGACCGTGCTGCTGAA | down | comp19397_c1 | class B basic helix-loop-helix protein 3 | up |
| ccr-miR-17-5p | CAAAGTGCTTACAGTGCAGGTAG | down | comp19400_c0 | polycomb group RING finger protein 1 | up |
| ssa-miR-20a-5p | TAAAGTGCTTATAGTGCAGGTAG | down | comp19400_c0 | polycomb group RING finger protein 1 | up |
| pma-miR-181a-3p | ACCATCGACCGTTGACTGTACC | up | comp19468_c0 | - | down |
| dre-miR-27b-3p_R-1 | TTCACAGTGGCTAAGTTCTGC | up | comp19485_c0 | - | up |
| ccr-miR-17-5p | CAAAGTGCTTACAGTGCAGGTAG | down | comp19528_c1 | 6-phosphofructokinase | up |
| dre-miR-27b-3p_R-1 | TTCACAGTGGCTAAGTTCTGC | up | comp19528_c1 | 6-phosphofructokinase | up |
| dre-miR-301c-3p_R+1 | CAGTGCAATAGTATTGTCATAGC | down | comp19528_c1 | 6-phosphofructokinase | up |
| ssa-miR-20a-5p | TAAAGTGCTTATAGTGCAGGTAG | down | comp19528_c1 | 6-phosphofructokinase | up |
| ssa-miR-301a-3p | CAGTGCAATAGTATTGTCATAGC | down | comp19528_c1 | 6-phosphofructokinase | up |
| dre-miR-27b-3p_R-1 | TTCACAGTGGCTAAGTTCTGC | up | comp19552_c0 | Cu2+-exporting ATPase | up |
| PC-3p-36625_60 | TGCTTTGAGAGGCTAGTCATGA | down | comp19575_c0 | Rab11 family-interacting protein 3/4 | up |
| ccr-miR-143_R+1_1ss20TA | TGAGATGAAGCACTGTAGCAC | up | comp19575_c0 | Rab11 family-interacting protein 3/4 | up |
| dre-miR-338_R-1 | TCCAGCATCAGTGATTTTGTT | down | comp19639_c0 | - | up |
| ppy-miR-338-3p_R-1 | TCCAGCATCAGTGATTTTGTT | down | comp19639_c0 | - | up |
| ccr-miR-143_R+1_1ss20TA | TGAGATGAAGCACTGTAGCAC | up | comp19753_c1 | 5'-AMP-activated protein kinase, regulatory gamma subunit | up |
| dre-miR-193b-3p | AACTGGCCCGCAAAGTCCCGCT | up | comp19810_c0 | bile acid-CoA:amino acid N-acyltransferase | up |
| ccr-miR-143_R+1_1ss20TA | TGAGATGAAGCACTGTAGCAC | up | comp19837_c0 | nuclear receptor, subfamily 1, group C, member 1 | up |
| ccr-miR-17-5p | CAAAGTGCTTACAGTGCAGGTAG | down | comp19837_c0 | nuclear receptor, subfamily 1, group C, member 1 | up |
| ola-miR-210-5p_R+2_1ss20TC | AGCCACTGACTAACGCACACTG | up | comp19837_c0 | nuclear receptor, subfamily 1, group C, member 1 | up |
| ssa-miR-20a-5p | TAAAGTGCTTATAGTGCAGGTAG | down | comp19837_c0 | nuclear receptor, subfamily 1, group C, member 1 | up |
| ssa-miR-16a-3p_R+1_2ss10TA11TC | CCAGTATTGACCGTGCTGCTGAA | down | comp19840_c0 | transferrin receptor | up |
| ssa-miR-20a-5p | TAAAGTGCTTATAGTGCAGGTAG | down | comp19840_c0 | transferrin receptor | up |
| ola-miR-210-5p_R+2_1ss20TC | AGCCACTGACTAACGCACACTG | up | comp19862_c0 | - | up |
| ssa-miR-200b-5p | CATCTTACCTGACAGTGCTGGA | up | comp19895_c0 | receptor tyrosine-protein kinase erbB-3 | down |
| dre-miR-301c-3p_R+1 | CAGTGCAATAGTATTGTCATAGC | down | comp19946_c2 | - | up |
| ssa-miR-301a-3p | CAGTGCAATAGTATTGTCATAGC | down | comp19946_c2 | - | up |
| ccr-miR-17-5p | CAAAGTGCTTACAGTGCAGGTAG | down | comp19983_c0 | myosin heavy chain | up |
| dre-miR-27b-3p_R-1 | TTCACAGTGGCTAAGTTCTGC | up | comp19983_c0 | myosin heavy chain | up |
| ssa-miR-20a-5p | TAAAGTGCTTATAGTGCAGGTAG | down | comp19983_c0 | myosin heavy chain | up |
| ccr-miR-187 | TCGTGTCTTGTGTTGCAGCCAGT | up | comp20010_c0 | - | up |
| ola-miR-210-5p_R+2_1ss20TC | AGCCACTGACTAACGCACACTG | up | comp20010_c0 | - | up |
| ssa-miR-16a-3p_R+1_2ss10TA11TC | CCAGTATTGACCGTGCTGCTGAA | down | comp20010_c0 | - | up |
| ssa-miR-200b-5p | CATCTTACCTGACAGTGCTGGA | up | comp20010_c0 | - | up |
| dre-miR-27b-3p_R-1 | TTCACAGTGGCTAAGTTCTGC | up | comp20020_c1 | - | up |
| PC-5p-48126_34 | CATGACTAGCCTCTCAAAGCAC | down | comp20056_c0 | inorganic phosphate transporter, PiT family | up |
| dre-miR-301c-3p_R+1 | CAGTGCAATAGTATTGTCATAGC | down | comp20056_c0 | inorganic phosphate transporter, PiT family | up |
| dre-miR-338_R-1 | TCCAGCATCAGTGATTTTGTT | down | comp20056_c0 | inorganic phosphate transporter, PiT family | up |
| ppy-miR-338-3p_R-1 | TCCAGCATCAGTGATTTTGTT | down | comp20056_c0 | inorganic phosphate transporter, PiT family | up |
| ssa-miR-301a-3p | CAGTGCAATAGTATTGTCATAGC | down | comp20056_c0 | inorganic phosphate transporter, PiT family | up |
| hsa-miR-3618_1ss21GA | TGTCTACATTAATGAAAAGAAC | down | comp21298_c0 | - | up |
| ccr-miR-143_R+1_1ss20TA | TGAGATGAAGCACTGTAGCAC | up | comp2836_c0 | - | down |
| ssa-miR-200b-5p | CATCTTACCTGACAGTGCTGGA | up | comp2836_c0 | - | down |
| ssa-miR-20a-5p | TAAAGTGCTTATAGTGCAGGTAG | down | comp307943_c0 | SCAN domain-containing zinc finger protein | down |
| ccr-miR-187 | TCGTGTCTTGTGTTGCAGCCAGT | up | comp3100_c0 | - | down |
| ssa-miR-16a-3p_R+1_2ss10TA11TC | CCAGTATTGACCGTGCTGCTGAA | down | comp3109_c0 | - | down |
| PC-5p-48126_34 | CATGACTAGCCTCTCAAAGCAC | down | comp3405_c0 | replication factor A2 | down |
| ccr-miR-17-5p | CAAAGTGCTTACAGTGCAGGTAG | down | comp3678_c0 | - | down |
| ssa-miR-20a-5p | TAAAGTGCTTATAGTGCAGGTAG | down | comp3678_c0 | - | down |
| ccr-miR-17-5p | CAAAGTGCTTACAGTGCAGGTAG | down | comp3830_c0 | - | down |
| ssa-miR-20a-5p | TAAAGTGCTTATAGTGCAGGTAG | down | comp3830_c0 | - | down |
| dre-miR-27b-3p_R-1 | TTCACAGTGGCTAAGTTCTGC | up | comp4127_c0 | myeloid leukemia cell differntiation protein MCL-1 | up |
| dre-miR-27b-3p_R-1 | TTCACAGTGGCTAAGTTCTGC | up | comp47219_c0 | - | up |
| ccr-miR-17-5p | CAAAGTGCTTACAGTGCAGGTAG | down | comp5908_c0 | L-lactate dehydrogenase | up |
| ssa-miR-20a-5p | TAAAGTGCTTATAGTGCAGGTAG | down | comp5908_c0 | L-lactate dehydrogenase | up |
| ssa-miR-16a-3p_R+1_2ss10TA11TC | CCAGTATTGACCGTGCTGCTGAA | down | comp7338_c0 | - | down |
| dre-miR-27b-3p_R-1 | TTCACAGTGGCTAAGTTCTGC | up | comp7480_c0 | aldehyde reductase | down |
| ccr-miR-143_R+1_1ss20TA | TGAGATGAAGCACTGTAGCAC | up | comp7530_c0 | zinc finger and BTB domain-containing protein 16 | up |
| ccr-miR-17-5p | CAAAGTGCTTACAGTGCAGGTAG | down | comp7530_c0 | zinc finger and BTB domain-containing protein 16 | up |
| ssa-miR-20a-5p | TAAAGTGCTTATAGTGCAGGTAG | down | comp7530_c0 | zinc finger and BTB domain-containing protein 16 | up |
| dre-miR-27b-3p_R-1 | TTCACAGTGGCTAAGTTCTGC | up | comp7697_c0 | histone H2A | down |
| ccr-miR-17-5p | CAAAGTGCTTACAGTGCAGGTAG | down | comp7721_c0 | angiopoietin-like 4 | up |
| ssa-miR-20a-5p | TAAAGTGCTTATAGTGCAGGTAG | down | comp7721_c0 | angiopoietin-like 4 | up |
| ssa-miR-16a-3p_R+1_2ss10TA11TC | CCAGTATTGACCGTGCTGCTGAA | down | comp8016_c0 | - | up |
| PC-3p-36625_60 | TGCTTTGAGAGGCTAGTCATGA | down | comp8203_c0 | - | up |
| ccr-miR-17-5p | CAAAGTGCTTACAGTGCAGGTAG | down | comp8306_c0 | kinetochore-associated protein 1 | down |
| dre-miR-301c-3p_R+1 | CAGTGCAATAGTATTGTCATAGC | down | comp8306_c0 | kinetochore-associated protein 1 | down |
| ssa-miR-20a-5p | TAAAGTGCTTATAGTGCAGGTAG | down | comp8306_c0 | kinetochore-associated protein 1 | down |
| ssa-miR-301a-3p | CAGTGCAATAGTATTGTCATAGC | down | comp8306_c0 | kinetochore-associated protein 1 | down |
| PC-3p-36625_60 | TGCTTTGAGAGGCTAGTCATGA | down | comp8360_c0 | - | down |
| dre-miR-193b-3p | AACTGGCCCGCAAAGTCCCGCT | up | comp85454_c0 | solute carrier family 1 (neutral amino acid transporter), member 5 | up |
| dre-miR-338_R-1 | TCCAGCATCAGTGATTTTGTT | down | comp8626_c0 | - | up |
| ppy-miR-338-3p_R-1 | TCCAGCATCAGTGATTTTGTT | down | comp8626_c0 | - | up |
| dre-miR-27b-3p_R-1 | TTCACAGTGGCTAAGTTCTGC | up | comp9504_c0 | purinergic receptor P2Y, G protein-coupled, 5 | down |
| dre-miR-27b-3p_R-1 | TTCACAGTGGCTAAGTTCTGC | up | comp9770_c0 | transcription factor AP-1 | up |
| dre-miR-301c-3p_R+1 | CAGTGCAATAGTATTGTCATAGC | down | comp9770_c0 | transcription factor AP-1 | up |
| ssa-miR-301a-3p | CAGTGCAATAGTATTGTCATAGC | down | comp9770_c0 | transcription factor AP-1 | up |
| dre-miR-27b-3p_R-1 | TTCACAGTGGCTAAGTTCTGC | up | comp9860_c1 | - | down |
| dre-miR-27b-3p_R-1 | TTCACAGTGGCTAAGTTCTGC | up | comp9877_c0 | histamine N-methyltransferase | down |
| ssa-miR-16a-3p_R+1_2ss10TA11TC | CCAGTATTGACCGTGCTGCTGAA | down | comp9951_c0 | - | up |

Table S10 List of miRNA-mRNA pairs with negative correlation.

| miR_name | miR_seq | regulation | Accession | annotation | regulation |
| --- | --- | --- | --- | --- | --- |
| ssa-miR-20a-5p | TAAAGTGCTTATAGTGCAGGTAG | down | comp10260_c0 | - | up |
| PC-5p-83983_9 | GCTTATTGTGAGTCCCGGGTA | down | comp10699_c0 | - | up |
| ccr-miR-17-5p | CAAAGTGCTTACAGTGCAGGTAG | down | comp10699_c2 | - | up |
| ssa-miR-20a-5p | TAAAGTGCTTATAGTGCAGGTAG | down | comp10699_c2 | - | up |
| hsa-miR-3618_1ss21GA | TGTCTACATTAATGAAAAGAAC | down | comp11189_c0 | - | up |
| PC-3p-36625_60 | TGCTTTGAGAGGCTAGTCATGA | down | comp11248_c0 | methylsterol monooxygenase | up |
| ccr-miR-17-5p | CAAAGTGCTTACAGTGCAGGTAG | down | comp11248_c0 | methylsterol monooxygenase | up |
| ssa-miR-20a-5p | TAAAGTGCTTATAGTGCAGGTAG | down | comp11248_c0 | methylsterol monooxygenase | up |
| ccr-miR-143_R+1_1ss20TA | TGAGATGAAGCACTGTAGCAC | up | comp11294_c0 | layilin | down |
| ssa-miR-16a-3p_R+1_2ss10TA11TC | CCAGTATTGACCGTGCTGCTGAA | down | comp11803_c0 | fatty acid desaturase 2 (delta-6 desaturase) | up |
| PC-3p-36625_60 | TGCTTTGAGAGGCTAGTCATGA | down | comp11888_c0 | protein phosphatase 3, regulatory subunit | up |
| PC-5p-83983_9 | GCTTATTGTGAGTCCCGGGTA | down | comp12230_c0 | carbonic anhydrase | up |
| PC-5p-83983_9 | GCTTATTGTGAGTCCCGGGTA | down | comp12233_c0 | myosin IX | up |
| ssa-miR-16a-3p_R+1_2ss10TA11TC | CCAGTATTGACCGTGCTGCTGAA | down | comp12317_c0 | diphosphoinositol-polyphosphate diphosphatase | up |
| PC-3p-36625_60 | TGCTTTGAGAGGCTAGTCATGA | down | comp12482_c0 | apoptosis regulator BCL-2 | up |
| dre-miR-27b-3p_R-1 | TTCACAGTGGCTAAGTTCTGC | up | comp12539_c1 | - | down |
| PC-5p-83983_9 | GCTTATTGTGAGTCCCGGGTA | down | comp12673_c0 | - | up |
| dre-miR-301c-3p_R+1 | CAGTGCAATAGTATTGTCATAGC | down | comp12753_c0 | - | up |
| ssa-miR-301a-3p | CAGTGCAATAGTATTGTCATAGC | down | comp12753_c0 | - | up |
| PC-5p-48126_34 | CATGACTAGCCTCTCAAAGCAC | down | comp12927_c0 | Ras-related protein Rab-20 | up |
| dre-miR-301c-3p_R+1 | CAGTGCAATAGTATTGTCATAGC | down | comp13235_c0 | - | up |
| ssa-miR-301a-3p | CAGTGCAATAGTATTGTCATAGC | down | comp13235_c0 | - | up |
| ssa-miR-16a-3p_R+1_2ss10TA11TC | CCAGTATTGACCGTGCTGCTGAA | down | comp13255_c1 | bromodomain-containing protein 4 | up |
| ccr-miR-17-5p | CAAAGTGCTTACAGTGCAGGTAG | down | comp13563_c0 | mitochondrial brown fat uncoupling protein 1 | up |
| ssa-miR-20a-5p | TAAAGTGCTTATAGTGCAGGTAG | down | comp13563_c0 | mitochondrial brown fat uncoupling protein 1 | up |
| PC-3p-36625_60 | TGCTTTGAGAGGCTAGTCATGA | down | comp13907_c0 | angiopoietin-like 4 | up |
| ccr-miR-17-5p | CAAAGTGCTTACAGTGCAGGTAG | down | comp13915_c0 | phosphoglycerate mutase | up |
| ssa-miR-20a-5p | TAAAGTGCTTATAGTGCAGGTAG | down | comp13915_c0 | phosphoglycerate mutase | up |
| ccr-miR-143_R+1_1ss20TA | TGAGATGAAGCACTGTAGCAC | up | comp13969_c0 | - | down |
| ssa-miR-16a-3p_R+1_2ss10TA11TC | CCAGTATTGACCGTGCTGCTGAA | down | comp14343_c0 | cysteamine dioxygenase | up |
| ccr-miR-143_R+1_1ss20TA | TGAGATGAAGCACTGTAGCAC | up | comp14388_c0 | - | down |
| dre-miR-301c-3p_R+1 | CAGTGCAATAGTATTGTCATAGC | down | comp14576_c0 | protein-tyrosine phosphatase | up |
| ssa-miR-301a-3p | CAGTGCAATAGTATTGTCATAGC | down | comp14576_c0 | protein-tyrosine phosphatase | up |
| PC-5p-48126_34 | CATGACTAGCCTCTCAAAGCAC | down | comp14617_c0 | - | up |
| ssa-miR-16a-3p_R+1_2ss10TA11TC | CCAGTATTGACCGTGCTGCTGAA | down | comp14617_c0 | - | up |
| dre-miR-301c-3p_R+1 | CAGTGCAATAGTATTGTCATAGC | down | comp14797_c0 | MFS transporter, MCP family, solute carrier family 16 (monocarboxylic acid transporters), member 3 | up |
| ssa-miR-301a-3p | CAGTGCAATAGTATTGTCATAGC | down | comp14797_c0 | MFS transporter, MCP family, solute carrier family 16 (monocarboxylic acid transporters), member 3 | up |
| dre-miR-338_R-1 | TCCAGCATCAGTGATTTTGTT | down | comp14901_c0 | LIM domain kinase 2 | up |
| ppy-miR-338-3p_R-1 | TCCAGCATCAGTGATTTTGTT | down | comp14901_c0 | LIM domain kinase 2 | up |
| dre-miR-27b-3p_R-1 | TTCACAGTGGCTAAGTTCTGC | up | comp14960_c0 | peptidyl-prolyl isomerase H (cyclophilin H) | down |
| ola-miR-210-5p_R+2_1ss20TC | AGCCACTGACTAACGCACACTG | up | comp14976_c0 | - | down |
| ccr-miR-17-5p | CAAAGTGCTTACAGTGCAGGTAG | down | comp15311_c0 | TNF receptor-associated factor 4 | up |
| ssa-miR-20a-5p | TAAAGTGCTTATAGTGCAGGTAG | down | comp15311_c0 | TNF receptor-associated factor 4 | up |
| dre-miR-301c-3p_R+1 | CAGTGCAATAGTATTGTCATAGC | down | comp15330_c2 | - | up |
| ssa-miR-301a-3p | CAGTGCAATAGTATTGTCATAGC | down | comp15330_c2 | - | up |
| dre-miR-27b-3p_R-1 | TTCACAGTGGCTAAGTTCTGC | up | comp15594_c0 | 3',5'-cyclic-nucleotide phosphodiesterase | down |
| ola-miR-210-5p_R+2_1ss20TC | AGCCACTGACTAACGCACACTG | up | comp15700_c0 | - | down |
| ssa-miR-16a-3p_R+1_2ss10TA11TC | CCAGTATTGACCGTGCTGCTGAA | down | comp15877_c0 | glycogen(starch) synthase | up |
| ccr-miR-143_R+1_1ss20TA | TGAGATGAAGCACTGTAGCAC | up | comp16016_c1 | - | down |
| dre-miR-338_R-1 | TCCAGCATCAGTGATTTTGTT | down | comp16131_c0 | von Hippel-Lindau disease tumor supressor | up |
| ppy-miR-338-3p_R-1 | TCCAGCATCAGTGATTTTGTT | down | comp16131_c0 | von Hippel-Lindau disease tumor supressor | up |
| ccr-miR-17-5p | CAAAGTGCTTACAGTGCAGGTAG | down | comp16210_c0 | - | up |
| dre-miR-301c-3p_R+1 | CAGTGCAATAGTATTGTCATAGC | down | comp16210_c0 | - | up |
| ssa-miR-20a-5p | TAAAGTGCTTATAGTGCAGGTAG | down | comp16210_c0 | - | up |
| ssa-miR-301a-3p | CAGTGCAATAGTATTGTCATAGC | down | comp16210_c0 | - | up |
| PC-5p-83983_9 | GCTTATTGTGAGTCCCGGGTA | down | comp16219_c0 | histone demethylase JMJD3 | up |
| dre-miR-338_R-1 | TCCAGCATCAGTGATTTTGTT | down | comp16342_c1 | shingomyelin synthase | up |
| ppy-miR-338-3p_R-1 | TCCAGCATCAGTGATTTTGTT | down | comp16342_c1 | shingomyelin synthase | up |
| ccr-miR-17-5p | CAAAGTGCTTACAGTGCAGGTAG | down | comp16360_c0 | dual specificity phosphatase | up |
| PC-3p-36625_60 | TGCTTTGAGAGGCTAGTCATGA | down | comp16469_c0 | - | up |
| ccr-miR-17-5p | CAAAGTGCTTACAGTGCAGGTAG | down | comp16593_c0 | cAMP response element modulator | up |
| ssa-miR-20a-5p | TAAAGTGCTTATAGTGCAGGTAG | down | comp16593_c0 | cAMP response element modulator | up |
| dre-miR-301c-3p_R+1 | CAGTGCAATAGTATTGTCATAGC | down | comp16979_c0 | - | up |
| ssa-miR-301a-3p | CAGTGCAATAGTATTGTCATAGC | down | comp16979_c0 | - | up |
| ssa-miR-20a-5p | TAAAGTGCTTATAGTGCAGGTAG | down | comp16985_c0 | 3',5'-cyclic-nucleotide phosphodiesterase | up |
| pma-miR-181a-3p | ACCATCGACCGTTGACTGTACC | up | comp17028_c0 | - | down |
| PC-5p-83983_9 | GCTTATTGTGAGTCCCGGGTA | down | comp17124_c0 | - | up |
| dre-miR-301c-3p_R+1 | CAGTGCAATAGTATTGTCATAGC | down | comp17163_c0 | purine-nucleoside phosphorylase | up |
| ssa-miR-301a-3p | CAGTGCAATAGTATTGTCATAGC | down | comp17163_c0 | purine-nucleoside phosphorylase | up |
| ccr-miR-17-5p | CAAAGTGCTTACAGTGCAGGTAG | down | comp17169_c0 | - | up |
| ssa-miR-20a-5p | TAAAGTGCTTATAGTGCAGGTAG | down | comp17169_c0 | - | up |
| dre-miR-301c-3p_R+1 | CAGTGCAATAGTATTGTCATAGC | down | comp17214_c0 | RUN and FYVE domain-containing protein 1 | up |
| ssa-miR-301a-3p | CAGTGCAATAGTATTGTCATAGC | down | comp17214_c0 | RUN and FYVE domain-containing protein 1 | up |
| dre-miR-27b-3p_R-1 | TTCACAGTGGCTAAGTTCTGC | up | comp17281_c0 | death-associated protein kinase | down |
| PC-5p-83983_9 | GCTTATTGTGAGTCCCGGGTA | down | comp17306_c1 | arylsulfatase I/J | up |
| ssa-miR-16a-3p_R+1_2ss10TA11TC | CCAGTATTGACCGTGCTGCTGAA | down | comp17381_c0 | - | up |
| dre-miR-301c-3p_R+1 | CAGTGCAATAGTATTGTCATAGC | down | comp17601_c0 | - | up |
| ssa-miR-20a-5p | TAAAGTGCTTATAGTGCAGGTAG | down | comp17601_c0 | - | up |
| ssa-miR-301a-3p | CAGTGCAATAGTATTGTCATAGC | down | comp17601_c0 | - | up |
| dre-miR-27b-3p_R-1 | TTCACAGTGGCTAAGTTCTGC | up | comp17626_c0 | - | down |
| ccr-miR-17-5p | CAAAGTGCTTACAGTGCAGGTAG | down | comp17629_c0 | cadherin 23 | up |
| dre-miR-301c-3p_R+1 | CAGTGCAATAGTATTGTCATAGC | down | comp17629_c0 | cadherin 23 | up |
| ssa-miR-20a-5p | TAAAGTGCTTATAGTGCAGGTAG | down | comp17629_c0 | cadherin 23 | up |
| ssa-miR-301a-3p | CAGTGCAATAGTATTGTCATAGC | down | comp17629_c0 | cadherin 23 | up |
| ccr-miR-143_R+1_1ss20TA | TGAGATGAAGCACTGTAGCAC | up | comp17724_c0 | - | down |
| hsa-miR-3618_1ss21GA | TGTCTACATTAATGAAAAGAAC | down | comp17809_c0 | myeloid/lymphoid or mixed-lineage leukemia protein 5 | up |
| ssa-miR-16a-3p_R+1_2ss10TA11TC | CCAGTATTGACCGTGCTGCTGAA | down | comp17908_c0 | neurotransmitter:Na+ symporter, NSS family | up |
| dre-miR-338_R-1 | TCCAGCATCAGTGATTTTGTT | down | comp18105_c0 | - | up |
| ppy-miR-338-3p_R-1 | TCCAGCATCAGTGATTTTGTT | down | comp18105_c0 | - | up |
| ssa-miR-16a-3p_R+1_2ss10TA11TC | CCAGTATTGACCGTGCTGCTGAA | down | comp18226_c0 | insulin receptor substrate | up |
| PC-3p-36625_60 | TGCTTTGAGAGGCTAGTCATGA | down | comp18285_c0 | - | up |
| PC-3p-36625_60 | TGCTTTGAGAGGCTAGTCATGA | down | comp18308_c1 | - | up |
| PC-3p-36625_60 | TGCTTTGAGAGGCTAGTCATGA | down | comp18390_c0 | mitochondrial carrier protein, MC family | up |
| dre-miR-338_R-1 | TCCAGCATCAGTGATTTTGTT | down | comp18391_c0 | protein-serine/threonine kinase | up |
| ppy-miR-338-3p_R-1 | TCCAGCATCAGTGATTTTGTT | down | comp18391_c0 | protein-serine/threonine kinase | up |
| ssa-miR-16a-3p_R+1_2ss10TA11TC | CCAGTATTGACCGTGCTGCTGAA | down | comp18391_c0 | protein-serine/threonine kinase | up |
| dre-miR-27b-3p_R-1 | TTCACAGTGGCTAAGTTCTGC | up | comp18432_c0 | Ras homolog gene family, member U | down |
| ssa-miR-200b-5p | CATCTTACCTGACAGTGCTGGA | up | comp18432_c0 | Ras homolog gene family, member U | down |
| dre-miR-27b-3p_R-1 | TTCACAGTGGCTAAGTTCTGC | up | comp18460_c0 | transglutaminase 2 | down |
| ccr-miR-17-5p | CAAAGTGCTTACAGTGCAGGTAG | down | comp18509_c0 | activating transcription factor 2 | up |
| ssa-miR-20a-5p | TAAAGTGCTTATAGTGCAGGTAG | down | comp18509_c0 | activating transcription factor 2 | up |
| PC-5p-83983_9 | GCTTATTGTGAGTCCCGGGTA | down | comp18641_c1 | NUAK family, SNF1-like kinase | up |
| dre-miR-27b-3p_R-1 | TTCACAGTGGCTAAGTTCTGC | up | comp18665_c0 | lipoprotein lipase | down |
| ssa-miR-16a-3p_R+1_2ss10TA11TC | CCAGTATTGACCGTGCTGCTGAA | down | comp18992_c2 | - | up |
| dre-miR-301c-3p_R+1 | CAGTGCAATAGTATTGTCATAGC | down | comp19049_c0 | - | up |
| dre-miR-338_R-1 | TCCAGCATCAGTGATTTTGTT | down | comp19049_c0 | - | up |
| ppy-miR-338-3p_R-1 | TCCAGCATCAGTGATTTTGTT | down | comp19049_c0 | - | up |
| ssa-miR-301a-3p | CAGTGCAATAGTATTGTCATAGC | down | comp19049_c0 | - | up |
| ssa-miR-16a-3p_R+1_2ss10TA11TC | CCAGTATTGACCGTGCTGCTGAA | down | comp19098_c1 | - | up |
| hsa-miR-3618_1ss21GA | TGTCTACATTAATGAAAAGAAC | down | comp19211_c0 | hexokinase | up |
| hsa-miR-3618_1ss21GA | TGTCTACATTAATGAAAAGAAC | down | comp19218_c0 | MFS transporter, SP family, solute carrier family 2 (facilitated glucose transporter), member 1 | up |
| dre-miR-193b-3p | AACTGGCCCGCAAAGTCCCGCT | up | comp19255_c0 | - | down |
| ssa-miR-16a-3p_R+1_2ss10TA11TC | CCAGTATTGACCGTGCTGCTGAA | down | comp19300_c0 | - | up |
| ssa-miR-16a-3p_R+1_2ss10TA11TC | CCAGTATTGACCGTGCTGCTGAA | down | comp19394_c0 | transient receptor potential cation channel, subfamily C, member 6 | up |
| ssa-miR-16a-3p_R+1_2ss10TA11TC | CCAGTATTGACCGTGCTGCTGAA | down | comp19397_c1 | class B basic helix-loop-helix protein 3 | up |
| ccr-miR-17-5p | CAAAGTGCTTACAGTGCAGGTAG | down | comp19400_c0 | polycomb group RING finger protein 1 | up |
| ssa-miR-20a-5p | TAAAGTGCTTATAGTGCAGGTAG | down | comp19400_c0 | polycomb group RING finger protein 1 | up |
| pma-miR-181a-3p | ACCATCGACCGTTGACTGTACC | up | comp19468_c0 | - | down |
| ccr-miR-17-5p | CAAAGTGCTTACAGTGCAGGTAG | down | comp19528_c1 | 6-phosphofructokinase | up |
| dre-miR-301c-3p_R+1 | CAGTGCAATAGTATTGTCATAGC | down | comp19528_c1 | 6-phosphofructokinase | up |
| ssa-miR-20a-5p | TAAAGTGCTTATAGTGCAGGTAG | down | comp19528_c1 | 6-phosphofructokinase | up |
| ssa-miR-301a-3p | CAGTGCAATAGTATTGTCATAGC | down | comp19528_c1 | 6-phosphofructokinase | up |
| PC-3p-36625_60 | TGCTTTGAGAGGCTAGTCATGA | down | comp19575_c0 | Rab11 family-interacting protein 3/4 | up |
| dre-miR-338_R-1 | TCCAGCATCAGTGATTTTGTT | down | comp19639_c0 | - | up |
| ppy-miR-338-3p_R-1 | TCCAGCATCAGTGATTTTGTT | down | comp19639_c0 | - | up |
| ccr-miR-17-5p | CAAAGTGCTTACAGTGCAGGTAG | down | comp19837_c0 | nuclear receptor, subfamily 1, group C, member 1 | up |
| ssa-miR-20a-5p | TAAAGTGCTTATAGTGCAGGTAG | down | comp19837_c0 | nuclear receptor, subfamily 1, group C, member 1 | up |
| ssa-miR-16a-3p_R+1_2ss10TA11TC | CCAGTATTGACCGTGCTGCTGAA | down | comp19840_c0 | transferrin receptor | up |
| ssa-miR-20a-5p | TAAAGTGCTTATAGTGCAGGTAG | down | comp19840_c0 | transferrin receptor | up |
| ssa-miR-200b-5p | CATCTTACCTGACAGTGCTGGA | up | comp19895_c0 | receptor tyrosine-protein kinase erbB-3 | down |
| dre-miR-301c-3p_R+1 | CAGTGCAATAGTATTGTCATAGC | down | comp19946_c2 | - | up |
| ssa-miR-301a-3p | CAGTGCAATAGTATTGTCATAGC | down | comp19946_c2 | - | up |
| ccr-miR-17-5p | CAAAGTGCTTACAGTGCAGGTAG | down | comp19983_c0 | myosin heavy chain | up |
| ssa-miR-20a-5p | TAAAGTGCTTATAGTGCAGGTAG | down | comp19983_c0 | myosin heavy chain | up |
| ssa-miR-16a-3p_R+1_2ss10TA11TC | CCAGTATTGACCGTGCTGCTGAA | down | comp20010_c0 | - | up |
| PC-5p-48126_34 | CATGACTAGCCTCTCAAAGCAC | down | comp20056_c0 | inorganic phosphate transporter, PiT family | up |
| dre-miR-301c-3p_R+1 | CAGTGCAATAGTATTGTCATAGC | down | comp20056_c0 | inorganic phosphate transporter, PiT family | up |
| dre-miR-338_R-1 | TCCAGCATCAGTGATTTTGTT | down | comp20056_c0 | inorganic phosphate transporter, PiT family | up |
| ppy-miR-338-3p_R-1 | TCCAGCATCAGTGATTTTGTT | down | comp20056_c0 | inorganic phosphate transporter, PiT family | up |
| ssa-miR-301a-3p | CAGTGCAATAGTATTGTCATAGC | down | comp20056_c0 | inorganic phosphate transporter, PiT family | up |
| hsa-miR-3618_1ss21GA | TGTCTACATTAATGAAAAGAAC | down | comp21298_c0 | - | up |
| ccr-miR-143_R+1_1ss20TA | TGAGATGAAGCACTGTAGCAC | up | comp2836_c0 | - | down |
| ssa-miR-200b-5p | CATCTTACCTGACAGTGCTGGA | up | comp2836_c0 | - | down |
| ccr-miR-187 | TCGTGTCTTGTGTTGCAGCCAGT | up | comp3100_c0 | - | down |
| ccr-miR-17-5p | CAAAGTGCTTACAGTGCAGGTAG | down | comp5908_c0 | L-lactate dehydrogenase | up |
| ssa-miR-20a-5p | TAAAGTGCTTATAGTGCAGGTAG | down | comp5908_c0 | L-lactate dehydrogenase | up |
| dre-miR-27b-3p_R-1 | TTCACAGTGGCTAAGTTCTGC | up | comp7480_c0 | aldehyde reductase | down |
| ccr-miR-17-5p | CAAAGTGCTTACAGTGCAGGTAG | down | comp7530_c0 | zinc finger and BTB domain-containing protein 16 | up |
| ssa-miR-20a-5p | TAAAGTGCTTATAGTGCAGGTAG | down | comp7530_c0 | zinc finger and BTB domain-containing protein 16 | up |
| dre-miR-27b-3p_R-1 | TTCACAGTGGCTAAGTTCTGC | up | comp7697_c0 | histone H2A | down |
| ccr-miR-17-5p | CAAAGTGCTTACAGTGCAGGTAG | down | comp7721_c0 | angiopoietin-like 4 | up |
| ssa-miR-20a-5p | TAAAGTGCTTATAGTGCAGGTAG | down | comp7721_c0 | angiopoietin-like 4 | up |
| ssa-miR-16a-3p_R+1_2ss10TA11TC | CCAGTATTGACCGTGCTGCTGAA | down | comp8016_c0 | - | up |
| PC-3p-36625_60 | TGCTTTGAGAGGCTAGTCATGA | down | comp8203_c0 | - | up |
| dre-miR-338_R-1 | TCCAGCATCAGTGATTTTGTT | down | comp8626_c0 | - | up |
| ppy-miR-338-3p_R-1 | TCCAGCATCAGTGATTTTGTT | down | comp8626_c0 | - | up |
| dre-miR-27b-3p_R-1 | TTCACAGTGGCTAAGTTCTGC | up | comp9504_c0 | purinergic receptor P2Y, G protein-coupled, 5 | down |
| dre-miR-301c-3p_R+1 | CAGTGCAATAGTATTGTCATAGC | down | comp9770_c0 | transcription factor AP-1 | up |
| ssa-miR-301a-3p | CAGTGCAATAGTATTGTCATAGC | down | comp9770_c0 | transcription factor AP-1 | up |
| dre-miR-27b-3p_R-1 | TTCACAGTGGCTAAGTTCTGC | up | comp9860_c1 | - | down |
| dre-miR-27b-3p_R-1 | TTCACAGTGGCTAAGTTCTGC | up | comp9877_c0 | histamine N-methyltransferase | down |
| ssa-miR-16a-3p_R+1_2ss10TA11TC | CCAGTATTGACCGTGCTGCTGAA | down | comp9951_c0 | - | up |

Table S11 Primers used in this study.

| Name of genes | Forward primer (5′-3′) | Reverse primer (5′-3′) |
| --- | --- | --- |
| 6-phosphofructokinase (PFKL) | GGCTTACGAGGGTGTTCTCC | CGCCTTTTGGTTCCAGATGC |
| hexokinase (HK) | CAGTACGACCGAAACGTGGA | GTCCCCTGAACAAGAAGCCA |
| lactate dehydrogenase (LDH) | CCCCAACTGTATCATCCTGG | TGGGTGCAGACCCAGTTTC |
| phosphoglycerate mutase (PGAM) | ACCGAGGACCAGCTACCTT | CCCTGTTTAATCTGAGGTACGA |
| lipoprotein lipase (LPL) | TGGCTACCACAGGGTTGAGA | CAGCTCAGACACACACCCTT |
| apoptosis regulator BCL-2(BCL2) | TTTTCGAGTTTGGCGCGATG | CCACAAAGGCATCCCATCCT |
| von Hippel-Lindau disease tumor supressor(Vhl) | AGACGGATGACCCGATGTTG | GCACACTAGCTTCCTCACCA |
| transferrin receptor(TFRC) | CGCAACAGGAACCAAACAGG | AGAACCGCAGCTAAACCGAA |
| MFS transporter, SP family, solute carrier family 2 (facilitated glucose transporter), member 1(SLC2A1) | TCCTGGTGGGGCTTTACTCT | CACGGAGGTTAGTGGGTGAG |
| death-associated protein kinase (DAPK) | TAGAAGACAGTATGCTCGCCG | GTCGCTCTCACAGTCCCTTG |
| transcription factor AP-1(JUN) | GCGGAGCACGTAAATCACAC | TCCTGGTCCTCCATGTCGAT |
| angiopoietin-like 4(ANGPTL4) | CTCCGTCGTGACCAGTGAAA | TTCCTCGTTGTTGGCCTTGA |
| carbonic anhydrase (CA) | GGGTCTCTAACAACTCCGCC | TGTCCACCTTTCAGCGTCTC |
| activating transcription factor 2(ATF2) | CACCCCCTGAACACCTACAC | GTCTGTTGAGCGTTTGCGTT |
| cAMP response element modulator(CREM) | CTTGAAAACCGTGTTGCCGT | CGGTTTTGCGGCAGTACAAG |
| insulin receptor substrate(IRS) | AGTTGCAGTGGCTCTGTCTC | ATTGCTCCGGTTGGATGTGT |
| dual specificity phosphatase (DUSP8) | AGCAAGGCAGAGGAACCAAA | GGGAGATGGCAGCTTGATGT |
| serine/threonine kinase (Akt) | TTGCGAACTTTTTCCAGCCAG | GCCTTAACACAGGTAGGGAGG |
| vascular endothelial growth factor (VEGF) | GCTGCTGCAATGACGAGATG | CGTTTGATGCGCATGGGTTT |
| erythropoietin(EPO) | CAGTTCCTCTGACCAGCGTG | AGAGTTGGTGATGGACGCAC |
| 5'-AMP-activated protein kinase, regulatory gamma subunit(PRKAG2) | GCCCCTCCCCTTTACACATC | AATATCGCAGCTCGGCTCTC |

| Name | Primer (5′-3′) |
| --- | --- |
| ola-miR-210-5p RT | CTCAACTGGTGTCGTGGAGTCGGCAATTCAGTTGAGCAGTGTGC |
| ola-miR-210-5p F | ACACTCCAGCTGGGAGCCACTGACTAACGCAC |
| mir-R210 | TGTCGTGGAGTCGGCAATTC |
| Name | Primer (5′-3′) |
| ccr-miR-17-5p RT | CTCAACTGGTGTCGTGGAGTCGGCAATTCAGTTGAGCTACCTGC |
| ccr-miR-17-5p F | ACACTCCAGCTGGGCAAAGTGCTTACAGTGCAG |
| mir-R17 | TGTCGTGGAGTCGGCAATTC |
| Name | Primer (5′-3′) |
| dre-miR-301c-3p RT | CTCAACTGGTGTCGTGGAGTCGGCAATTCAGTTGAGGCTATGAC |
| dre-miR-301c-3p F | ACACTCCAGCTGGGCAGTGCAATAGTATTGTCA |
| mir-R301c | TGTCGTGGAGTCGGCAATTC |
| Name | Primer (5′-3′) |
| ssa-miR-16a-3p RT | CTCAACTGGTGTCGTGGAGTCGGCAATTCAGTTGAGTTCAGCAG |
| ssa-miR-16a-3p F | ACACTCCAGCTGGGCCAGTATTGACCGTGCTGC |
| mir-R16a | TGTCGTGGAGTCGGCAATTC |
| Name | Primer (5′-3′) |
| PC-5p-83983 RT | CTCAACTGGTGTCGTGGAGTCGGCAATTCAGTTGAGTACCCGGG |
| PC-5p-83983 F | ACACTCCAGCTGGGGCTTATTGTGAGTCCCG |
| mir-R83983 | TGTCGTGGAGTCGGCAATTC |
| Name | Primer (5′-3′) |
| ssa-miR-20a-5p RT | CTCAACTGGTGTCGTGGAGTCGGCAATTCAGTTGAGCTACCTGC |
| ssa-miR-20a-5p F | ACACTCCAGCTGGGTAAAGTGCTTATAGTGCAG |
| mir-R20a | TGTCGTGGAGTCGGCAATTC |
| Name | Primer (5′-3′) |
| dre-miR-338 RT | CTCAACTGGTGTCGTGGAGTCGGCAATTCAGTTGAGAACAAAAT |
| dre-miR-338 F | ACACTCCAGCTGGGTCCAGCATCAGTGATTT |
| mir-R338 | TGTCGTGGAGTCGGCAATTC |
| Name | Primer (5′-3′) |
| ccr-miR-143 RT | CTCAACTGGTGTCGTGGAGTCGGCAATTCAGTTGAGGTGCTACA |
| ccr-miR-143 F | ACACTCCAGCTGGGTGAGATGAAGCACTGTA |
| mir-R143 | TGTCGTGGAGTCGGCAATTC |
| Name | Primer (5′-3′) |
| PC-3p-36625 RT | CTCAACTGGTGTCGTGGAGTCGGCAATTCAGTTGAGTCATGACT |
| PC-3p-36625 F | ACACTCCAGCTGGGTGCTTTGAGAGGCTAGTC |
| mir-R36625 | TGTCGTGGAGTCGGCAATTC |
| Name | Primer (5′-3′) |
| hsa-miR-3618 RT | CTCAACTGGTGTCGTGGAGTCGGCAATTCAGTTGAGGTTCTTTTCA |
| hsa-miR-3618 F | ACACTCCAGCTGGGTGTCTACATTAATGAA |
| mir-R3618 | TGTCGTGGAGTCGGCAATTC |
| Name | Primer (5′-3′) |
| dre-miR-27b-3p RT | CTCAACTGGTGTCGTGGAGTCGGCAATTCAGTTGAGGCAGAACT |
| dre-miR-27b-3p F | ACACTCCAGCTGGGTTCACAGTGGCTAAGTT |
| mir-R27b | TGTCGTGGAGTCGGCAATTC |
| Name | Primer (5′-3′) |
| ppy-miR-338-3p_R-1 RT | CTCAACTGGTGTCGTGGAGTCGGCAATTCAGTTGAGAACAAAAT |
| ppy-miR-338-3p_R-1 F | ACACTCCAGCTGGGTCCAGCATCAGTGATTT |
| mir-R338 | TGTCGTGGAGTCGGCAATTC |
| Name | Primer (5′-3′) |
| ssa-miR-301a-3p RT | CTCAACTGGTGTCGTGGAGTCGGCAATTCAGTTGAGGCTATGAC |
| ssa-miR-301a-3p F | ACACTCCAGCTGGGCAGTGCAATAGTATTGTCA |
| mir-R301a | TGTCGTGGAGTCGGCAATTC |
| Name | Primer (5′-3′) |
| u6-RT | AAAGATGGAACGCTTCACG |
| u6-F | ACTAAAATTGGAACGATACAGAGA |
| u6-R | AAAGATGGAACGCTTCACG |
